# Meroterpenoids and Isocoumarinoids from a *Myrothecium* Fungus Associated with *Apocynum venetum*

**DOI:** 10.3390/md16100363

**Published:** 2018-10-01

**Authors:** Yanchao Xu, Cong Wang, Haishan Liu, Guoliang Zhu, Peng Fu, Liping Wang, Weiming Zhu

**Affiliations:** 1 State Key Laboratory of Functions and Applications of Medicinal Plants, Guizhou Medical University, Guiyang 550014, China; m18586818694@163.com; 2 Key Laboratory of Marine Drugs, Ministry of Education of China, School of Medicine and Pharmacy, Ocean University of China, Qingdao 266003, China; wangcong123206@163.com(C.W.); liuhaishan_229@outlook.com (H.L.); guoliangzhu2015@hotmail.com (G.Z.); fupeng@ouc.edu.cn (P.F.); 3 Laboratory for Marine Drugs and Bioproducts, Qingdao National Laboratory for Marine Science and Technology, Qingdao 266003, China; 4 Guangxi Key Laboratory of Chemistry and Engineering of Forest Products, School of Chemistry and Chemical Engineering, Guangxi University for Nationalities, Nanning 530006, China

**Keywords:** endophytic fungus, *Myrothecium* sp., meroterpenoids, isocoumarinoids, α-glucosidase inhibitors, salt-resistant plant, *Apocynum venetum*

## Abstract

Four new meroterpenoids **1**–**4** and four new isocoumarinoids **5**–**8**, along with five known isocoumarinoids (**9**–**13**), were isolated from the fungus *Myrothecium* sp. OUCMDZ-2784 associated with the salt-resistant medicinal plant, *Apocynum venetum* (Apocynaceae). Their structures were elucidated by means of spectroscopic analysis, X-ray crystallography, ECD spectra and quantum chemical calculations. Compounds **1**–**5**, **7**, **9** and **10** showed weak α-glucosidase inhibition with the IC_50_ values of 0.50, 0.66, 0.058, 0.20, 0.32, 0.036, 0.026 and 0.37 mM, respectively.

## 1. Introduction

Since the discovery of penicillin, fungi have been an important source of lead compounds for drug development, which have provided a lot of attractive natural products (NPs) with different biological activities [1,2,3]. With the increase of study on the terrestrial fungal NPs, more and more known compounds were isolated repeatedly. Therefore, many researchers turned their attention to the fungi isolated from specific habitats, such as the marine-derived fungi [4,5,6,7] and the fungi associated with the plants or animals [8,9,10,11]. 

As part of our ongoing studies to search for bioactive NPs from fungi derived from special niche [12,13,14,15,16], we screened the fungus *Myrothecium* sp. OUCMDZ-2784 which is associated with the salt-resistant plant *A. venetum* (Apocynaceae) growing in the Yellow River Delta, a traditional Chinese medicine used for treatment of hypertension [17] and heart failure [18]. *Myrothecium* sp. has been reported to produce trichothecenes [19], sesquiterpenes [20,21], diterpenes [22] and cyclopeptides [23] with cytotoxic and antibacterial activities. The ethyl acetate (EtOAc) extract of the fermentation of *Myrothecium* sp. OUCMDZ-2784 showed 75% inhibition of α-glucosidase at 286 μg/mL. Chemical study resulted in the isolation and identification of four new meroterpenoids, myrothecisins A–D (**1**–**4**) and four new isocoumarinoids, myrothelactones A–D (**5**–**8**), together with five known isocoumarinoids that were identified as tubakialactone B (**9**) [24], acremonone G (**10**) [25], 6,8-dihydroxy-3-methylisocoumarin (**11**) [26], 3,4-dimethyl-6,8-dihydroxyisocoumarin (**12**) [27] and sescandelin B (**13**) [28], respectively by comparing ^1^H and ^13^C NMR spectra (Figure S57, Table S1) as well as ESIMS spectra (Figure S59) with those reported. 

## 2. Results and Discussion

**Figure 1:**
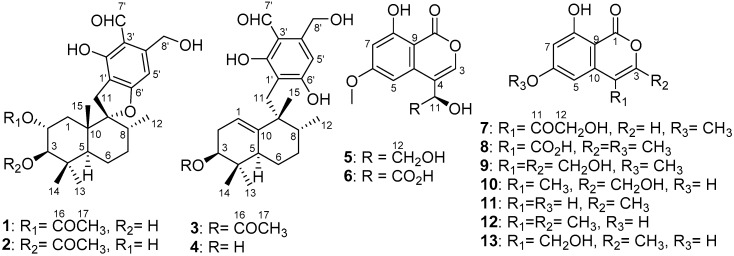
Structures **1**–**13** isolated from *Myrothecium* sp. OUCMDZ-2784.

**Figure 2:**
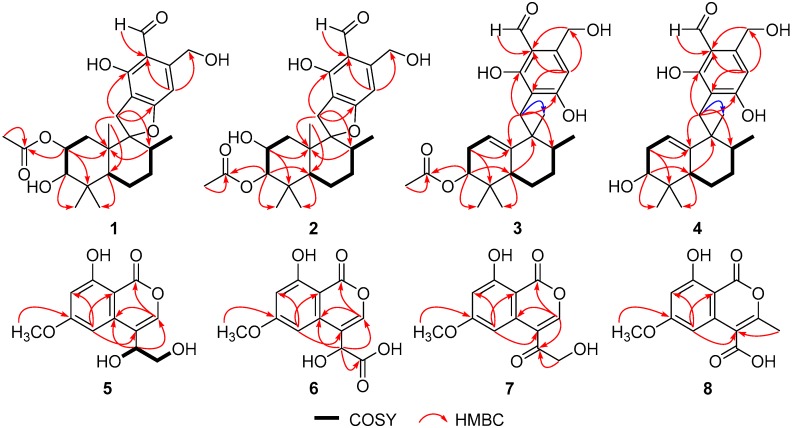
Key homonuclear correlation spectroscopy (COSY) and key HMBC correlations for **1**–**8**.

**Figure 3:**
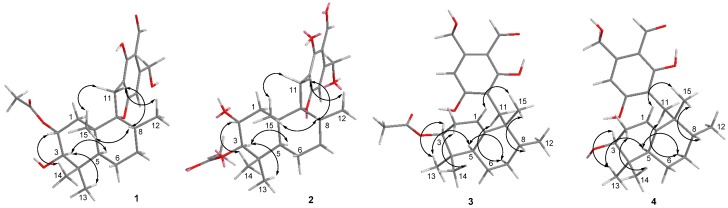
NOESY correlations for **1**–**4**.

**Figure 4:**
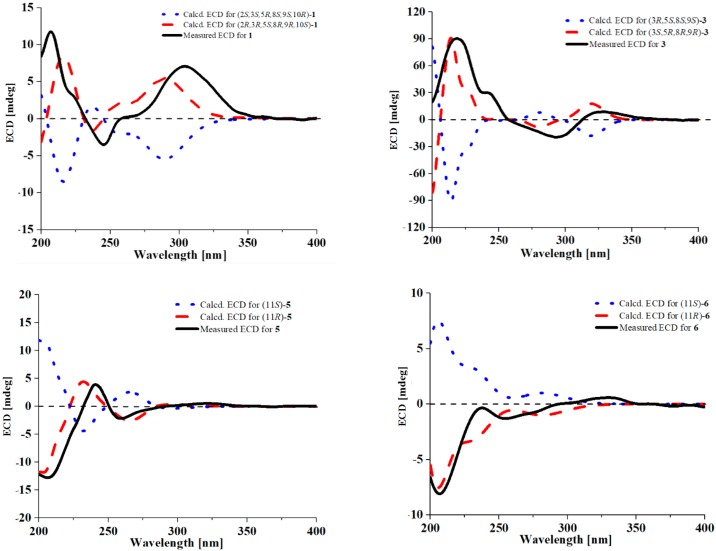
Measured and calculated ECD spectra for **1**, **3**, **5** and **6**.

Myrothecisin A (**1**) was isolated as a pale-yellow oil. Its molecular formula was assigned as C_25_H_34_O_7_ by the HRESIMS peak at *m/z* 469.2188 [M + Na]^+^ (Figure S58A), indicating nine degrees of unsaturation. The ^13^C NMR (Figure S6) spectrum of **1** showed 25 signals that were classified by DEPT (Figure S7) and HSQC (Figure S8) as an aldehyde carbonyl carbon (δ_C_ 193.8), one acyl carbonyl carbon (δ_C_ 170.3), five sp^2^ non-protonated carbons (δ_C_ 167.3, 159.8, 149.5, 112.3, 111.2) and three sp^3^ non-protonated carbons (δ_C_ 98.8, 42.8, 39.2), one sp^2^ methine (δ_C_ 101.5) and four sp^3^ methines (δ_C_ 78.2, 71.4, 45.6, 36.0), five sp^3^ methylenes (δ_C_ 60.5, 35.1, 30.6, 30.4, 20.7) and five methyl carbons (δ_C_ 28.7, 21.3, 17.0, 16.6, 15.2) (Table 1). The ^1^H (Figure S5) and HSQC NMR showed the singlet signals at δ_H_ 10.06 and 6.56 for an aldehyde proton and an aromatic proton, respectively. The key HMBC (Figure S10) correlations from H-7′ (δ_H_ 10.06) to C-2′/C-3′/C-4′, 2′-OH (δ_H_ 12.16) to C-1′/C-2′/C-3′, H-5′ (δ_H_ 6.56) to C-1′/C-4′/C-6′/C-8′, H-8′ (δ_H_ 4.74) to C-3′/C-4′/C-5′ and 8′-OH (δ_H_ 5.44) to C-4′/C-8′ suggested a penta-substituted benzene ring (Figure 2). The COSY (homonuclear correlation spectroscopy) correlations from H-1 through H-2 to H-3 and H-5 through H-6, H-7 and H-8 to H-12 (Figure 1 and Figure S9), along with the key HMBC correlations from H-2 to C-4/C-10/C-16, H-3 to C-5/C-13/C-14, 3-OH to C-2/C-3/C-4, H-1 to C-2/C-3/C-10/C-15, H-15 to C-1/C-5/C-9/C-10, H-5 to C-3/C-4/C-6/C-13, H-6 to C-5, H-7 to C-8, H-13 to C-3/C-5/C-14, H-14 to C-3/C-5/C-13, H-12 to C-7/C-8/C-9 and H-17 to C-16 revealed a sesquiterpene fragment (Figure 2). The connection of the above-mentioned two fragments were confirmed by the key HMBC correlations from H-11 to C-8/C-9/C-10/C-1′/C-2′/C-6′ (Figure 2) [29]. The relative configuration of **1** was determined by the NOESY correlations from H-8 to H-11 and H-15, H-3 to H-5 and H-13, H-2 to H-14 and H-15 and H-11 to H-15 (Figure 3 and Figure S11). The absolute configuration of **1** was determined by calculation of electronic circular dichroism (ECD) using time-dependent density functional theory (TDDFT) (Figure S1) [30,31] and the measured ECD spectrum of **1** matched well with the calculated ECD spectrum for (2*R*,3*R*,5*S*,8*R*,9*R*,10*S*)-**1** (Figure 4). 

**Figure 5:**
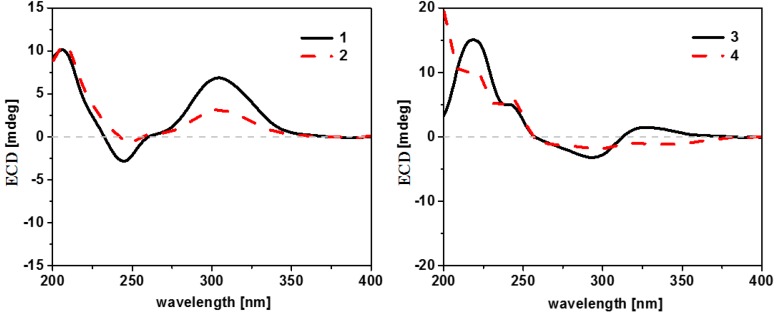
ECD spectra for **1**−**4**.

The molecular formula of **2** was also determined as C_25_H_34_O_7_ by the HRESIMS peak at *m/z* 469.2200 [M + Na]^+^ (Figure S58B), implying that **2** is an isomer of **1**. Comparison of its ^1^H and ^13^C NMR spectra (Figures S12–S16) with those of **1** revealed that the acetyloxy group in **2** was on C-3. This was confirmed by the HMBC (Figure S17) correlation from H-3 (δ_H_ 4.29) to C-16 (δ_C_ 170.3) (Figure 2). The similar NOESY correlations (Figure 3 and Figure S18) suggested that **2** has the same relative configuration as **1**. The similarity of ECD curves between **2** and **1** (Figure 5) indicated the same absolute configurations of its stereogenic carbons. Therefore **2** was named myrothecisin B.

Compound **3** was also obtained as a pale-yellow oil. Its molecular formula was determined as C_25_H_34_O_6_ according to the HRESIMS peak at *m/z* 453.2238 [M + Na]^+^ (Figure S58C). The ^13^C NMR (Figure S20) spectrum of **3** showed one aldehyde carbonyl carbon (δ_C_ 193.3), one acyl carbonyl carbon (δ_C_ 169.8), six sp^2^ non-protonated carbons (δ_C_ 164.6, 164.5, 145.4, 143.9, 111.4, 110.2) and two sp^3^ non-protonated carbons (δ_C_ 43.7, 35.6), two sp^2^ methines (δ_C_ 114.7, 107.5) and three sp^3^ methines (δ_C_ 75.6, 43.3, 43.1), five sp^3^ methylenes (δ_C_ 59.9, 30.2, 28.0, 26.7, 23.5) and five methyl carbons (δ_C_ 25.0, 23.0, 21.0, 17.1, 17.1) (Table 1). Analysis of its 1D and 2D NMR (Figures S19–S24) data revealed the presence of a substituted benzene ring and a sesquiterpene unit, indicating **3** was an analogue of **1** and **2**. Comparison of the ^1^H and ^13^C NMR spectra with those of **1** and **2** suggested a same pentasubstituted benzene ring. The structure of the sesquiterpene unit was slightly modified and was determined by the COSY (Figure S23) correlations from H-1 through H-2 to H-3 and H-5 through H-6, H-7 and H-8 to H-12 and the key HMBC (Figure S24) correlations from H-3 to C-2/C-4/C-10/C-13/C-14, H-17 to C-16, H-1 to C-3/C-9, H-15 to C-10, H-8 to C-9, H-5 to C-9/C-13, H-12 to C-7 and H-2 to C-4/C-10 (Figure 2). The HMBC correlations from H_2_-11 (δ_H_ 2.68/2.53) to C-8/C-10/C-15/C-2′/C-6′ (Figure 2) confirmed the connection between the sesquiterpene fragment and the benzene ring. The relative configuration of **3** was determined by the NOESY (Figure S25) correlations from H-13 to H-3 and H-5, H-8 to H-15, as well as H-5 to H-11 (Figure 3). The absolute configuration was determined as (3*S*,5*R*,8*R*,9*R*)- by comparison of the calculated and experimental ECD spectra (Figure 4 and Figure S2). Therefore **3** was named myrothecisin C.

The molecular formula of **4** was assigned as C_23_H_32_O_5_ by the HRESIMS peak at *m/z* 411.2139 [M + Na]^+^ (Figure S58D), which was C_2_H_2_O less than that of **3**. The similarity of the UV and NMR data between **3** and **4** (Table 1) suggested that **4** possesses the same skeleton as **3**. Careful comparison of their ^1^H and ^13^C NMR spectra (Figures S26–S31) showed that the acetyloxy group (δ_C_ 21.0/δ_H_ 1.98 and δ_C_ 169.8) in **3** was replaced by a hydroxy group (δ_H_ 4.13) in **4** (Table 1). The NOESY data (Figure 3 and Figure S32) suggested that **4** has the same relative configuration as **3**. The ECD Cotton effects of **4** were nearly identical to those of **3** (Figure 5), indicating the same absolute configurations of the corresponding stereogenic carbons. Thus, **4** was named myrothecisin D.

**Figure 6:**
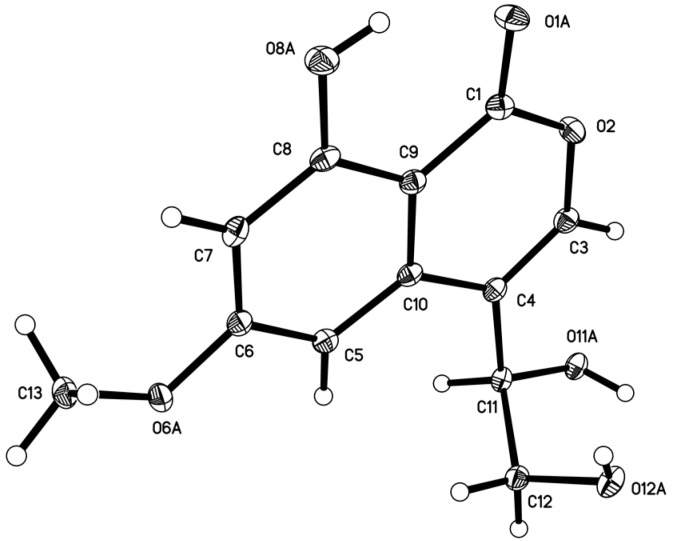
ORTEP diagram of **5**.

Compound **5** was obtained as a colorless crystal with the molecular formula C_12_H_12_O_6_ from the HRESIMS peak at *m/z* 251.0563 [M − H]^−^ (Figure S58E). The ^1^H NMR spectrum showed two *meta*-coupled aromatic protons at δ_H_ 6.78 (d, *J* = 2.2 Hz) and δ_H_ 6.63 (d, *J* = 2.2 Hz) (Table 2, Figure S33), indicating the presence of a tetra-substituted benzene ring. The ^13^C (Figure S34) NMR spectrum showed 12 carbon signals that were classified by DEPT (Figure S35) and HSQC (Figure S36) spectra as six sp^2^ non-protonated carbons (δ_C_ 166.4, 165.4, 163.4, 137.6, 118.6, 99.9), three sp^2^ methines (δ_C_ 143.1, 100.5, 100.4) and one sp^3^ methine (δ_C_ 68.8), one sp^3^ methylene (δ_C_ 64.8) and one methoxy group (δ_C_ 56.0) (Table 2). The key HMBC correlations (Figure 2 and Figure S38) from CH_3_O-6 to C-6, HO-11 to C-4/C-12, H-11 to C-3/C-10, H-3 to C-1/C-10, H-5 to C-4/C-7/C-9 and H-7 to C-9 along with the continuous COSY correlations of HO-11 (δ_H_ 5.50)/H-11 (δ_H_ 4.66)/H-12 (δ_H_ 3.51, 3.62)/HO-12 (δ_H_ 4.81) (Figure S37) revealed that **5** possesses a 4,6,8-trisubstituted isocoumarin skeleton with a hydroxy, a methoxy and a 1,2-dihydroxy ethyl at C-8, C-6 and C-4, respectively. The structure of **5** was further confirmed by X-ray crystallography (Figure 6). Because the value of the Flack parameter [−0.2(2)] was large, the absolute configuration determined by X-ray crystallography was not reliable. Thus, the ECD calculation method was used to further confirm the absolute configuration of C-11 of **5** as 11*R*- (Figure 4 and Figure S3). Consequently, **5** was named myrothelactone A.

Compound **6** was obtained as a white powder. Its molecular formula was determined as C_12_H_10_O_7_ based on the HRESIMS peak at *m/z* 265.0355 [M − H]^−^ (Figure S58F). The UV and ^13^C NMR data of **6** (Table 2) were similar to those of **5**, indicating that they have the same isocoumarin scaffold. Comparison of their ^1^H and ^13^C data (Figures S39–S43) indicated that the hydroxymethyl group (δ_C/H_ 64.8/3.62&3.51, δ_H_ 4.81) in **5** was replaced by the carboxyl group (δ_C_ 173.4). This change was verified by the key HMBC (Figure S44) correlations from H-11 to C-3/C-10/C-12. The absolute configuration of C-11 of **6** was determined as 11*R*- by comparison of the calculated and experimental ECD spectra (Figure 4 and Figure S4). Therefore, **6** was name myrothelactone B.

Compound **7** was obtained as a white powder. Its molecular formula was determined as C_12_H_10_O_6_ according to its HRESIMS peak at *m/z* 249.0408 [M – H]^−^ (Figure S58G), which was only two hydrogen atoms less than that of **5**. The difference observed in the NMR spectra of **7** and **5** was that the signals for hydroxymethine (δ_C/H_ 68.8/4.66) in **5** were replaced by the signal of a carbonyl group (δ_C-11_ 198.3) in **7** (Table 2, Figures S45–S49). The HMBC (Figure S50) correlations from H-3 (δ_H_ 8.47) and H-12 (δ_H_ 4.57) to C-11 further confirmed the structure of **7** which was name myrothelactone C (Figure 2).

The molecular formula of compound **8** was determined as C_12_H_10_O_6_ on the basis of its HRESIMS peak at *m/z* 249.0407 [M − H]^−^ (Figure S58H), which is an isomer of **7**. Analysis of its ^1^H and ^13^C NMR spectra showed that **8** also had the same isocoumarin scaffold, whose difference is the replacement of carbonyl (δ_C_ 198.3), hydroxymethyl (δ_C_ 65.9, δ_H_ 4.57/5.28) and sp^2^ methine (δ_C_ 153.1, δ_H_ 8.47) signals in **7** by two sp^2^ non-protonated carbons (δ_C_ 173.9, 151.0) and methyl (δ_C_ 17.9, δ_H_ 2.29) signals in **8** (Table 2, Figures S51–S55). The HMBC correlations from H-12 (δ_H_ 2.29) to C-3 (δ_C_ 151.0) and C-4 (δ_C_ 118.7) suggested the methyl substitution at C-3 (Figure 2 and Figure S56). The chemical shift of the carboxyl signal (δ_C_ 173.9) together with 2D NMR data indicated the carboxyl substitution at C-4. The structure of myrothelactone D (**8**) was therefore determined (Figure 2).

The α-glucosidase inhibitory activity of **1**–**13** was preliminarily investigated. Compounds **1**–**5**, **7**, **9** and **10** exhibited inhibitory activity against the human-sourced α-glucosidase recombinant expressed in *Saccharomyces cerevisiae* [31,32,33] with IC_50_ values of 0.50, 0.66, 0.058, 0.20, 0.32, 0.036, 0.026 and 0.37 mM, while the IC_50_ value of positive control acarbose was 0.47 mM. Due to the low activity, the deeper investigation of the mechanism and type of enzymatic inhibition as well as the binding mode were not done.

## 3. Experimental Section

### 3.1. General Experimental Procedures

Optical rotations were measured using a JASCO P-1020 digital polarimeter (JASCO Corporation, Tokyo, Japan). UV spectra were obtained on a Beckman DU 640 spectrophotometer (Beckman Coulter, Inc., Brea, CA, USA). CD data were performed using a JASCO J-815 spectropolarimeter (JASCO Corporation, Tokyo, Japan). IR spectra were obtained on a Nicolet Nexus 470 spectrophotometer (Thermo Nicolet Corporation, Madison, WI, USA) as KBr discs. NMR spectra were recorded on a Varian System 500 spectrometer (Varian, Palo Alto, CA, USA) or a Bruker Avance 600 spectrometer (Bruker, Fallanden, Switzerland) using residual solvent signals for referencing and chemical shifts were recorded as δ values. HRESIMS spectra were measured using the Q-TOF ULTIMA GLOBAL GAA076 LC mass spectrometer (Waters Asia, Ltd., Singapore). Semi-preparative HPLC was performed using an ODS column (YMC-pack ODS-A, 10 mm × 250 mm, 5 μm, 4.0 mL/min, Kyoto, Japan). TLC and column chromatography (CC) were performed on plates pre-coated with silica gel GF_254_ (10–40 µm, Qingdao Marine Chemical Factory, Qingdao, China) and Sephadex LH-20 (Amersham Biosciences, Uppsala, Sweden), respectively. Vacuum-liquid chromatography (VLC) utilized silica gel H (Qingdao Marine Chemical Factory).

### 3.2. Collection and Phylogenetic Analysis

The fungus OUCMDZ-2784 was isolated from *Apocynum venetum* (Apocynaceae) collected from the estuary of Yellow River, Dongying, China. The leaves of the plant were washed with tap water and sterile distilled water in sequence. Then, it was cut into small pieces, which were then put into a centrifuge tubes filled with different concentrations of sucrose solution. These tubes were centrifuged at 1200 rpm for 20 min. Four zones were separated by improved discontinuous sucrose gradient centrifugation. The interface between the third and the fourth bands was deposited on a PDA (200 g potato, 20 g glucose, 20 g agar per liter of sea water) plate containing chloramphenicol (100 μg/mL) as a bacterial inhibitor and was then cultured at 28 °C for 3 days. A single colony was transferred to PDA agar media and was identified as *Myrothecium* sp. according to its morphological characteristics and 18S rRNA gene sequences (GenBank accession No. KF977010).

### 3.3. Cultivation and Extraction

Fungus OUCMDZ-2784 was prepared on PDA agar medium. Spores were incubated at 28 °C for 48 h on a rotary shaker with shaking at 120 rpm in a 500 mL cylindrical flask containing 150 mL liquid medium (20 g maltose, 20 g mannitol, 10 g glucose, 3 g yeast extract, 10 g monosodium glutamate per liter of sea water). The cultures were transferred to 350 × 1000 mL Erlenmeyer flasks and each containing 300 mL liquid fermentation media (1 g peptone, 10g soluble starch per liter of sea water, pH 7.0). The flasks were incubated at room temperature under static conditions for 30 days. The cultures were extracted three times by EtOAc and the combined EtOAc extracts were dried in vacuo to yield 20.1 g of extract.

### 3.4. Purification

The extract (20.1 g) was fractionated by VLC, eluting with a step gradient of CH_2_Cl_2_-petroleum ether (50–100%) and MeOH-CH_2_Cl_2_ (0–50%) and five fractions (Fr.1–Fr.5) were collected. Fraction 2 (3.2 g) was subjected to Sephadex LH-20 chromatography eluting with CH_2_Cl_2_/MeOH (1:1) to afford three subfractions (Fr.2.1–Fr.2.3). Fr.2.1 (1.0 g) was further purified by HPLC on an ODS column (80% MeOH/H_2_O) to give compounds **1** (25.2 mg, *t*_R_ 6.3 min) and **3** (30.1 mg, *t*_R_ 10.2 min). Fr.2.2 (50.2 mg) was purified by HPLC on an ODS column (60% MeOH/H_2_O) to yield compounds **11** (3.5 mg, *t*_R_ 10.4 min) and **8** (3.3 mg, *t*_R_ 15.2 min). Fr.2.3 (46.3 mg) was purified by HPLC on an ODS column (60% MeOH/H_2_O) to yield compounds **12** (5.1 mg, *t*_R_ 12.6 min) and **13** (12.0 mg, *t*_R_ 16.4 min). Fraction 3 (3.8 g) was separated into three subfractions (Fr.3.1–Fr.3.3) by Sephadex LH-20 eluting with MeOH-CH_2_Cl_2_ (1:1). Fr.3.1 (0.5 g) was purified by semi preparative HPLC on an ODS column (85% MeOH/H_2_O) to yield compound **2** (36.2 mg, *t*_R_ 10.2 min). Fr.3.2 (1.1 g) was separated by silica gel VLC column eluting with CH_2_Cl_2_-petroleum (2:1) to yield compounds **10** (100.3 mg) and **5** (200.8 mg). Fr.3.3 (0.5 g) was further purified by Sephadex LH-20 eluting with MeOH to yield compound **9** (15.3 mg). Fraction 4 (1.8 g) was separated into three subfractions (Fr.4.1–Fr.4.3) by Sephadex LH-20 eluting with MeOH. Fr.4.1 (0.6 g) was further purified by semi preparative HPLC on an ODS column (85% MeOH/H_2_O) to yield compound **4** (34.3 mg, *t*_R_ 12.2 min). Fr.4.2 (0.2 g) was further purified by semi preparative HPLC on an ODS column (50% MeOH/H_2_O) to yield compound **7** (6.0 mg, *t*_R_ 12.7 min). Fr.4.3 (0.3 g) was purified by HPLC on an ODS column (40% MeOH/H_2_O) to yield compound **6** (5.8 mg, *t*_R_ 10.6 min).

*Myrothecisin A* *(***1***)*: pale yellow oil; ${\left[\alpha \right]}_{D}^{20}$ + 9.0 (*c* 0.1, CHCl_3_); UV (MeOH) *λ*_max_ (log *ε*) 212 (4.13), 237 (3.60), 305 (3.93) nm; ECD (0.002 M, MeOH) *λ*_max_ (Δ*ε*) 207 (+1.69), 244 (−0.51), 304 (+1.06) nm; IR (KBr) *ν*_max_ 3443, 2926, 1719, 1635, 1372, 1267 cm^−1^; ^1^H and ^13^C NMR data, see Table 1; HRESIMS *m/z* 469.2188 [M + Na]^+^ (calcd for C_25_H_34_O_7_Na, 469.2197).

*Myrothecisin B (***2***)*: pale yellow oil; ${\left[\alpha \right]}_{D}^{20}$ + 70.1 (*c* 0.1, CHCl_3_); UV (MeOH) *λ*_max_ (log *ε*) 211 (4.10), 238 (3.62), 305 (3.88) nm; ECD (0.002 M, MeOH) *λ*_max_ (Δ*ε*) 207 (+1.62), 249 (−0.09), 305 (+0.49) nm; IR (KBr) *ν*_max_ 3424, 2940, 1733, 1638, 1460, 1371, 1263 cm^−1^; ^1^H and ^13^C NMR data, see Table 1; HRESIMS *m/z* 469.2200 [M + Na]^+^ (calcd for C_25_H_34_O_7_Na, 469.2197).

*Myrothecisin C (***3***)*: pale yellow oil; ${\left[\alpha \right]}_{D}^{20}$ + 63.5 (*c* 0.1, CHCl_3_); UV (MeOH) *λ*_max_ (log *ε*) 212 (4.09), 239 (3.65), 307 (3.81) nm; ECD (0.002 M, MeOH) *λ*_max_ (Δ*ε*) 219 (+2.29), 293 (−0.49), 327 (+0.22) nm; IR (KBr) *ν*_max_ 3480, 2936, 1732, 1612, 1373, 1254 cm^−1^; ^1^H and ^13^C NMR data, see Table 1; HRESIMS *m/z* 453.2238 [M + Na]^+^ (calcd for C_25_H_34_O_6_Na, 453.2248).

*Myrothecisin D (***4***)*: pale yellow oil; ${\left[\alpha \right]}_{D}^{20}$ + 20.0 (*c* 0.1, CHCl_3_); UV (MeOH) *λ*_max_ (log *ε*) 203 (4.16), 224 (3.56), 300 (3.86) nm; ECD (0.002 M, MeOH) *λ*_max_ (Δ*ε*) 220 (+1.57), 242 (+0.92), 295 (−0.27) nm; IR (KBr) *ν*_max_ 2928, 1718, 1621, 1370, 1264, 1027 cm^−1^; ^1^H and ^13^C NMR data, see Table 1; HRESIMS *m/z* 411.2139 [M + Na]^+^ (calcd for C_23_H_32_O_5_Na, 411.2142).

*Myrothelactone A* *(***5***)*: colorless crystal; melting point (mp) 174–175 °C; ${\left[\alpha \right]}_{D}^{20}$ − 36.0 (*c* 0.1, MeOH); UV (MeOH) *λ*_max_ (log *ε*) 246 (3.82), 328 (3.19) nm; ECD (0.004 *M*, MeOH) *λ*_max_ (Δ*ε*) 206.5 (−0.22), 241 (+0.24), 259 (−0.27) nm; IR (KBr) *ν*_max_ 3747, 3630, 3159, 2959, 1668, 1558, 1398, 1237 cm^−1^; ^1^H and ^13^C NMR data, see Table 2; HRESIMS *m/z* 251.0563 [M − H]^−^ (calcd for C_12_H_11_O_6_, 251.0561).

*Myrothelactone B (***6***)*: white powder; mp 169–171 °C; ${\left[\alpha \right]}_{D}^{20}$ − 30.0 (*c* 0.1, MeOH); UV (MeOH) *λ*_max_ (log *ε*) 245 (3.81), 328 (3.17) nm; ECD (0.004 M, MeOH) *λ*_max_ (Δ*ε*) 206.5 (−0.78), 241 (+0.24), 259 (−0.13) nm; IR (KBr) *ν*_max_ 3749, 2922, 1681, 1651, 1619, 1459, 1399 cm^−1^; ^1^H and ^13^C NMR data, see Table 2; HRESIMS *m/z* 265.0355 [M − H]^−^ (calcd for C_12_H_9_O_7_, 265.0354).

*Myrothelactone C (***7***)*: white powder; mp 160–161 °C; UV (MeOH) *λ*_max_ (log *ε*) 228 (3.91), 263 (3.43), 325 (3.16) nm; IR (KBr) *ν*_max_ 3750, 3675, 3615, 1736, 1651, 1558, 1540, 1399 cm^−1^; ^1^H and ^13^C NMR data, see Table 2; HRESIMS *m/z* 249.0408 [M − H]^−^ (calcd for C_12_H_9_O_6_, 249.0405).

*Myrothelactone D (***8***)*: white powder; mp 219–221 °C; UV (MeOH) *λ*_max_ (log *ε*) 231 (3.92), 263 (3.45), 325 (3.16) nm; IR (KBr) *ν*_max_ 3749, 3673, 3445, 3197, 1716, 1682, 1539, 1457, 1399 cm^−1^; ^1^H and ^13^C NMR data, see Table 2; HRESIMS *m/z* 249.0407 [M − H]^−^ (calcd for C_12_H_9_O_6_, 249.0405).

### 3.5. X-ray Structure Determination of Compound ***5***

Compound **5** was obtained as a colorless needles crystal with molecular formula C_12_H_12_O_6_. Orthorhombic, space group *P*2_1_2_1_2_1_, *a* = 4.9041(2) Å, *b* = 13.8470(5) Å, *c* = 15.7443(6) Å, *α* = 90.00°, *β* = 90.00°, *γ* = 90.00°, *V* = 1069.15(7) Å3, *Z* = 4, *D*_calcd_ = 1.567 Mg/m^3^, *μ* = 1.089 mm^−1^, *F*(000) = 528, crystal size 0.30 mm × 0.18 mm × 0.15 mm, *T* = 293(2) K. A total of 1478 unique reflections (2*θ* < 50°) were collected on a CCD area detector diffractometer with graphite monochromated Cu K*α* radiation (*λ* = 1.54178 Å). The structure was solved by direct methods (SHELXS-97) and expanded using Fourier techniques (SHELXL-97). The final cycle of full-matrix least squares refinement was based on 1478 unique reflections (2*θ* < 50°) and 165 variable parameters and converged with unweighted and weighted agreement factors of *R*_1_ = 0.0326, w*R*_2_ = 0.0885 and *R* = 0.0880 for *I* > 2sigma(*I*) data. Absolute structure parameter: −0.2(2). The deposited number of compound **5** in the Cambridge Crystallographic Data Centre is 980155.

### 3.6. α-Glucosidase Inhibitory Assays

The human-sourced α-glucosidase was recombinant expressed in the yeast *Saccharomyces cerevisiae* and the inhibitory effects of compounds **1**–**13** were tested using p-nitrophenyl-α-d-glucopyranoside (pNPG) as substrate [31,32,33]. The sample was dissolved in sodium phosphate buffer (PBS, pH 6.8) at three concentrations. 10 μL of the sample solution, 20 μL of 2.5 mM pNPG solution (in phosphate buffer) and 20 μL of PBS were mixed in a 96-well microplate and incubated at 37 °C for 5 min. A volume of 10 μL of α-glucosidase diluted to 0.2 U/mL by 0.01 M PBS was then added to each well. After incubating at 37 °C for 15 min, the absorbance at 405 nm was recorded by a Spectra max 190 micro plate reader (Molecular Devices Inc., San Jose, CA, USA). The blank was prepared by adding phosphate buffer instead of the α-glucosidase and the positive control was acarbose. Blank readings (no enzyme) were subtracted from each well and results were compared to the control. The inhibition (%) was calculated as [1 − (OD_drug_/OD_blank_)] × 100%. The IC_50_ value was calculated as the compound concentration that is required for 50% inhibition and the IC_50_ value of acarbose was 0.47 mM.

## 4. Conclusions

This study revealed eight new fungal NPs, meroterpenoids **1**–**4** and isocoumarinoids **5**–**8**, from the culture of the salt-tolerant plant-associated fungus *Myrothecium* sp. OUCMDZ-2784. The new compounds **1**–**5** and **7** exhibited α-glucosidase inhibitory activity. Combined with bioactive NPs from mangrove-derived fungi [34,35,36,37,38], the results indicated that fungi living in the salt-tolerant plants are an important biological resources for new and bioactive NPs.

## Figures and Tables

**Table 1:** ^1^H (600 MHz) and ^13^C (150 MHz) NMR data for **1**–**4** in DMSO-*d*_6_.

No.	1		2		3		4	
	δ_C_, Type	δ_H_, Mult. (*J* in Hz)	δ_C_, Type	δ_H_, Mult. (*J* in Hz)	δ_C_, Type	δ_H_, Mult. (*J* in Hz)	δ_C_, Type	δ_H_, Mult. (*J* in Hz)
1	35.1, CH_2_	1.16, m; 1.55 ^a^	38.4, CH_2_	1.17, m; 1.57 ^a^	114.7, CH	4.94, brs	116.3, CH	4.88, s
2	71.4, CH	4.83, ddd (11.5, 10.1, 4.1)	64.4, CH	3.65, m	28.0, CH_2_	1.89, m; 1.23, m	31.6, CH_2_	1.72, m
3	78.2, CH	2.96, dd (10.1, 4.8)	83.3, CH	4.29, d (9.9)	75.6, CH	4.62, dd (8.6, 6.5)	71.5, CH	3.30 ^a^
4	39.2, C		39.0, C		35.6, C		36.9, C	
5	45.6, CH	1.56 ^a^	45.5, CH	1.63 ^a^	43.1, CH	2.54, m	43.7, CH	2.45, m
6	20.7, CH_2_	1.54 ^a^; 1.47, m	20.4, CH_2_	1.56 ^a^; 1.48, m	26.7, CH_2_	1.79, m	26.7, CH_2_	1.77, m
7	30.6, CH_2_	1.55 ^a^; 1.35, m	30.5, CH_2_	1.57 ^a^; 1.38, m	30.2, CH_2_	1.54, m	30.3, CH_2_	1.52, m
8	36.0, CH	1.83, m	35.6, CH	1.85, m	43.3, CH	1.30, m	42.9, CH	1.29, m
9	98.8, C		98.8, C		43.7, C		43.7, C	
10	42.8, C		42.5, C		143.9, C		143.0, C	
11	30.4, CH_2_	3.03, d (16.1); 2.79, d (16.0)	30.2, CH_2_	3.11, d (16.5); 2.81, d (16.5)	23.5, CH_2_	2.68, d (12.9); 2.53, d (12.9)	23.3, CH_2_	2.63, d (12.7); 2.50, d (12.7)
12	15.2, CH_3_	0.64, d (6.3)	15.1, CH_3_	0.68, d (6.3)	17.1, CH_3_	1.03, d (6.6)	17.2, CH_3_	1.02, d (6.7)
13	28.7, CH_3_	0.97, s	28.4, CH_3_	0.79, s	25.0, CH_3_	0.88, s	25.2, CH_3_	0.95, s
14	17.0, CH_3_	0.78, s	17.5, CH_3_	0.81, s	17.1, CH_3_	0.73, s	14.8, CH_3_	0.59, s
15	16.6, CH_3_	1.04, s	16.6, CH_3_	1.03, s	23.0, CH_3_	0.96, s	22.9, CH_3_	0.91, s
16	170.3, C		170.3, C		169.8, C			
17	21.3, CH_3_	1.91, s	21.0, CH_3_	2.02, s	21.0, CH_3_	1.98, s		
1′	111.2, C		111.2, C		111.4, C		111.3, C	
2′	159.8, C		159.8, C		164.5, C		164.8, C	
3′	112.3, C		112.2, C		110.2, C		110.2, C	
4′	149.5, C		149.4, C		145.4, C		145.4, C	
5′	101.5, CH	6.56, s	101.6, CH	6.61, s	107.5, CH	6.51, s	107.3, CH	6.50, s
6′	167.3, C		167.3, C		164.6, C		164.6, C	
7′	193.8, CH	10.06, s	193.8, CH	10.07, s	193.3, CH	9.96, s	193.4, CH	9.96, s
8′	60.5, CH_2_	4.74, s	60.4, CH_2_	4.74, s	59.9, CH_2_	4.70, s	59.8, CH_2_	4.70, s
2/3-OH		4.94, d (4.7)		4.62, d (4.9)				4.13, s
2′-OH		12.16, s		12.24, s		12.84, s		12.78, s
6′-OH								10.55, brs
8′-OH		5.44, s		5.39, s		5.35, s		5.36, s

^a^ Overlapped.

**Table 2:** ^1^H and ^13^C NMR data for **5**–**8** in DMSO-*d*_6_.

No.	5 ^a^		6 ^b^		7 ^b^		8 ^b^	
	δ_C_, Type	δ_H_, mult. (*J* in Hz)	δ_C_, Type	δ_H_, mult. (*J* in Hz)	δ_C_, Type	δ_H_, mult. (*J* in Hz)	δ_C_, Type	δ_H_, mult. (*J* in Hz)
1	165.4, C		164.9, C		163.4, C		165.2, C	
3	143.1, CH	7.45, s	144.4, CH	7.62, s	153.1, CH	8.47, s	151.0, C	
4	118.6, C		117.7, C		114.7, C		118.7, C	
5	100.5, CH	6.78, d (2.2)	101.6, CH	6.84, s	102.3, CH	7.46, s	101.6, CH	6.86, s
6	166.4, C		166.4, C		166.8, C		166.3, C	
7	100.4, CH	6.63 (d, 2.2)	100.6, CH	6.63, s	101.2, CH	6.68, s	99.8, CH	6.48, s
8	163.4, C		163.3, C		163.2, C		162.6, C	
9	99.9, C		99.9, C		99.9, C		99.2, C	
10	137.6, C		136.8, C		134.8, C		138.6, C	
11	68.8, CH	4.66, td (5.2, 5.2)	68.5, CH	5.01, s	198.3, C		173.9, C	
12	64.8, CH_2_	3.62, ddd (11.3, 5.3, 5.3); 3.51, ddd (11.3, 5.3, 5.3)	173.4, C		65.9, CH_2_	4.57, d (4.7)	17.9, CH_3_	2.29, s
6-OCH_3_	56.0, CH_3_	3.88, s	56.0, CH_3_	3.84, s	56.0, CH_3_	3.87, s	55.8, CH_3_	3.80, s
8-OH		11.37, s		11.22, s		11.03, s		11.21, s
11-OH		5.50, d (4.7)						
12-OH		4.81, t (5.2)				5.28, t (5.0)		

^a^ Data were measured at 600 MHz (^1^H) and 150 MHz (^13^C). ^b^ Data were measured at 500 MHz (^1^H) and 125 MHz (^13^C).

## Supplementary Materials

The following are available online at http://www.mdpi.com/1660-3397/16/10/363/s1, Figures S1–S4: DFT-optimized structures for low-energy conformers of compounds **1**, **3**, **5** and **6**, Figures S5–S11: NMR spectra of compound **1** in DMSO-*d*_6_, Figures S12–S18: NMR spectra of compound **2** in DMSO-*d*_6_, Figures S19–S25: NMR spectra of compound **3** in DMSO-*d*_6_, Figures S26–S32: NMR spectra of compound **4** in DMSO-*d*_6_, Figures S33–S38: NMR spectra of compound **5** in DMSO-*d*_6_, Figures S39–S44: NMR spectra of compound **6** in DMSO-*d*_6_, Figures S45–S50: NMR spectra of compound **7** in DMSO-*d*_6_, Figures S51–S56: NMR spectra of compound **8** in DMSO-*d*_6_, Figure S57: ^1^H- and ^13^C-NMR spectra of compounds **9**–**13** in DMSO-*d*_6_, Figure S58: HRESI-MS spectra of compounds **1**–**8**, Figure S59: ESI-MS spectra of compounds **9**–**13**, Table S1: ^1^H and ^13^C NMR data for compounds **9**–**13** in DMSO-*d*_6_.
