# Supplementary material for: Meroterpenoids and Isocoumarinoids from a Myrothecium Fungus Associated with Apocynum venetum
Source: Mar Drugs. 2018 Oct 1;16(10):363. doi: 10.3390/md16100363 (PMC6213923; doi:10.3390/md16100363)
Supplement: Supplementary file 1 [file marinedrugs-16-00363-s001.pdf]

Supplementary Material

Meroterpenoids and Isocoumarinoids from a *Myrothecium* Fungus Associated with *Apocynum venetum*

Yanchao Xu [1,2,3,†], Cong Wang [2,4,†], Haishan Liu [2], Guoliang Zhu [2], Peng Fu [2,3], Liping Wang [1,2,*] and Weiming Zhu [1,2,3,*]

1 State Key Laboratory of Functions and Applications of Medicinal Plants, Guizhou Medical University, Guiyang 550014, China; m18586818694@163.com
2 Key Laboratory of Marine Drugs, Ministry of Education of China, School of Medicine and Pharmacy, Ocean University of China, Qingdao 266003, China; wangcong123206@163.com(C.W.); liuhaishan_229@outlook.com (H.L.); guoliangzhu2015@hotmail.com (G.Z.); fupeng@ouc.edu.cn (P.F.)
3 Laboratory for Marine Drugs and Bioproducts, Qingdao National Laboratory for Marine Science and Technology; Qingdao 266003, China
4 Guangxi Key Laboratory of Chemistry and Engineering of Forest Products, School of Chemistry and Chemical Engineering, Guangxi University for Nationalities, Nanning 530006, China
* Correspondence: lipingw2006@163.com (L.W.); weimingzhu@ouc.edu.cn (W.Z.); Tel./Fax: +86-532-8203-1268 (W.Z.)
† These authors contributed equally to this paper.

List of Supplementary Material

Theory and Calculation Details......S3
Figure S1. DFT-optimized structures for low-energy conformers of compound **1**......S4
Figure S2. DFT-optimized structures for low-energy conformers of compound **3**......S4
Figure S3. DFT-optimized structures for low-energy conformers of compound **5**......S4
Figure S4. DFT-optimized structures for low-energy conformers of compound **6**......S4
Figure S5. ^{1}H-NMR spectrum of myrothecisin A (**1**) in DMSO-d_6......S5
Figure S6. ^{13}C-NMR spectrum of myrothecisin A (**1**) in DMSO-d_6......S5
Figure S7. DEPT spectrum of myrothecisin A (**1**) in DMSO-d_6......S6
Figure S8. HSQC spectrum of myrothecisin A (**1**) in DMSO-d_6......S6
Figure S9. ^{1}H-^{1}H COSY spectrum of myrothecisin A (**1**) in DMSO-d_6......S7
Figure S10. HMBC spectrum of myrothecisin A (**1**) in DMSO-d_6......S7
Figure S11. NOESY spectrum of myrothecisin A (**1**) in DMSO-d_6......S8
Figure S12. ^{1}H-NMR spectrum of myrothecisin B (**2**) in DMSO-d_6......S8
Figure S13. ^{13}C-NMR spectrum of myrothecisin B (**2**) in DMSO-d_6......S9
Figure S14. DEPT spectrum of myrothecisin B (**2**) in DMSO-d_6......S9
Figure S15. HSQC spectrum of myrothecisin B (**2**) in DMSO-d_6......S10
Figure S16. ^{1}H-^{1}H COSY spectrum of myrothecisin B (**2**) in DMSO-d_6......S10
Figure S17. HMBC spectrum of myrothecisin B (**2**) in DMSO-d_6......S11

Figure S18. NOESY spectrum of myrothecisin B (**2**) in DMSO-d_6……S11
Figure S19. ^{1}H-NMR spectrum of myrothecisin C (**3**) in DMSO-d_6……S12
Figure S20. ^{13}C-NMR spectrum of myrothecisin C (**3**) in DMSO-d_6……S12
Figure S21. DEPT spectrum of myrothecisin C (**3**) in DMSO-d_6……S13
Figure S22. HSQC spectrum of myrothecisin C (**3**) in DMSO-d_6……S13
Figure S23. ^{1}H-^{1}H COSY spectrum of myrothecisin C (**3**) in DMSO-d_6……S14
Figure S24. HMBC spectrum of myrothecisin C (**3**) in DMSO-d_6……S14
Figure S25. NOESY spectrum of myrothecisin C (**3**) in DMSO-d_6……S15
Figure S26. ^{1}H-NMR spectrum of myrothecisin D (**4**) in DMSO-d_6……S15
Figure S27. ^{13}C-NMR spectrum of myrothecisin D (**4**) in DMSO-d_6……S16
Figure S28. DEPT spectrum of myrothecisin D (**4**) in DMSO-d_6……S16
Figure S29. HSQC spectrum of myrothecisin D (**4**) in DMSO-d_6……S17
Figure S30. ^{1}H-^{1}H COSY spectrum of myrothecisin D (**4**) in DMSO-d_6……S17
Figure S31. HMBC spectrum of myrothecisin D (**4**) in DMSO-d_6……S18
Figure S32. NOESY spectrum of myrothecisin D (**4**) in DMSO-d_6……S18
Figure S33. ^{1}H-NMR spectrum of myrothelactone A (**5**) in DMSO-d_6……S19
Figure S34. ^{13}C-NMR spectrum of myrothelactone A (**5**) in DMSO-d_6……S19
Figure S35. DEPT spectrum of myrothelactone A (**5**) in DMSO-d_6……S20
Figure S36. HSQC spectrum of myrothelactone A (**5**) in DMSO-d_6……S20
Figure S37. ^{1}H-^{1}H COSY spectrum of myrothelactone A (**5**) in DMSO-d_6……S21
Figure S38. HMBC spectrum of myrothelactone A (**5**) in DMSO-d_6……S21
Figure S39. ^{1}H-NMR spectrum of myrothelactone B (**6**) in DMSO-d_6……S22
Figure S40. ^{13}C-NMR spectrum of myrothelactone B (**6**) in DMSO-d_6……S22
Figure S41. DEPT spectrum of myrothelactone B (**6**) in DMSO-d_6……S23
Figure S42. HSQC spectrum of myrothelactone B (**6**) in DMSO-d_6……S23
Figure S43. ^{1}H-^{1}H COSY spectrum of myrothelactone B (**6**) in DMSO-d_6……S24
Figure S44. HMBC spectrum of myrothelactone B (**6**) in DMSO-d_6……S24
Figure S45. ^{1}H-NMR spectrum of myrothelactone C (**7**) in DMSO-d_6……S25
Figure S46. ^{13}C-NMR spectrum of myrothelactone C (**7**) in DMSO-d_6……S25
Figure S47. DEPT spectrum of myrothelactone C (**7**) in DMSO-d_6……S26
Figure S48. HSQC spectrum of myrothelactone C (**7**) in DMSO-d_6……S26
Figure S49. ^{1}H-^{1}H COSY spectrum of myrothelactone C (**7**) in DMSO-d_6……S27
Figure S50. HMBC spectrum of myrothelactone C (**7**) in DMSO-d_6……S27
Figure S51. ^{1}H-NMR spectrum of myrothelactone D (**8**) in DMSO-d_6……S28
Figure S52. ^{13}C-NMR spectrum of myrothelactone D (**8**) in DMSO-d_6……S28
Figure S53. DEPT spectrum of myrothelactone D (**8**) in DMSO-d_6……S29

Figure S54. HSQC spectrum of myrothelactone D (**8**) in DMSO-d_6……………………………………S29
Figure S55. ^{1}H-^{1}H COSY spectrum of myrothelactone D (**8**) in DMSO-d_6……………………………S30
Figure S56. HMBC spectrum of myrothelactone D (**8**) in DMSO-d_6……………………………………S30
Figure S57. ^{1}H and ^{13}C NMR spectra of the known compounds **9–13** in DMSO-d_6……………………………S31
Figure S58. HRESIMS spectra of the new compounds **1–8**…………………………………………………S36
Figure S59. ESIMS spectra of the known compounds **9–13**………………………………………………S40
Table S1. ^{1}H and ^{13}C NMR data for the known compounds **9–13** in DMSO-d_6. ………………………………S43

Theory and Calculation Details. The calculations were performed by using the density functional theory (DFT) as carried out in the Gaussian 03.[S1] The preliminary conformational distributions search was performed by HyperChem 7.5 software. All ground-state geometries were optimized at the B3LYP/6-31G(d) level. Conformers within a 2 kcal/mol energy threshold from the global minimum were selected to calculate the electronic transitions.[S2] The overall theoretical ECD spectra were obtained according to the Boltzmann weighting of each conformers. Solvent effects of methanol solution were evaluated at the same DFT level by using the SCRF/PCM method.[S3]

References:

(S1) Gaussian 03, Revision E.01, M. J. Frisch, G. W. Trucks, H. B. Schlegel, G. E. Scuseria, M. A. Robb, J. R. Cheeseman, J. A. Montgomery, Jr., T. Vreven, K. N. Kudin, J. C. Burant, J. M. Millam, S. S. Iyengar, J. Tomasi, V. Barone, B. Mennucci, M. Cossi, G. Scalmani, N. Rega, G. A. Petersson, H. Nakatsuji, M. Hada, M. Ehara, K. Toyota, R. Fukuda, J. Hasegawa, M. Ishida, T. Nakajima, Y. Honda, O. Kitao, H. Nakai, M. Klene, X. Li, J. E. Knox, H. P. Hratchian, J. B. Cross, V. Bakken, C. Adamo, J. Jaramillo, R. Gomperts, R. E. Stratmann, O. Yazyev, A. J. Austin, R. Cammi, C. Pomelli, J. W. Ochterski, P. Y. Ayala, K. Morokuma, G. A. Voth, P. Salvador, J. J. Dannenberg, V. G. Zakrzewski, S. Dapprich, A. D. Daniels, M. C. Strain, O. Farkas, D. K. Malick, A. D. Rabuck, K. Raghavachari, J. B. Foresman, J. V. Ortiz, Q. Cui, A. G. Baboul, S. Clifford, J. Cioslowski, B. B. Stefanov, G. Liu, A. Liashenko, P. Piskorz, I. Komaromi, R. L. Martin, D. J. Fox, T. Keith, M. A. Al-Laham, C. Y. Peng, A. Nanayakkara, M. Challacombe, P. M. W. Gill, B. Johnson, W. Chen, M. W. Wong, C. Gonzalez, and J. A. Pople, Gaussian, Inc., Wallingford CT, 2004.
(S2) (a) Casida, M. E. In Recent Advances in Density Functional Methods, part I; Chong, D. P., Eds.; World Scientific: Singapore, 1995; pp 155−192. (b) Gross, E. K. U.; Dobson, J. F.; Petersilka, M. Top. *Curr. Chem.* **1996**, *181*, 81−172. (c) Gross, E. K. U.; Kohn, W. *Adv. Quantum Chem.* **1990**, *21*, 255−291. (d) Runge, E.; Gross, E. K. U. *Phys. Rev. Lett.* **1984**, *52*, 997−1000.
(S3) (a) Miertus, S.; Tomasi, J. *Chem. Phys.* **1982**, *65*, 239−245. (b) Tomasi, J.; Persico, M. *Chem. Rev.* **1994**, *94*, 2027−2094. (c) Cammi, R.; Tomasi, J. *J. Comp. Chem.* **1995**, *16*, 1449−1458.

Figure S1. DFT-optimized structures for low-energy conformers of compound **1** at B3LYP/6-31G(d) level in methanol (PCM).

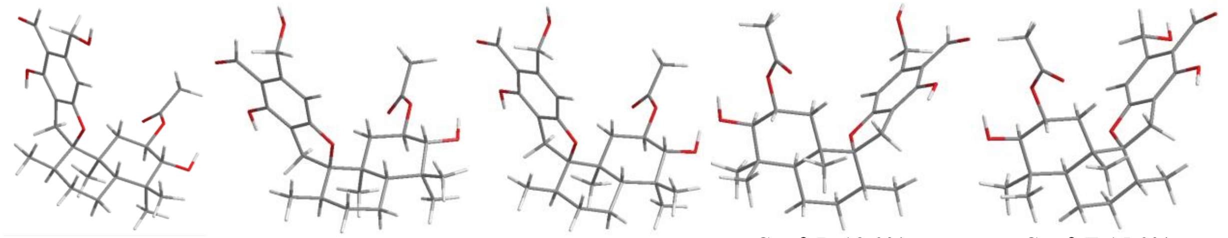

Conf.A 28.0% Conf. B 19.0% Conf. C 19.0% Conf. D 19.0% Conf. E 15.0%

Figure S2. DFT-optimized structures for low-energy conformers of compound **3** at B3LYP/6-31G(d) level in methanol (PCM).

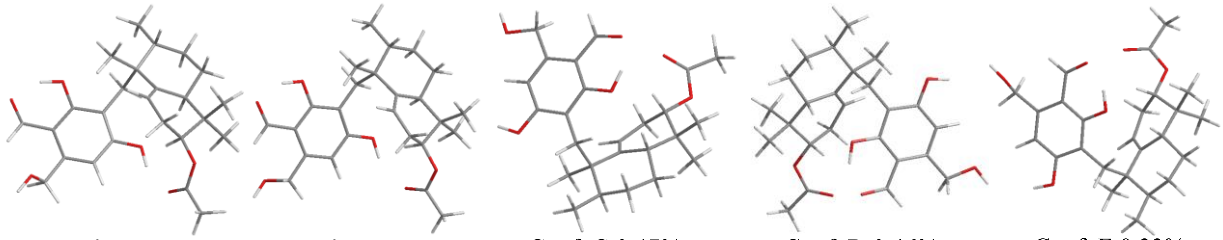

Conf. A 54.3% Conf. B 44.4% Conf. C 0.47% Conf. D 0.46% Conf. E 0.33%

Figure S3. DFT-optimized structures for low-energy conformers of compound **5** at B3LYP/6-31G(d) level in methanol (PCM).

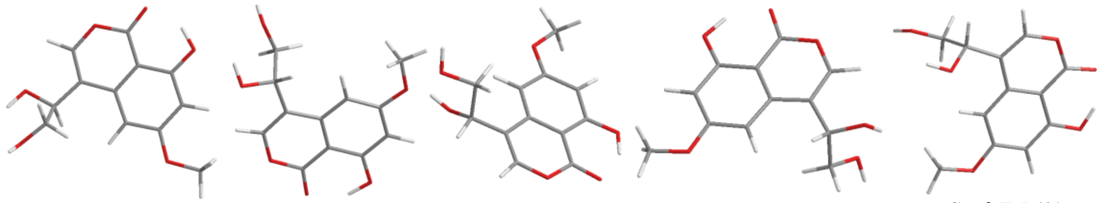

Conf. A 63.4% Conf. B 16.7% Conf. C 8.9% Conf. D 5.5% Conf. E 5.4%

Figure S4. DFT-optimized structures for low-energy conformers of compound **6** at B3LYP/6-31G(d) level in methanol (PCM).

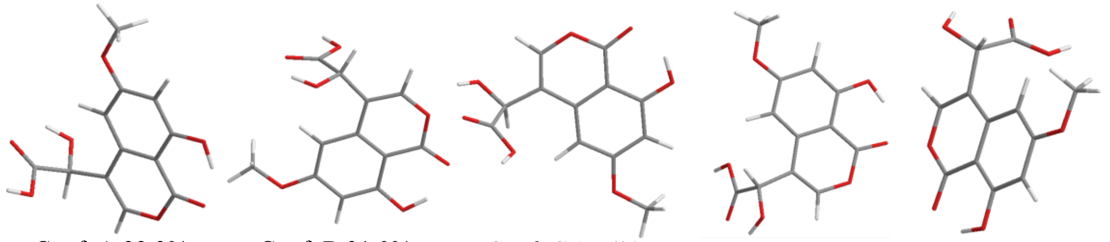

Conf. A 33.3% Conf. B 31.0% Conf. C 15.6% Conf. D 15.6% Conf. E 4.6%

Figure S5. ^{1}H-NMR spectrum of myrothecisin A (**1**) in DMSO-d_6

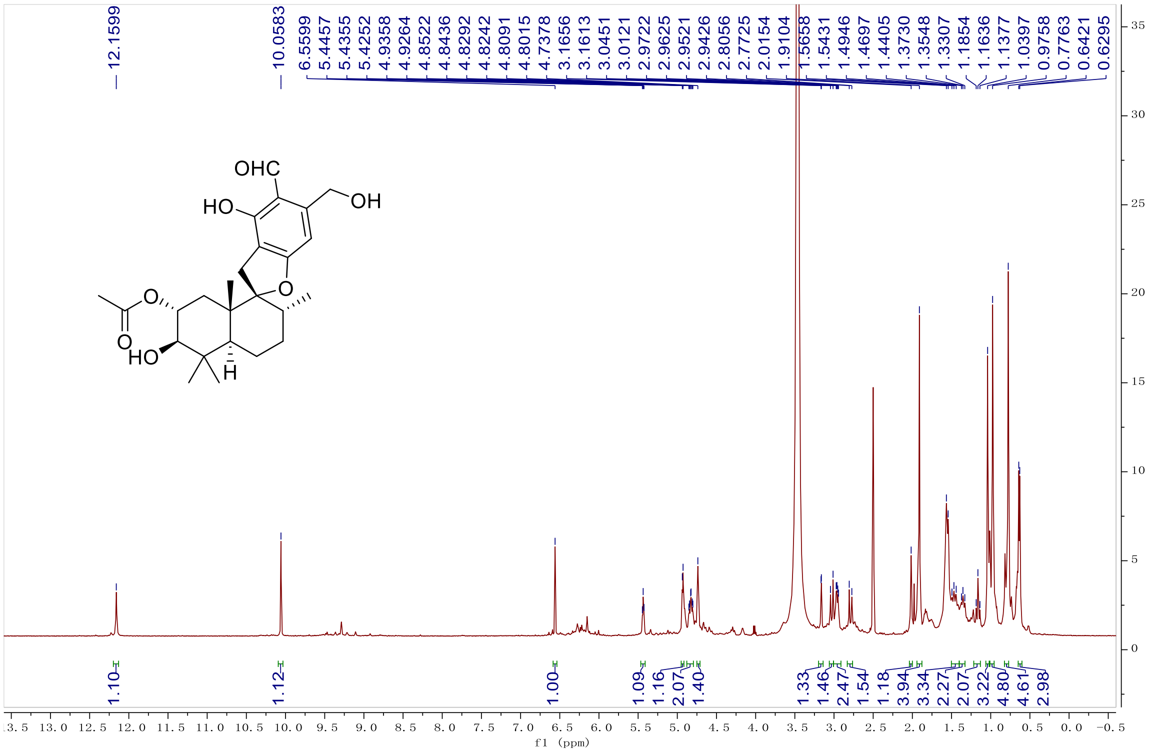

Figure S6. ^{13}C-NMR spectrum of myrothecisin A (**1**) in DMSO-d_6

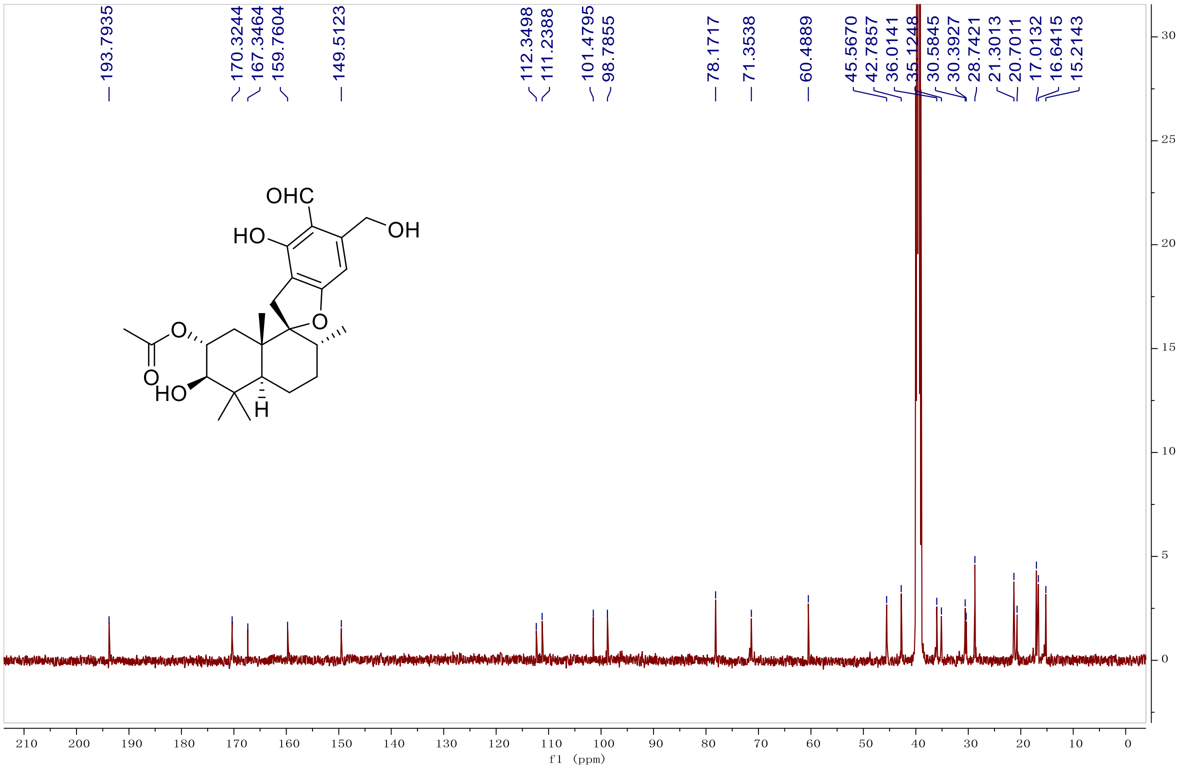

Figure S7. DEPT spectrum of myrothecisin A (**1**) in DMSO-*d*$_6$

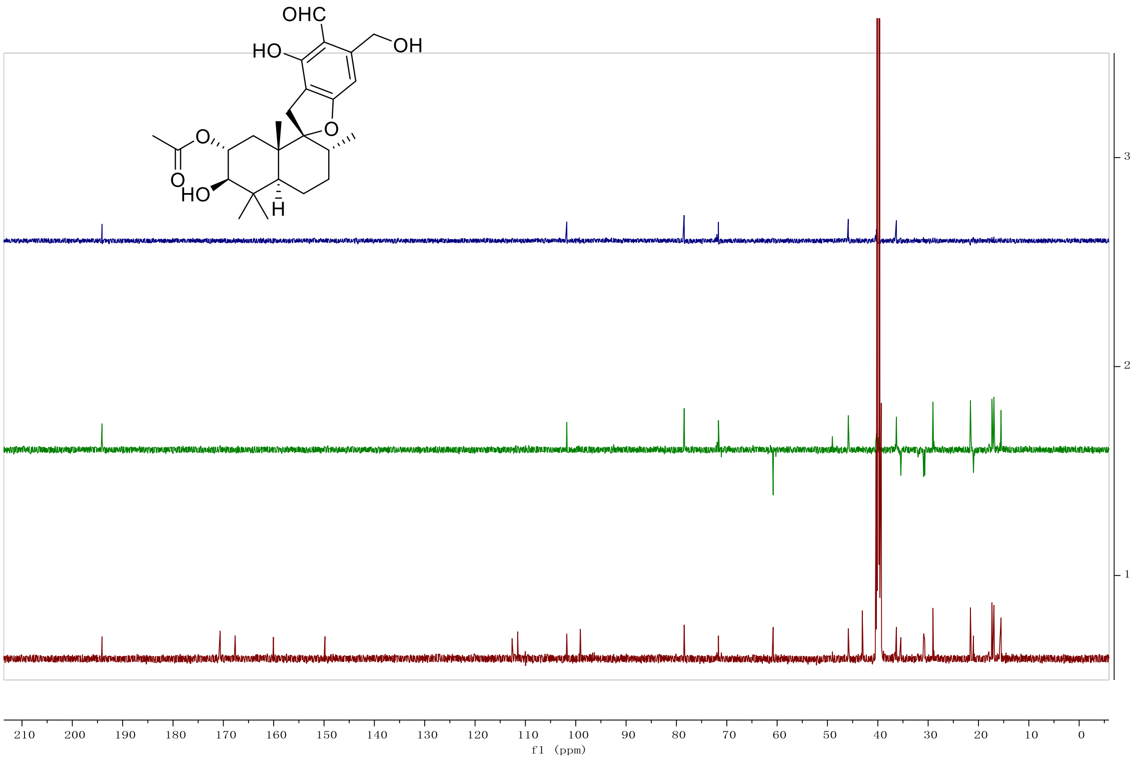

Figure S8. HSQC spectrum of myrothecisin A (**1**) in DMSO-*d*$_6$

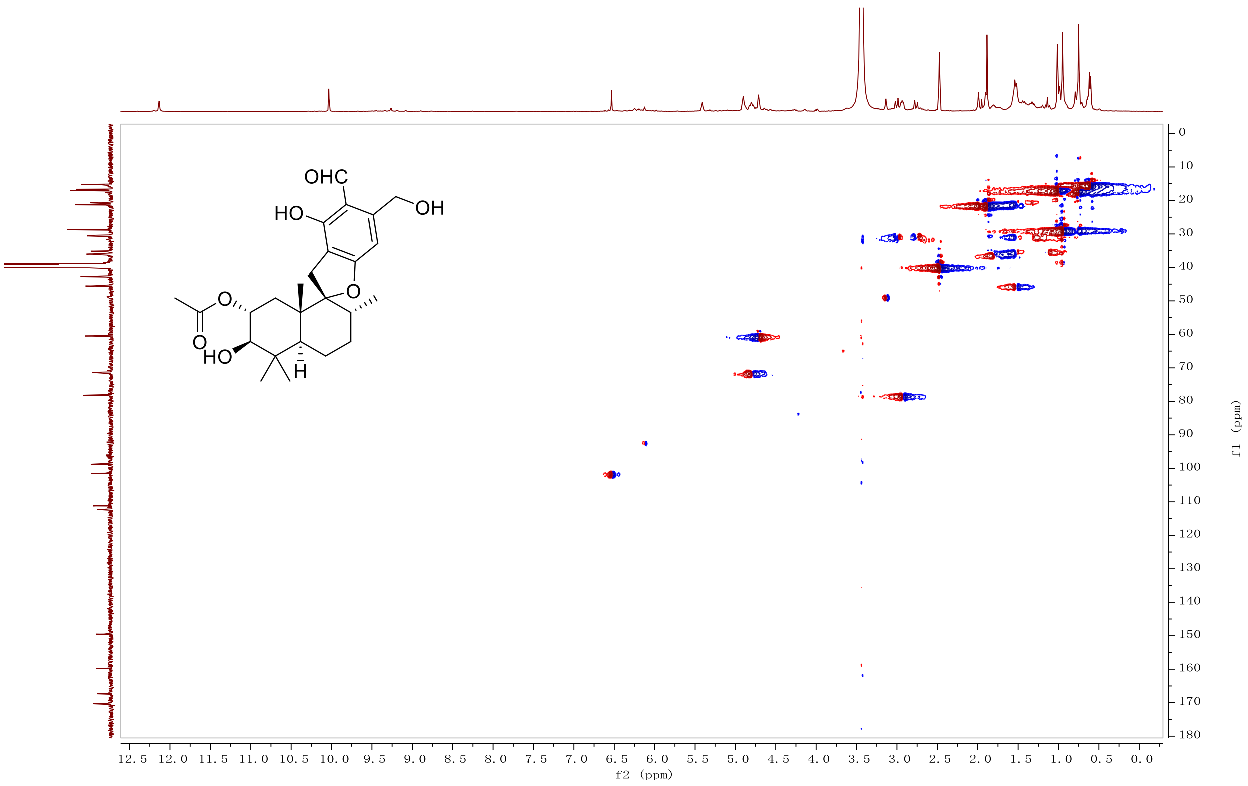

Figure S9. ¹H-¹H COSY spectrum of myrothecisin A (**1**) in DMSO-d_6

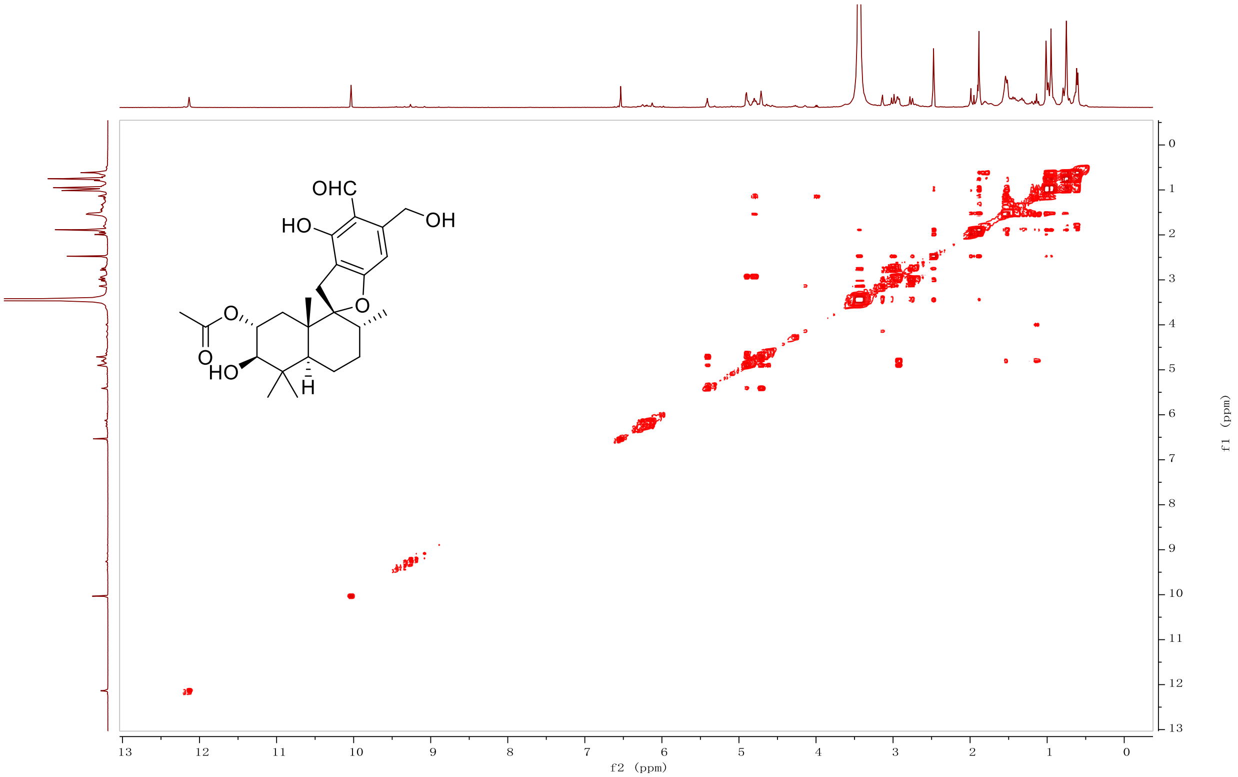

Figure S10. HMBC spectrum of myrothecisin A (**1**) in DMSO-d_6

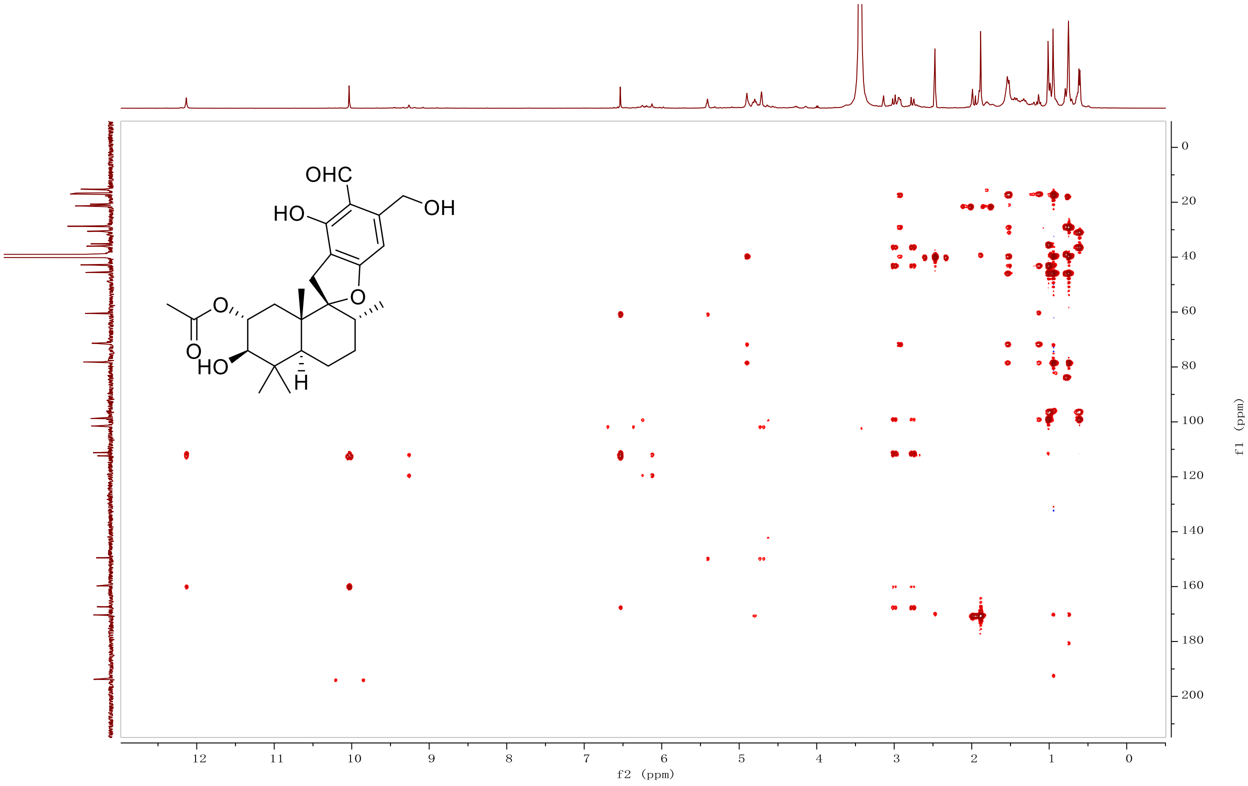

Figure S11. NOESY spectrum of myrothecisin A (**1**) in DMSO-d_6

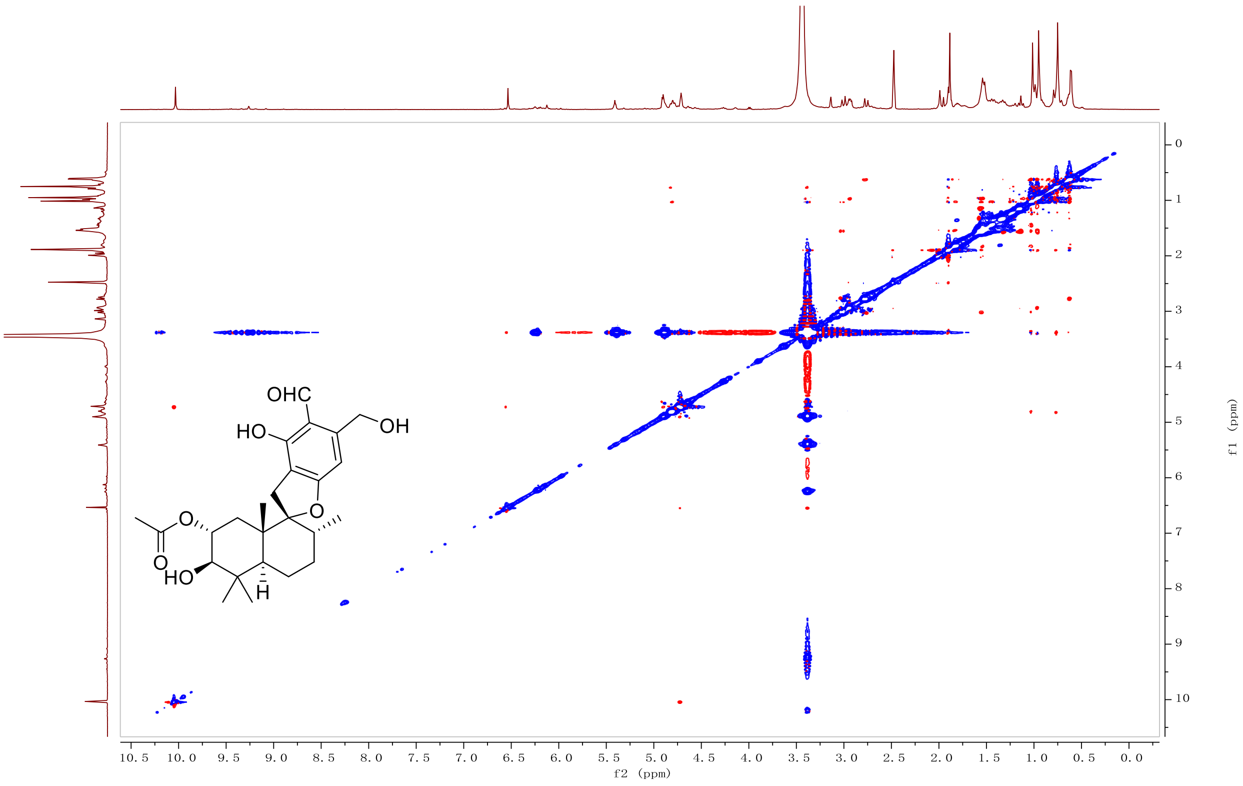

Figure S12. ^{1}H-NMR spectrum of myrothecisin B (**2**) in DMSO-d_6

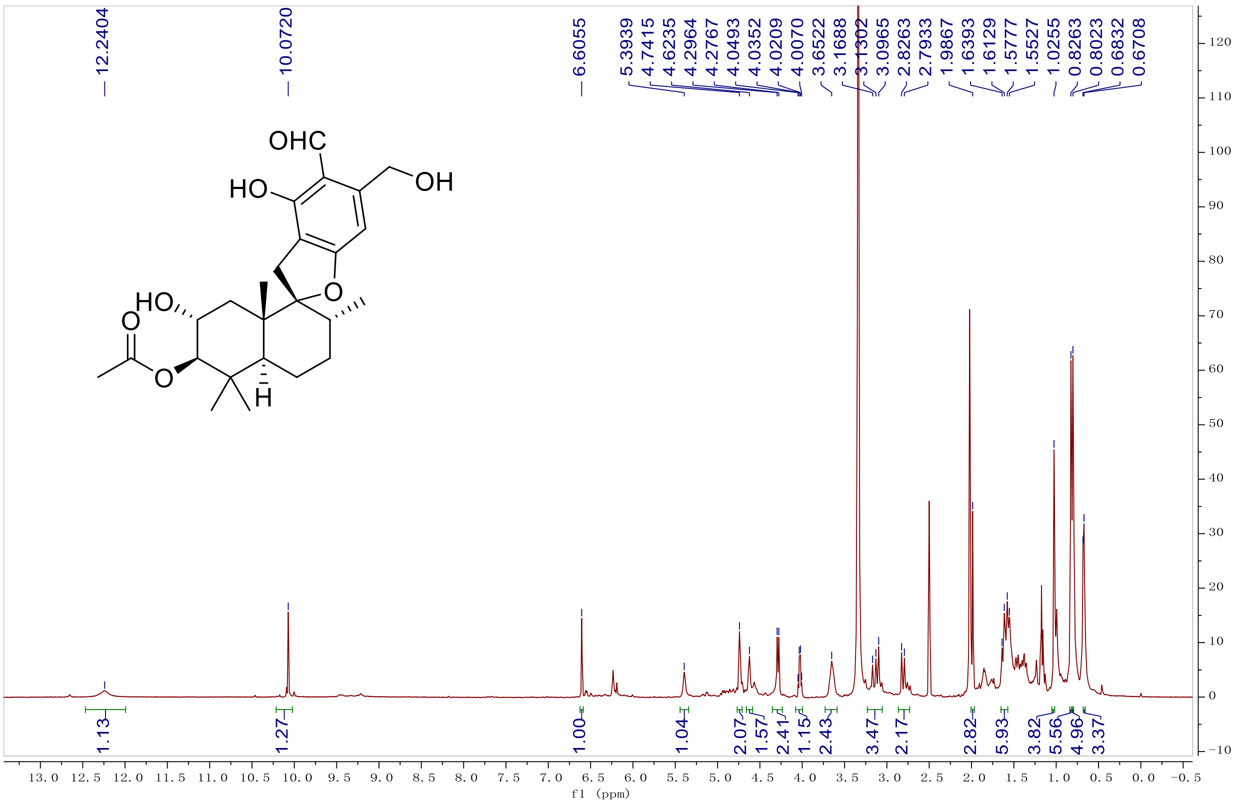

Figure S13. ^{13}C-NMR spectrum of myrothecisin B (**2**) in DMSO-d_6

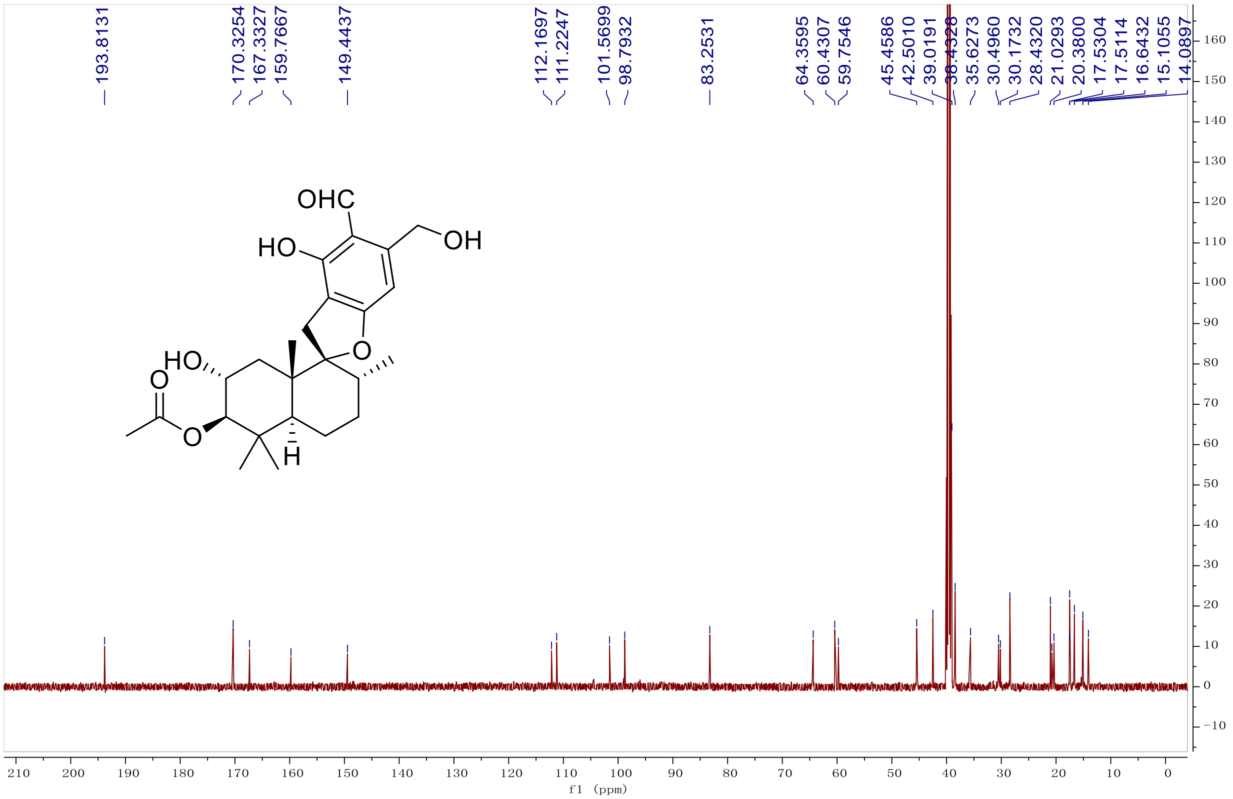

Figure S14. DEPT spectrum of myrothecisin B (**2**) in DMSO-d_6

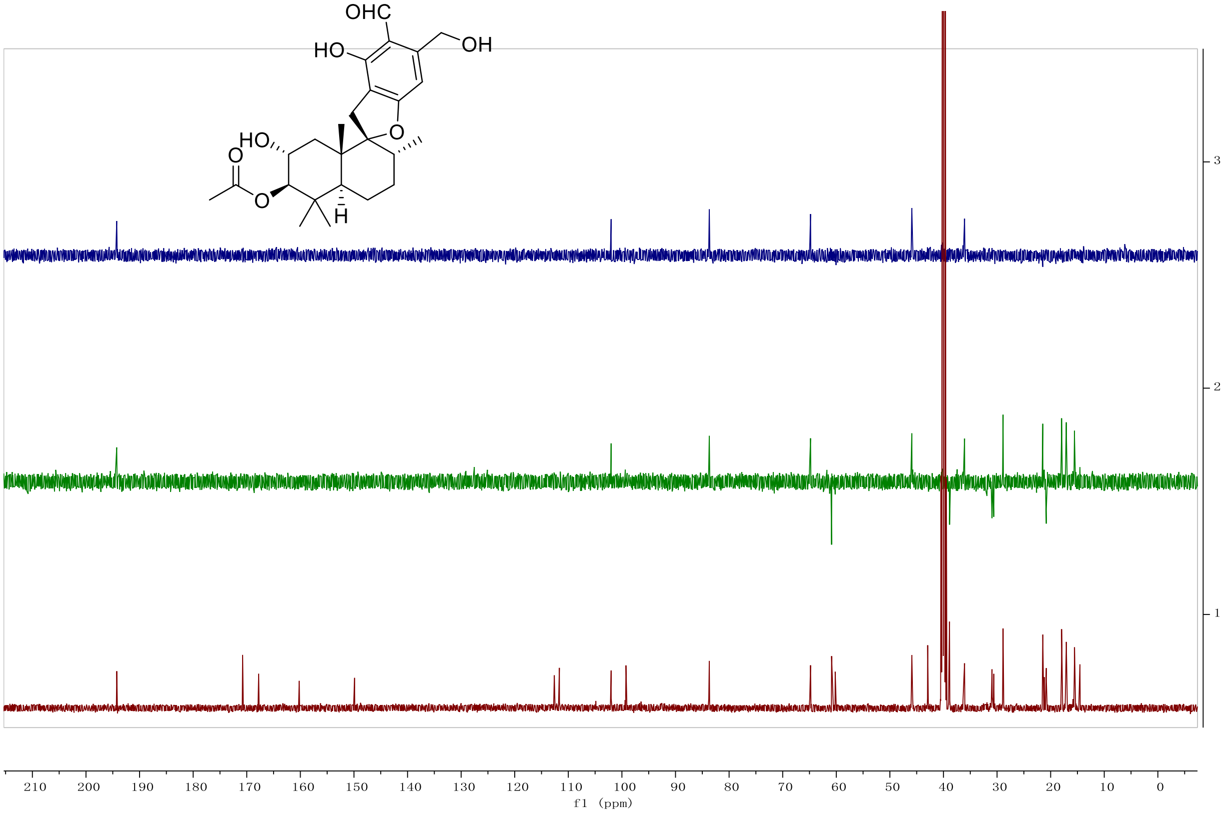

Figure S15. HSQC spectrum of myrothecisin B (**2**) in DMSO-d_6

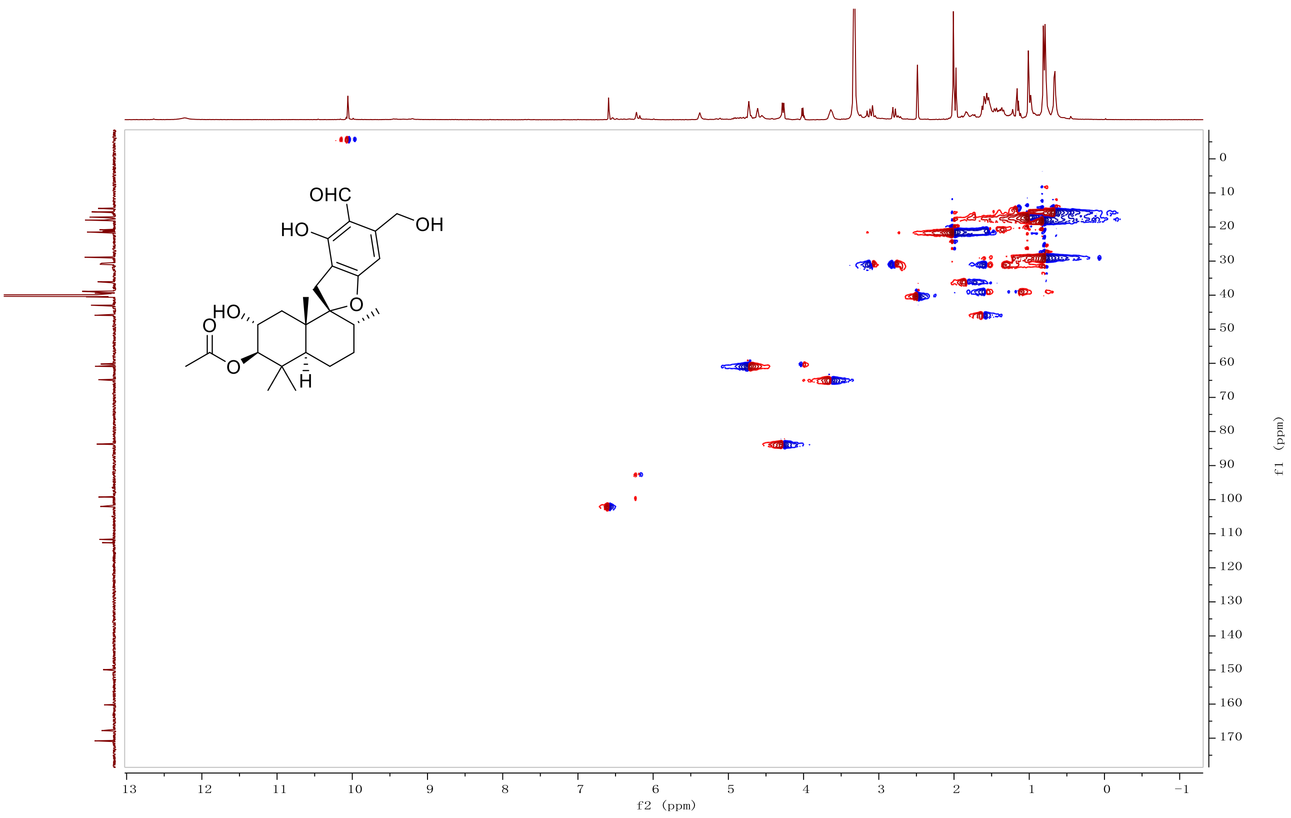

Figure S16. ^{1}H-^{1}H COSY spectrum of myrothecisin B (**2**) in DMSO-d_6

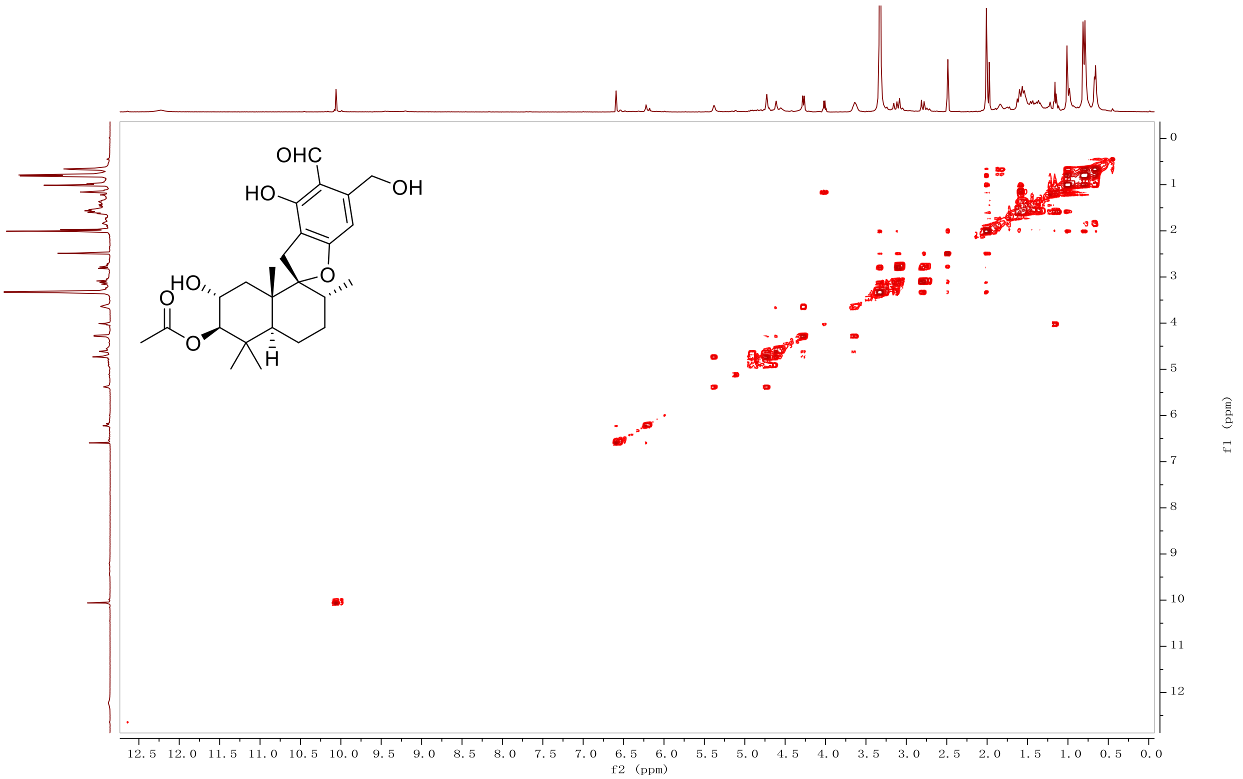

Figure S17. HMBC spectrum of myrothecisin B (**2**) in DMSO-d_6

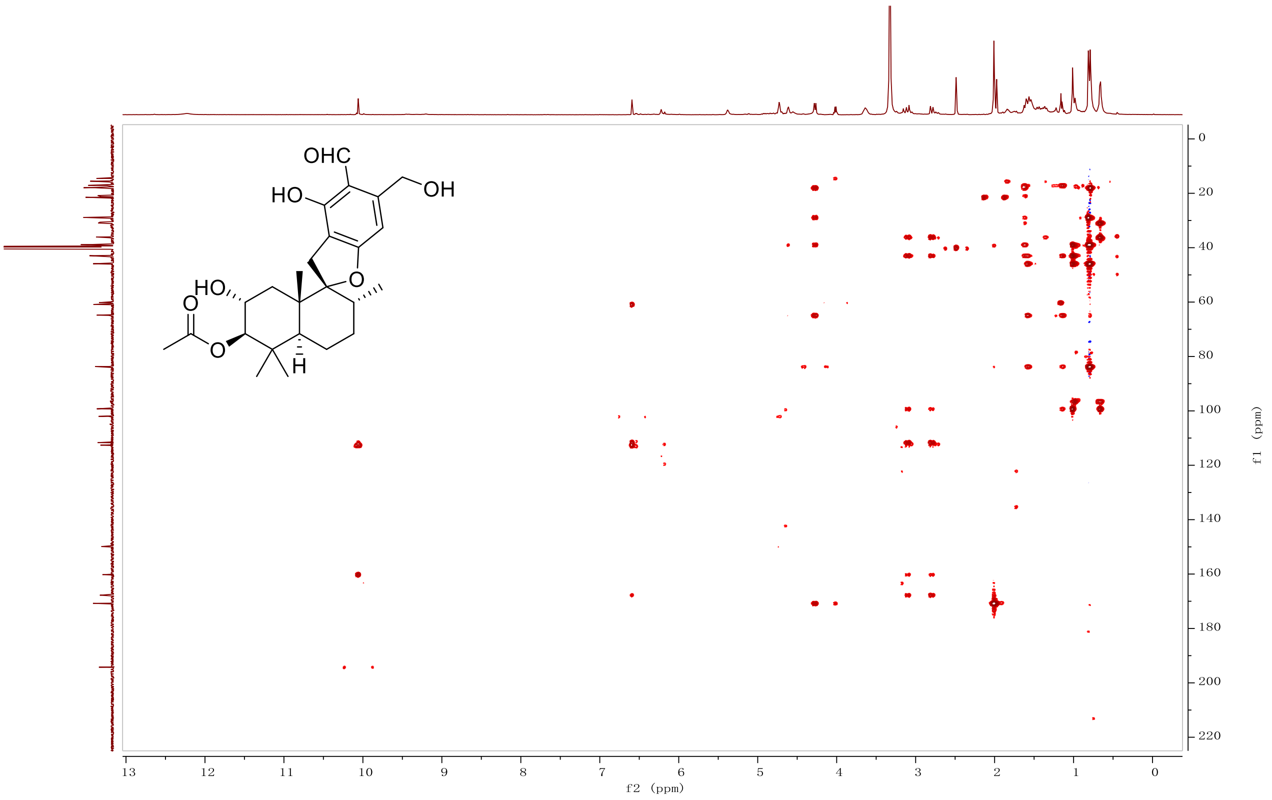

Figure S18. NOESY spectrum of myrothecisin B (**2**) in DMSO-d_6

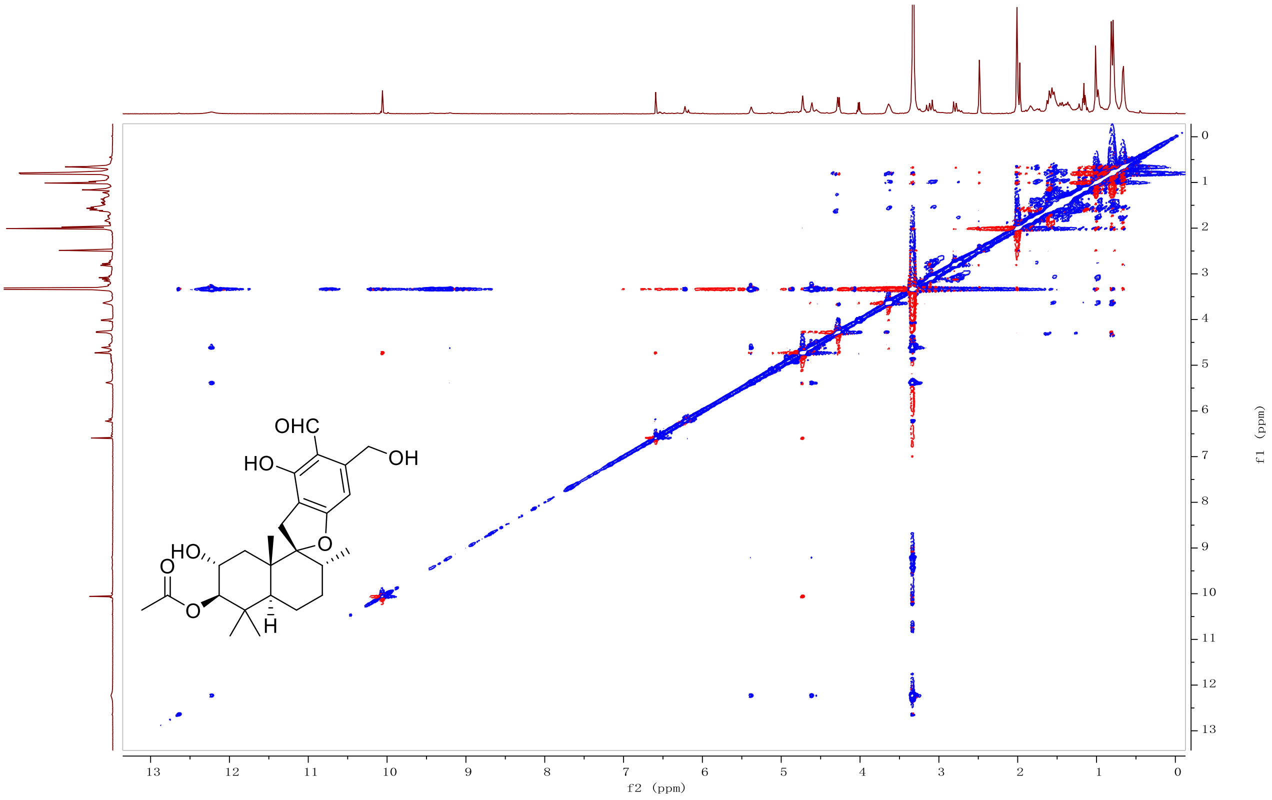

Figure S19. ^{1}H-NMR spectrum of myrothecisin C (**3**) in DMSO-d_6

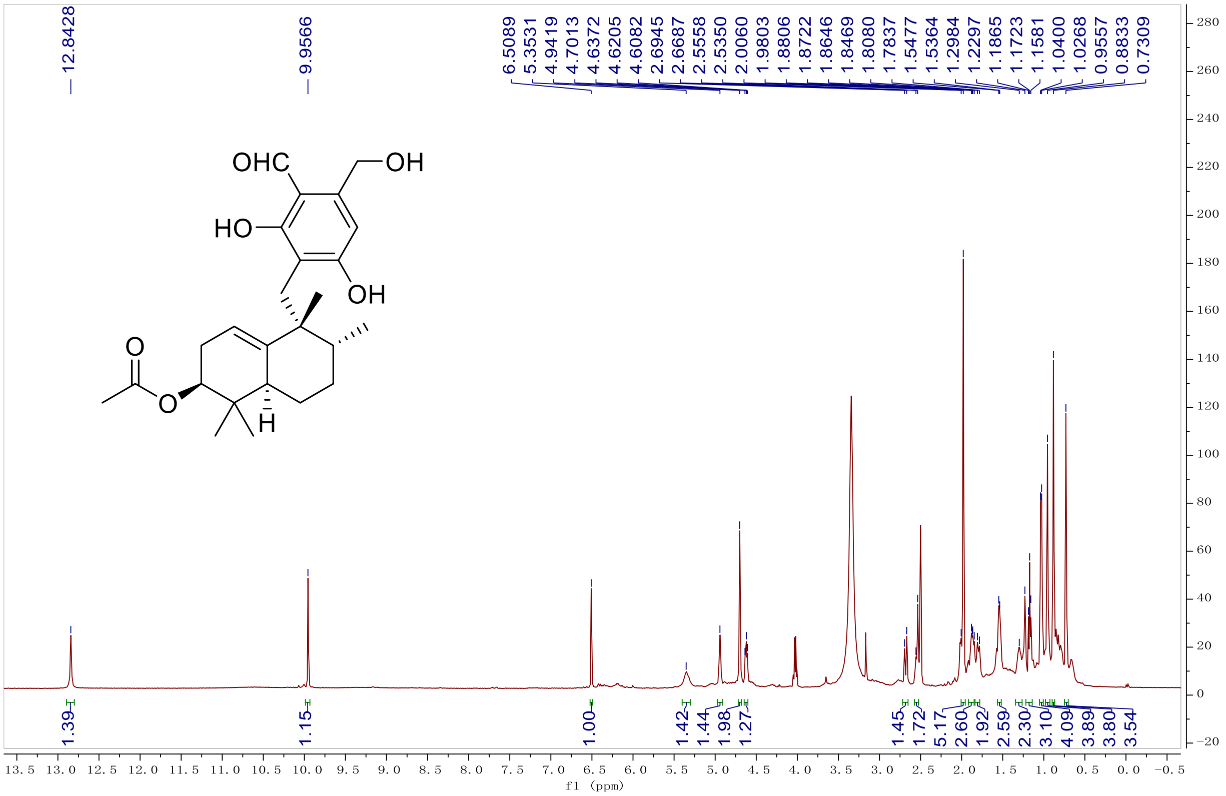

Figure S20. ^{13}C-NMR spectrum of myrothecisin C (**3**) in DMSO-d_6

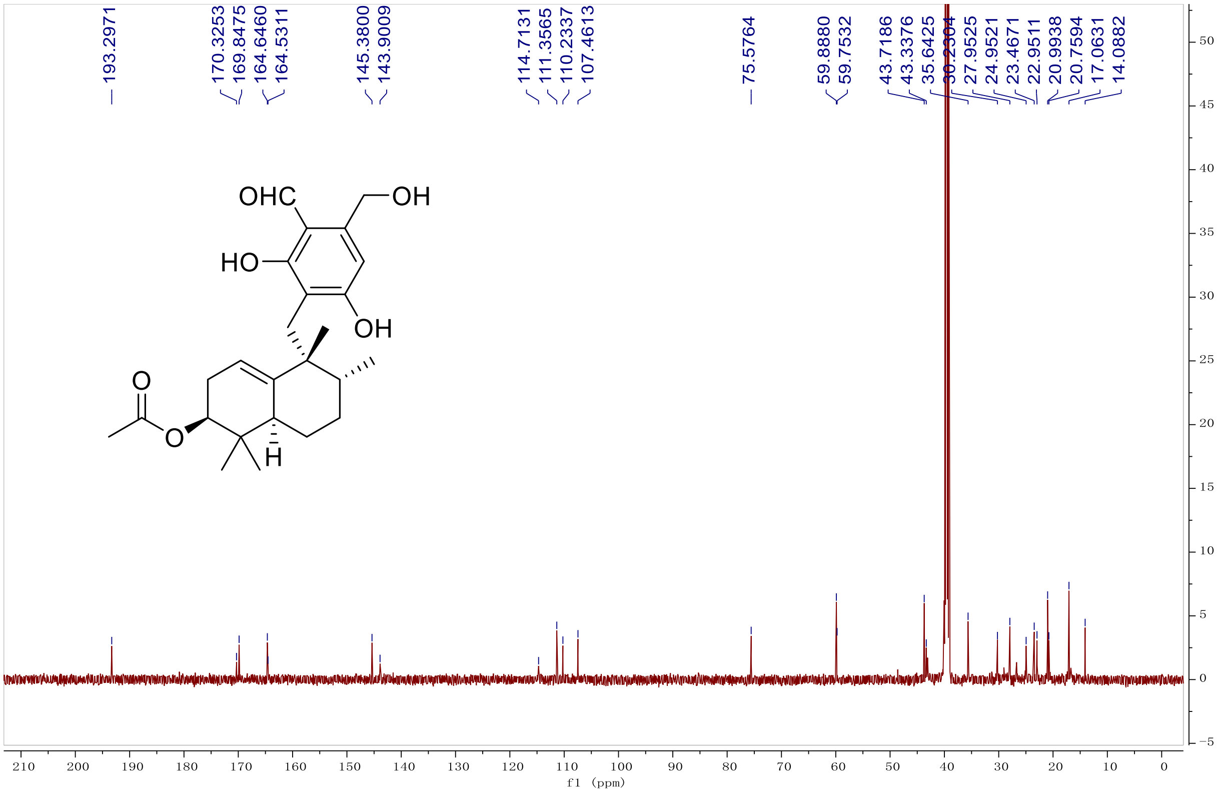

Figure S21. DEPT spectrum of myrothecisin C (**3**) in DMSO-d_6

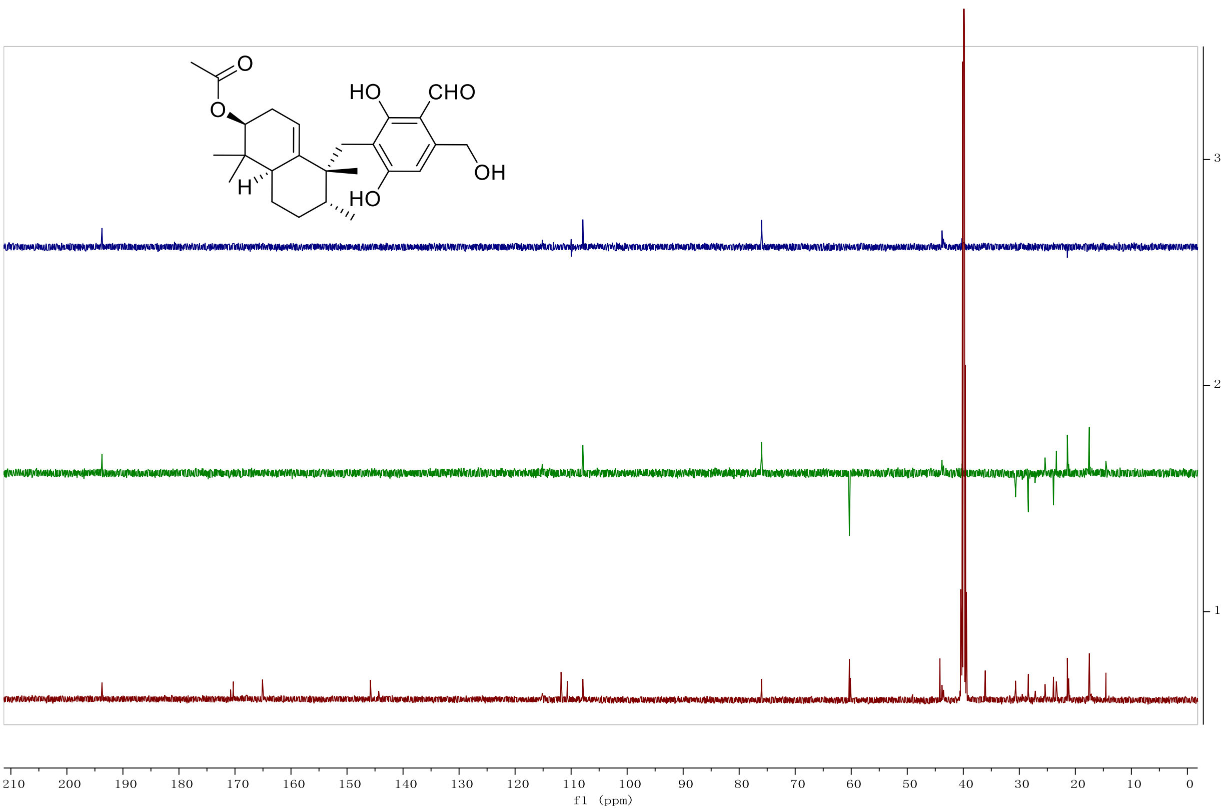

Figure S22. HSQC spectrum of myrothecisin C (**3**) in DMSO-d_6

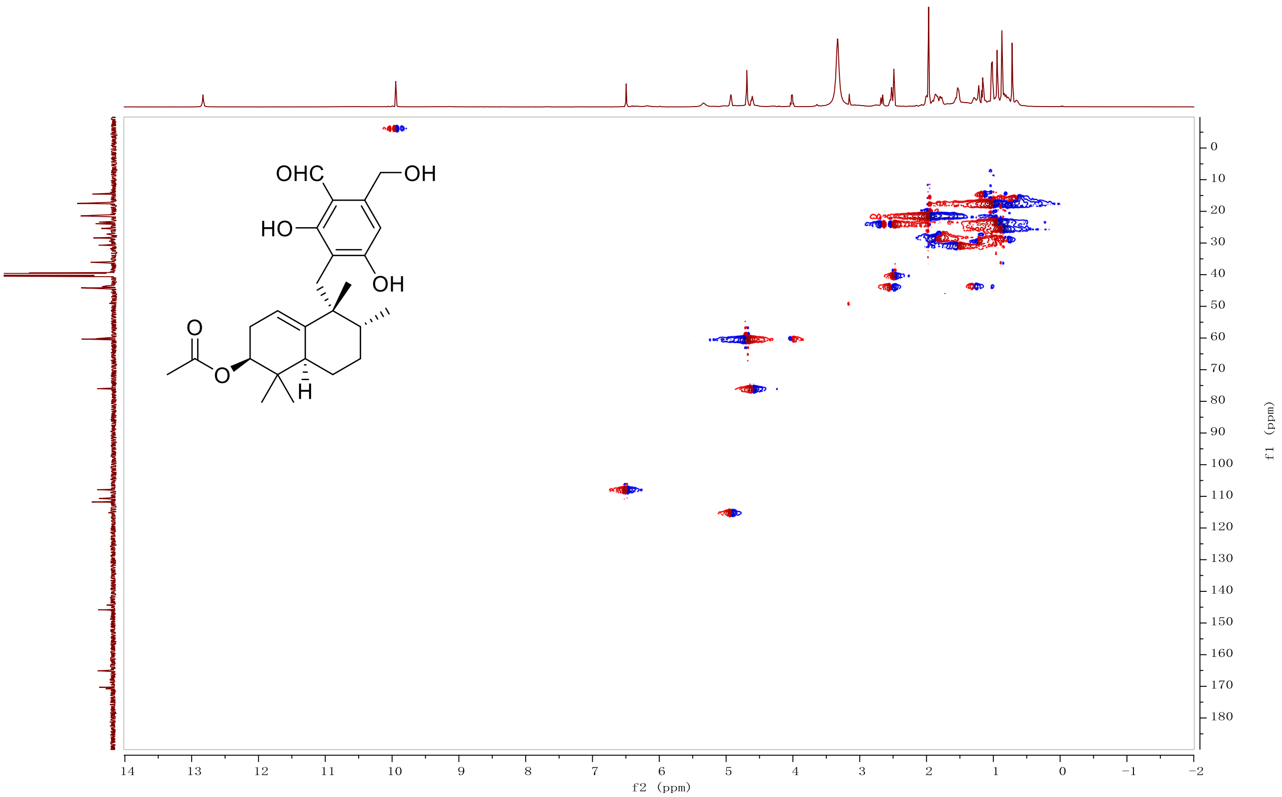

Figure S23. ^{1}H-^{1}H COSY spectrum of myrothecisin C (**3**) in DMSO-d_6

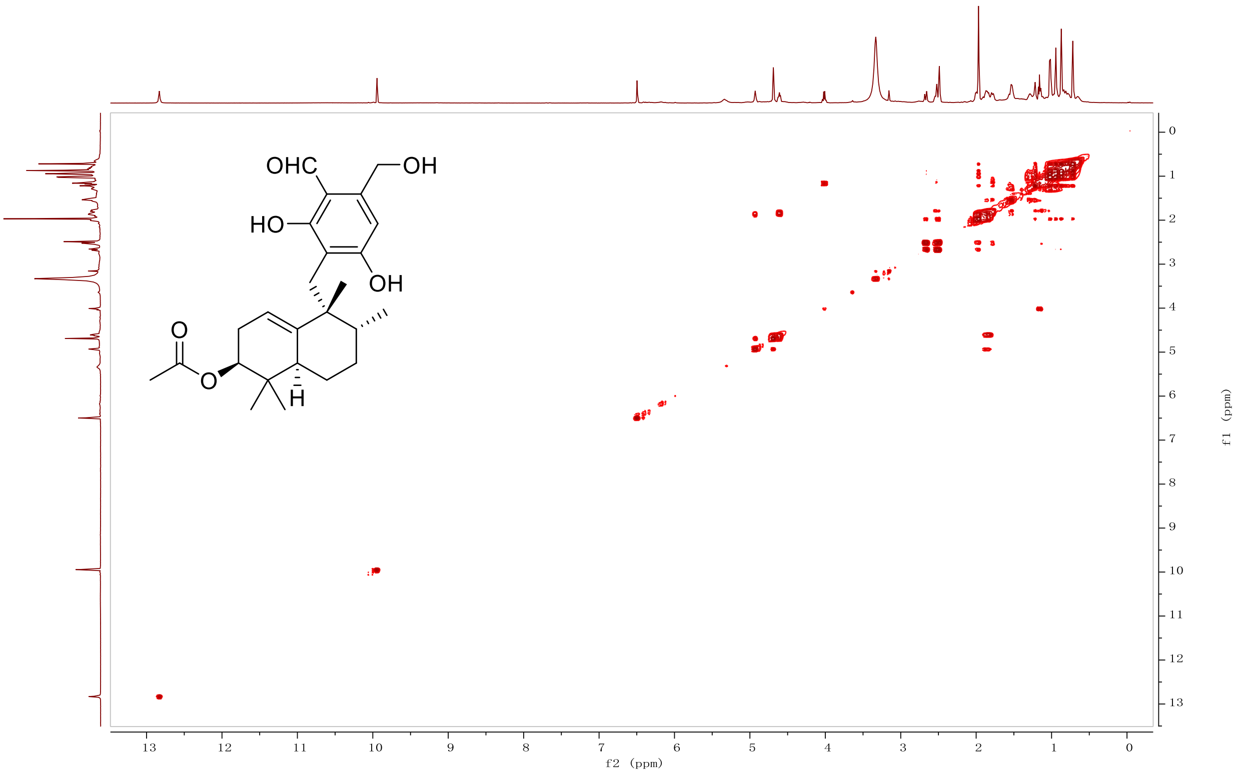

Figure S24. HMBC spectrum of myrothecisin C (**3**) in DMSO-d_6

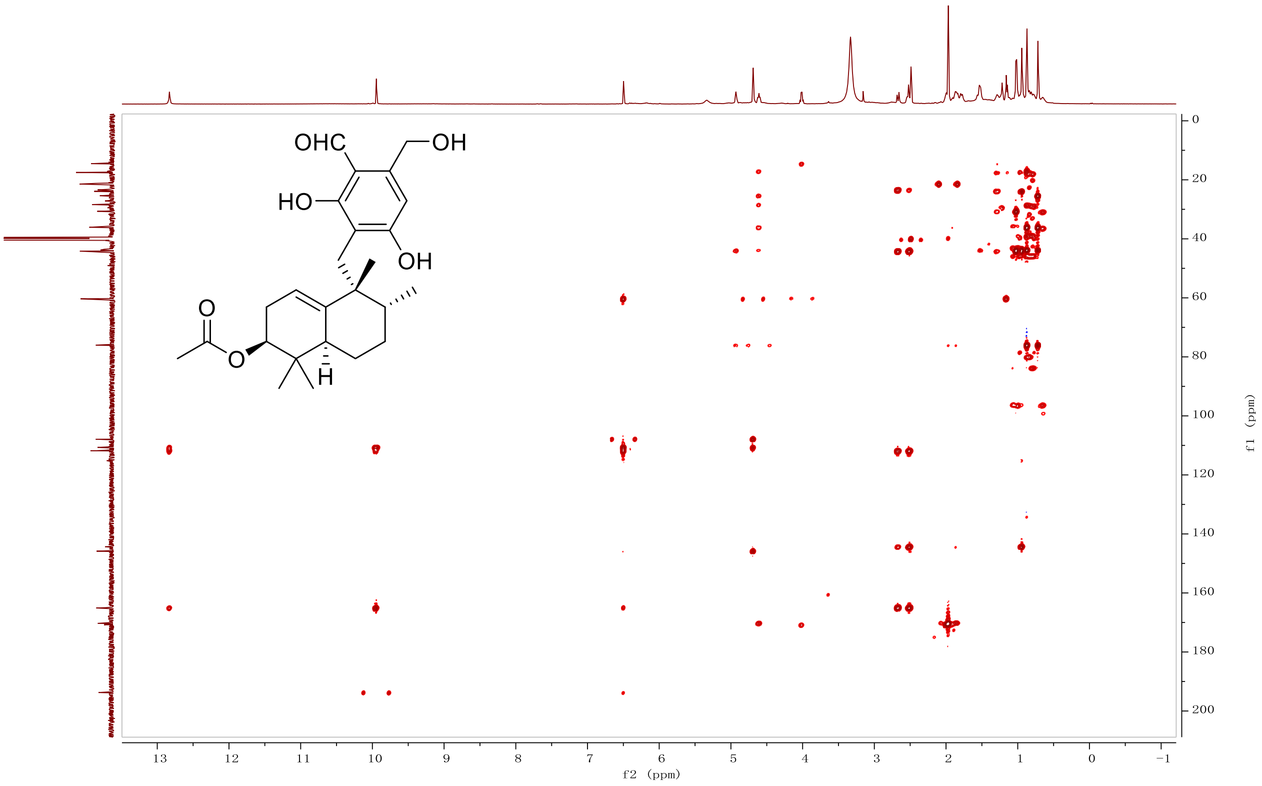

Figure S25. NOESY spectrum of myrothecisin C (**3**) in DMSO-d_6

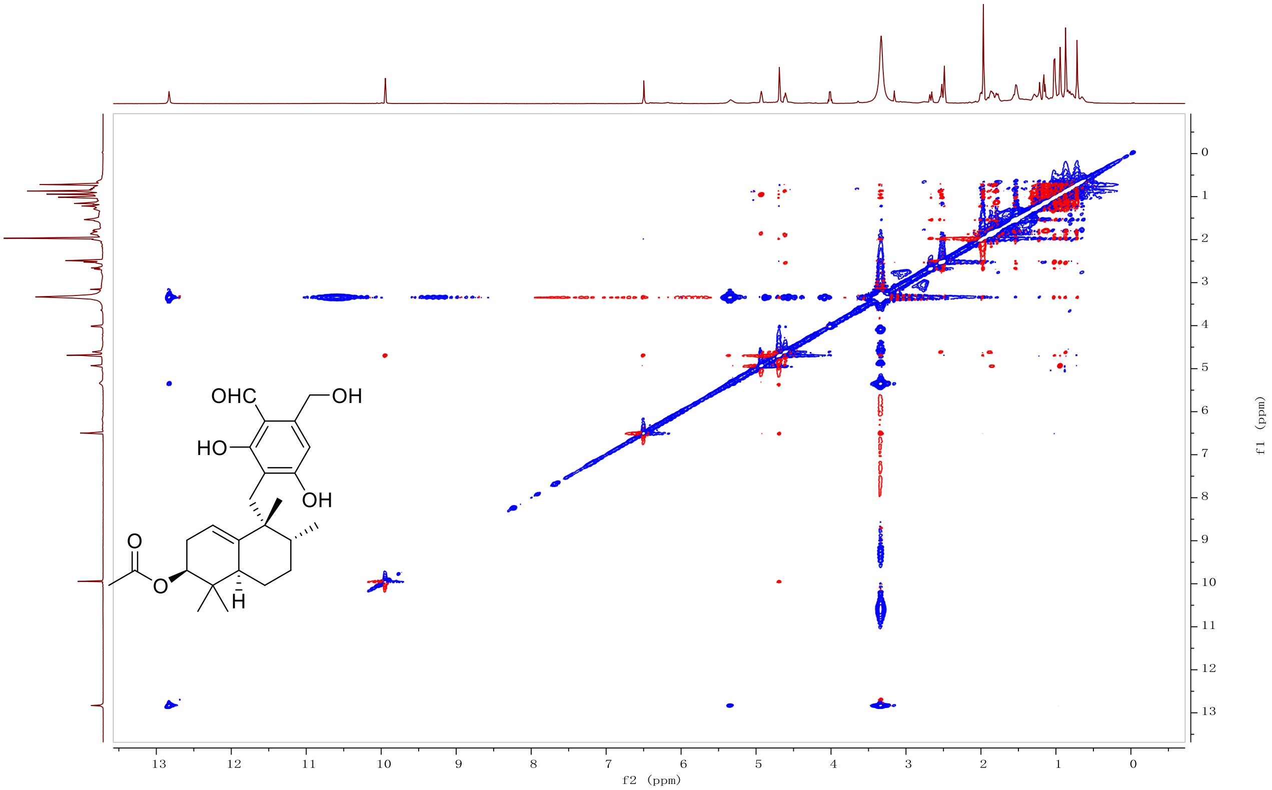

Figure S26. ^{1}H-NMR spectrum of myrothecisin D (**4**) in DMSO-d_6

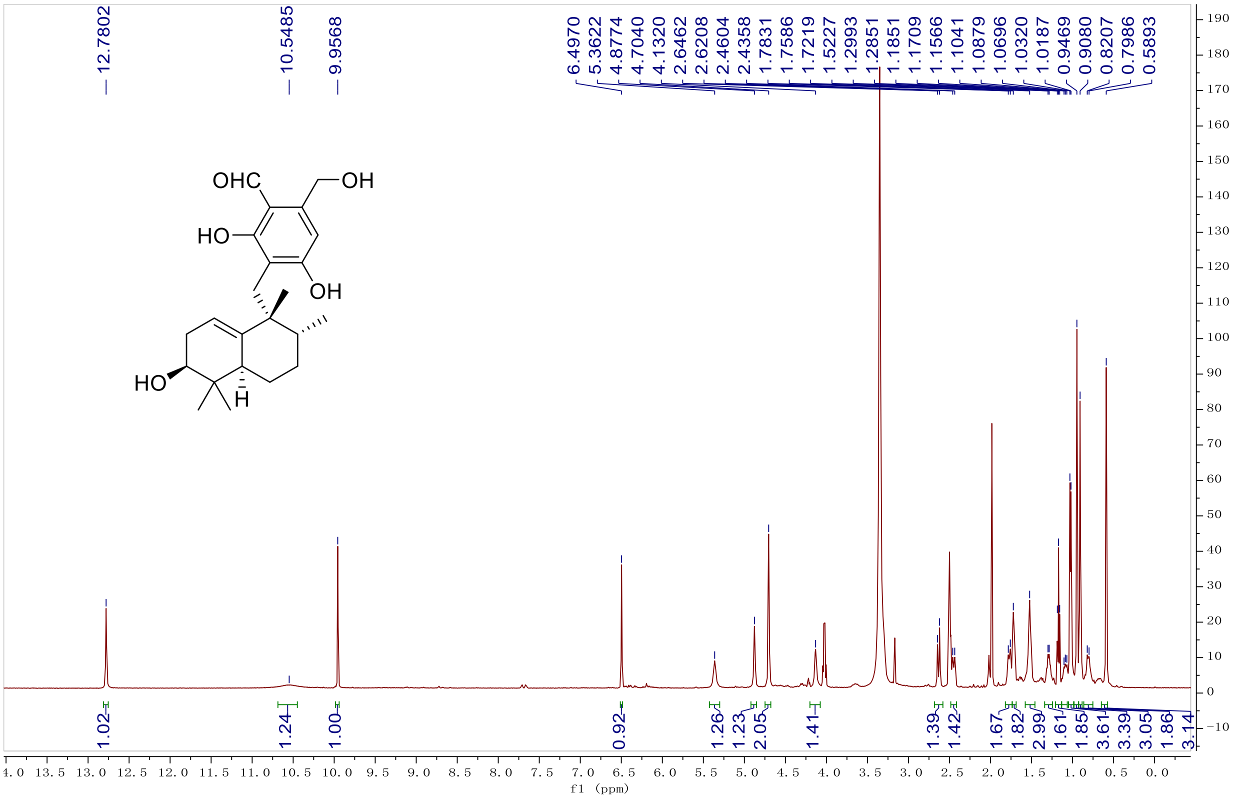

Figure S27. ^{13}C-NMR spectrum of myrothecisin D (**4**) in DMSO-d_6

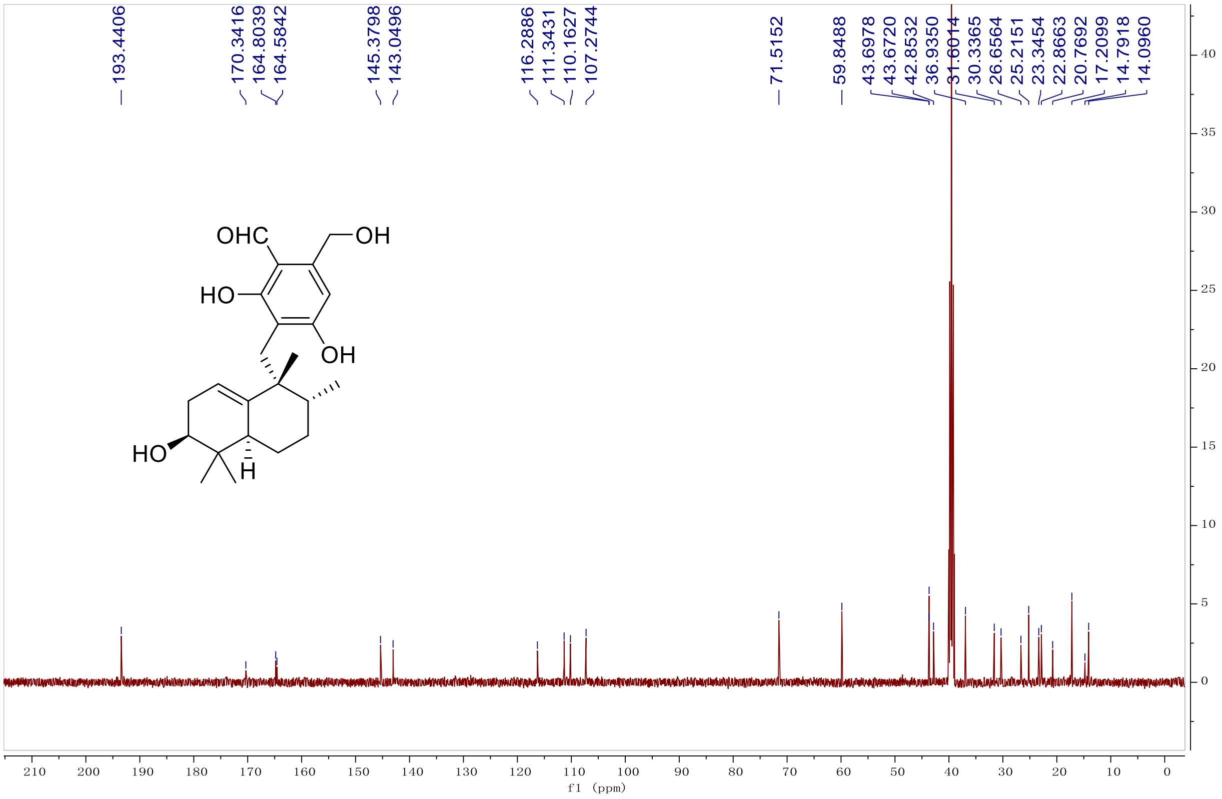

Figure S28. DEPT spectrum of myrothecisin D (**4**) in DMSO-d_6

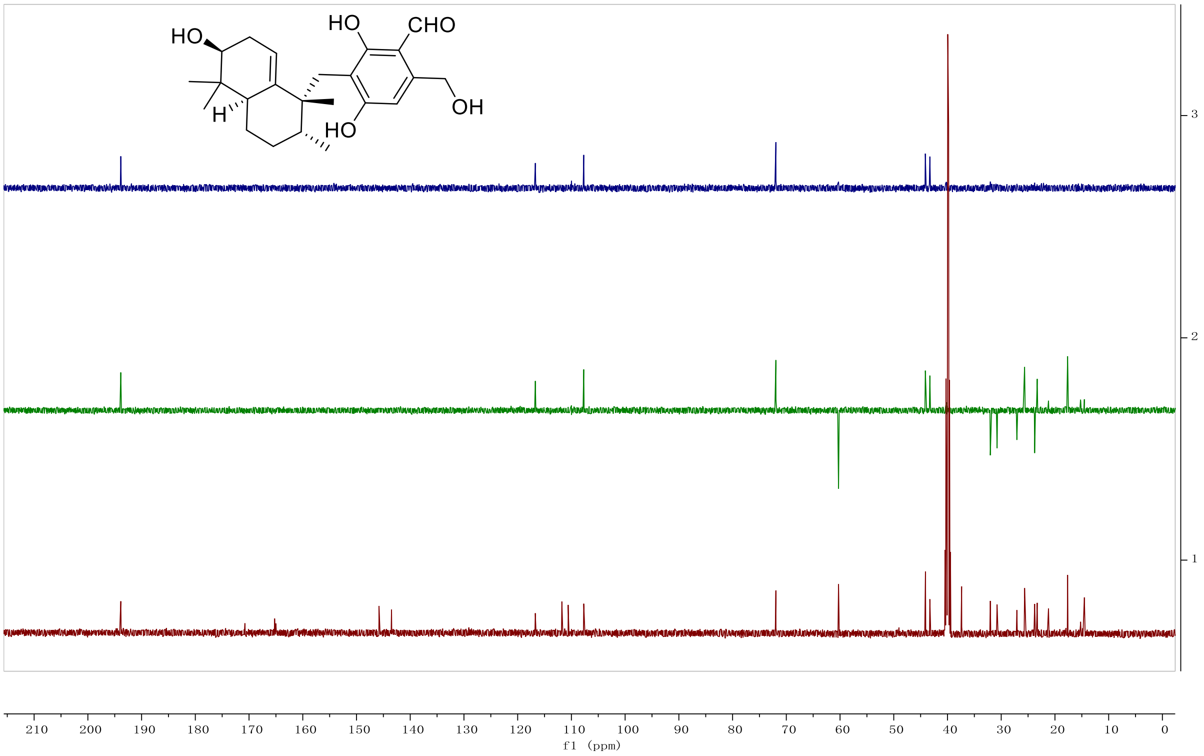

Figure S29. HSQC spectrum of myrothecisin D (**4**) in DMSO-d_6

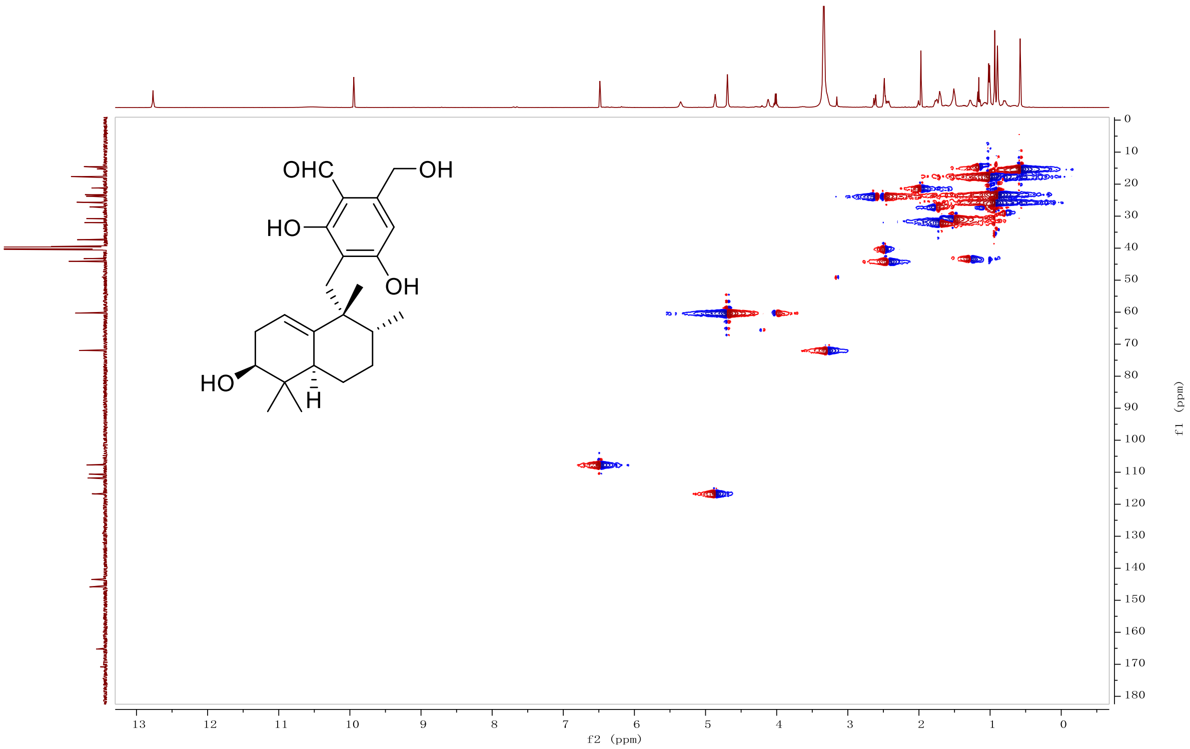

Figure S30. ^{1}H-^{1}H COSY spectrum of myrothecisin D (**4**) in DMSO-d_6

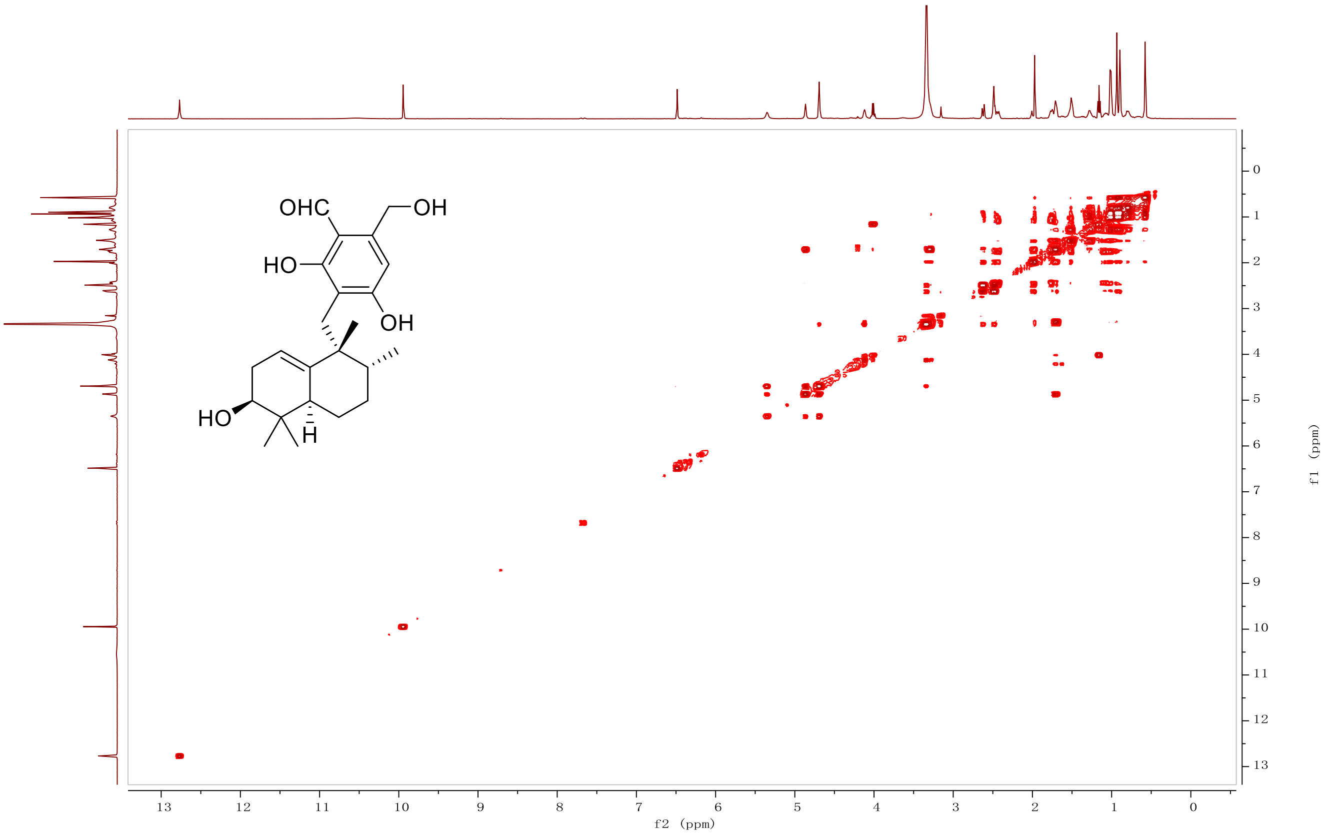

Figure S31. HMBC spectrum of myrothecisin D (**4**) in DMSO-d_6

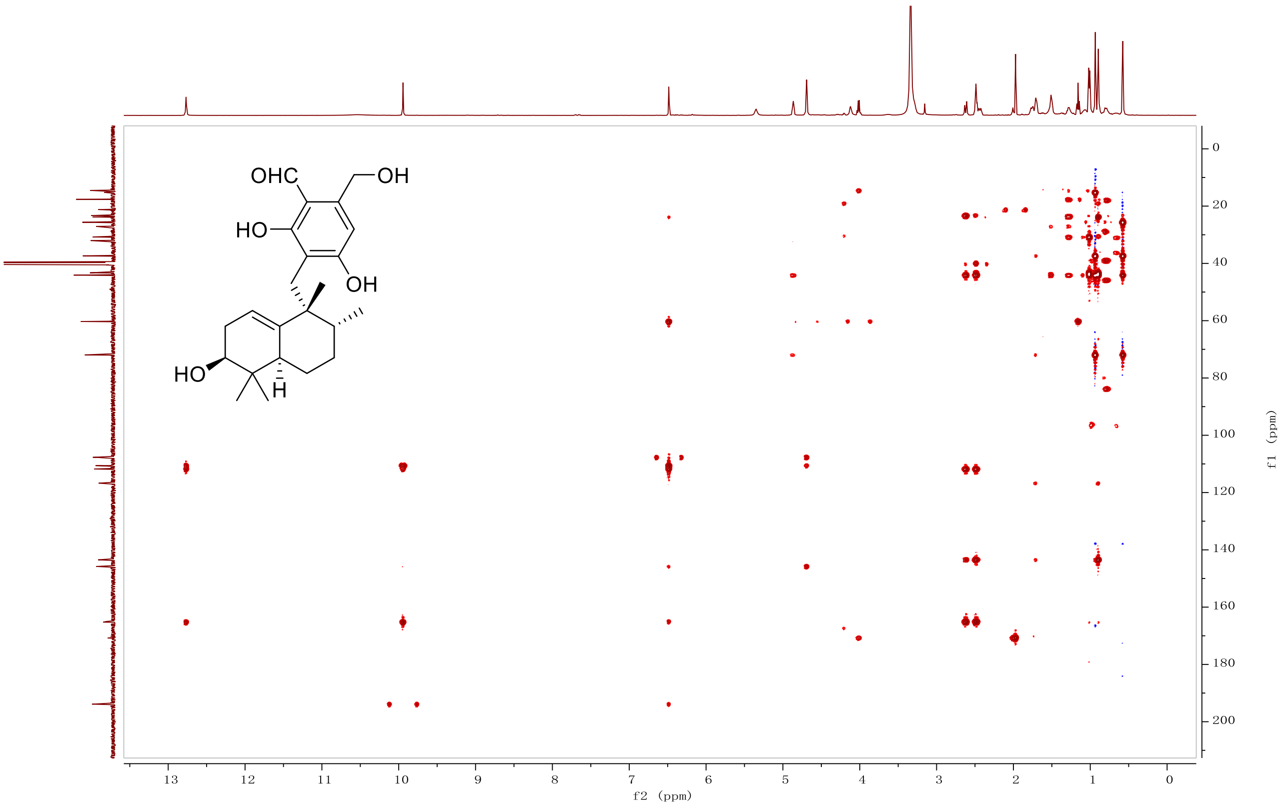

Figure S32. NOESY spectrum of myrothecisin D (**4**) in DMSO-d_6

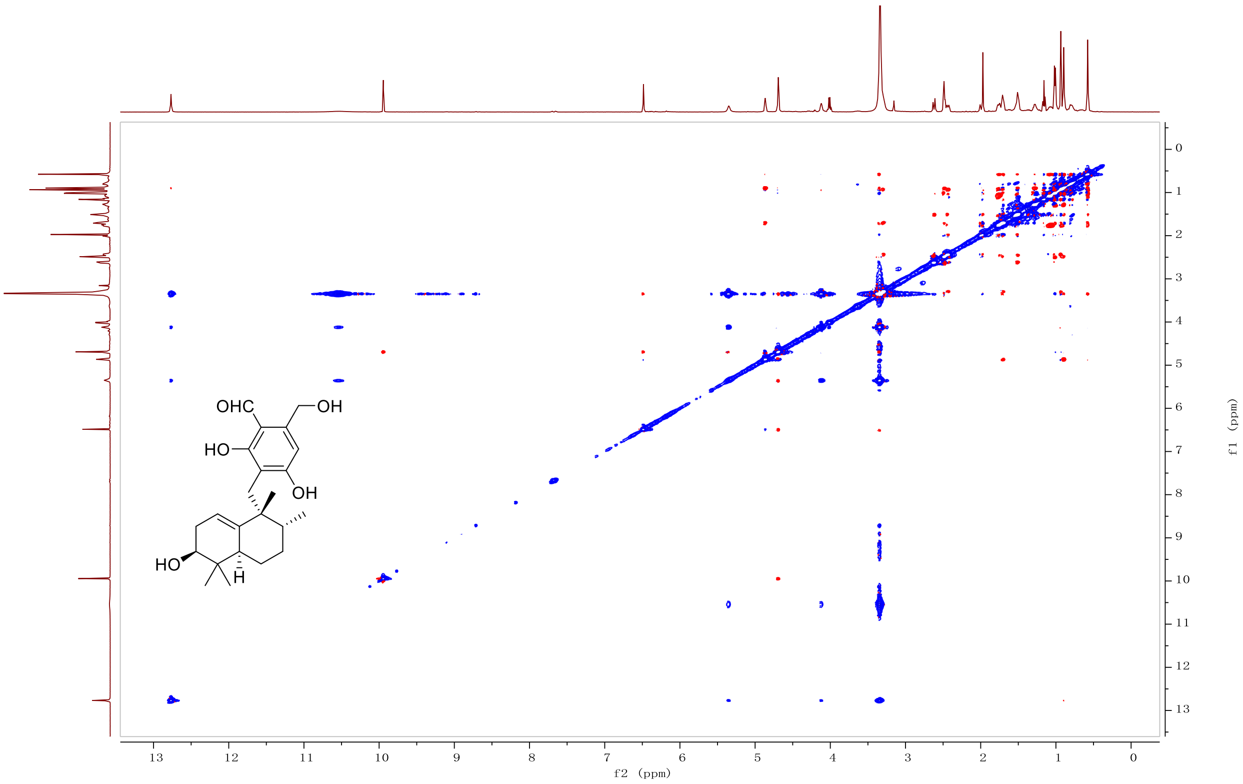

Figure S33. ^{1}H-NMR spectrum of myrothelactone A (**5**) in DMSO-d_6

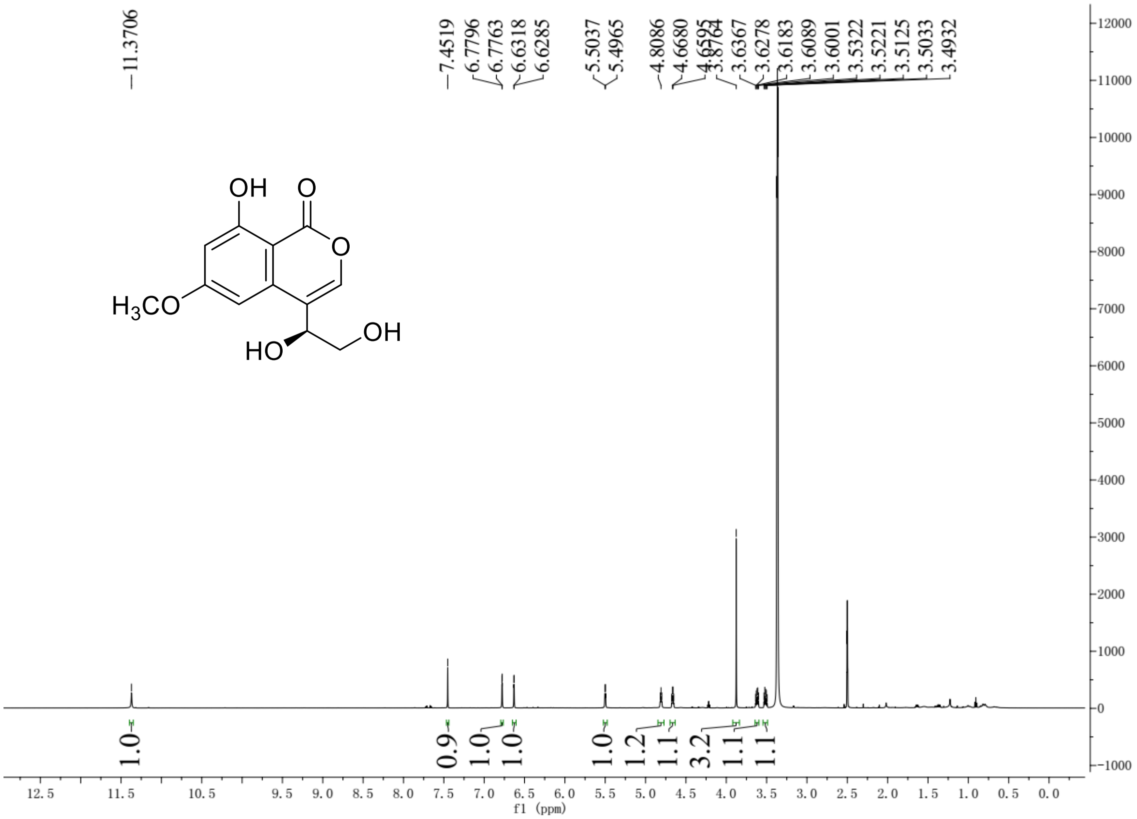

Figure S34. ^{13}C-NMR spectrum of myrothelactone A (**5**) in DMSO-d_6

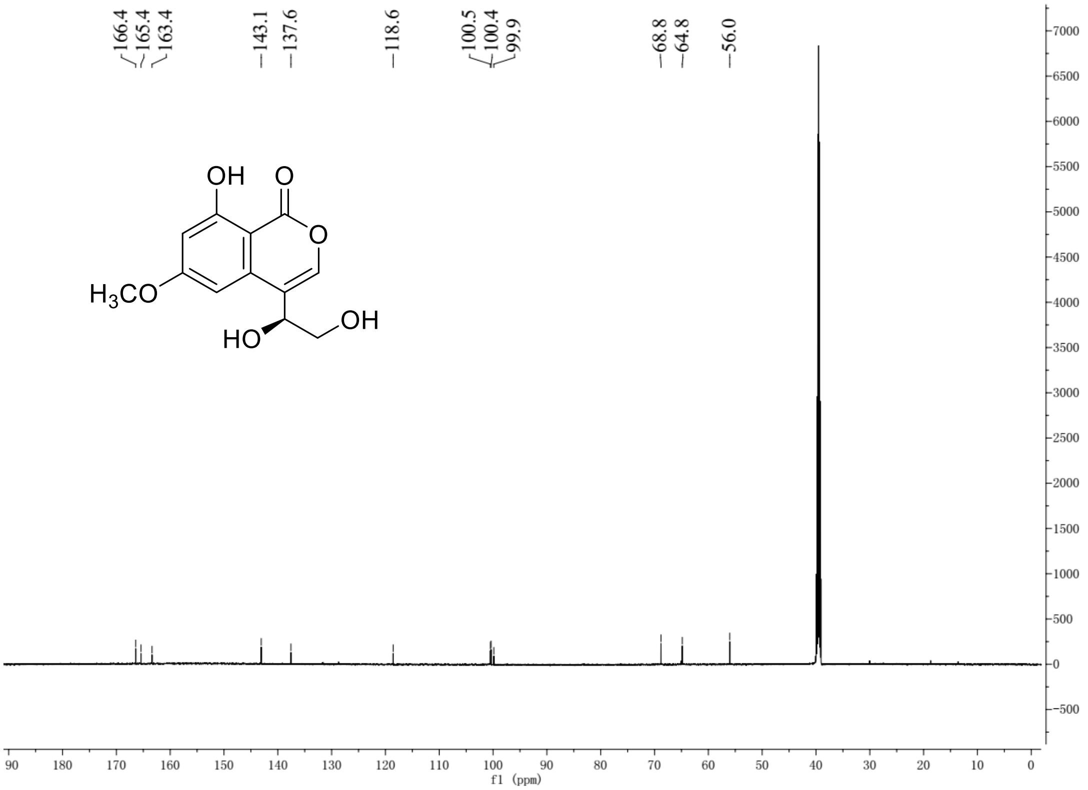

Figure S35. DEPT spectrum of myrothelactone A (**5**) in DMSO-d_6

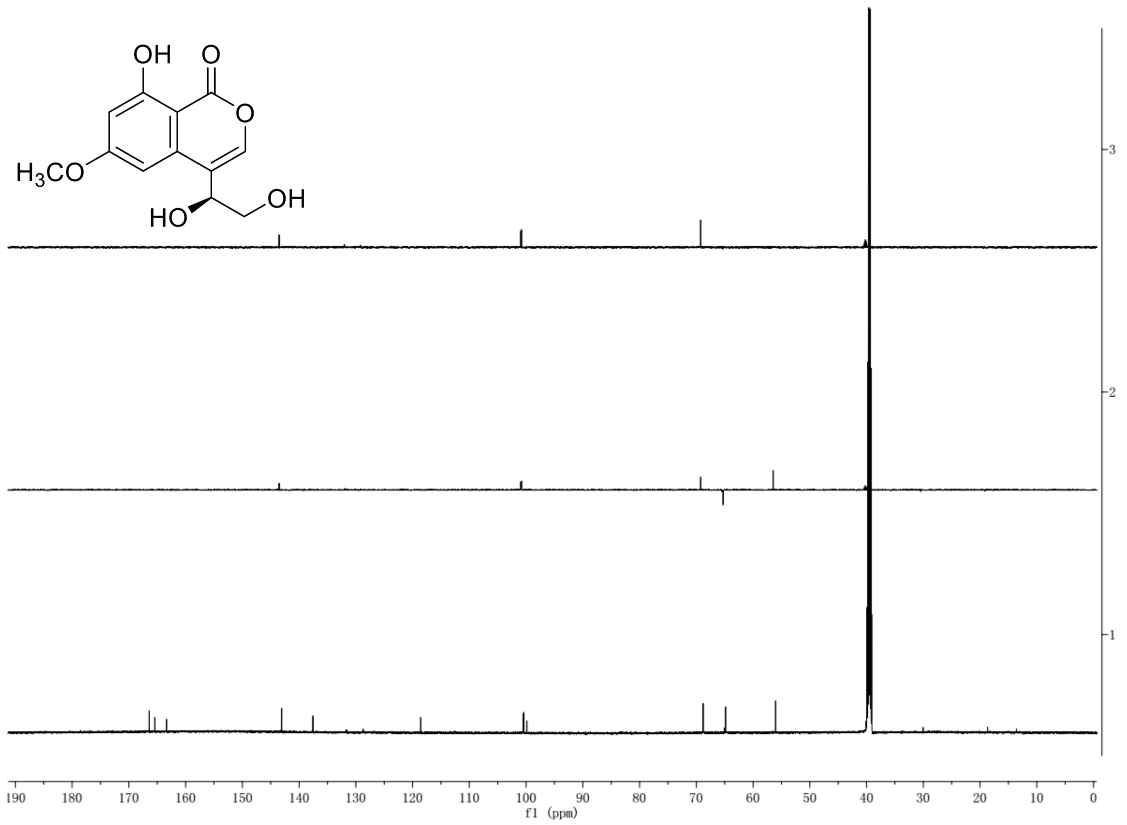

Figure S36. HSQC spectrum of myrothelactone A (**5**) in DMSO-d_6

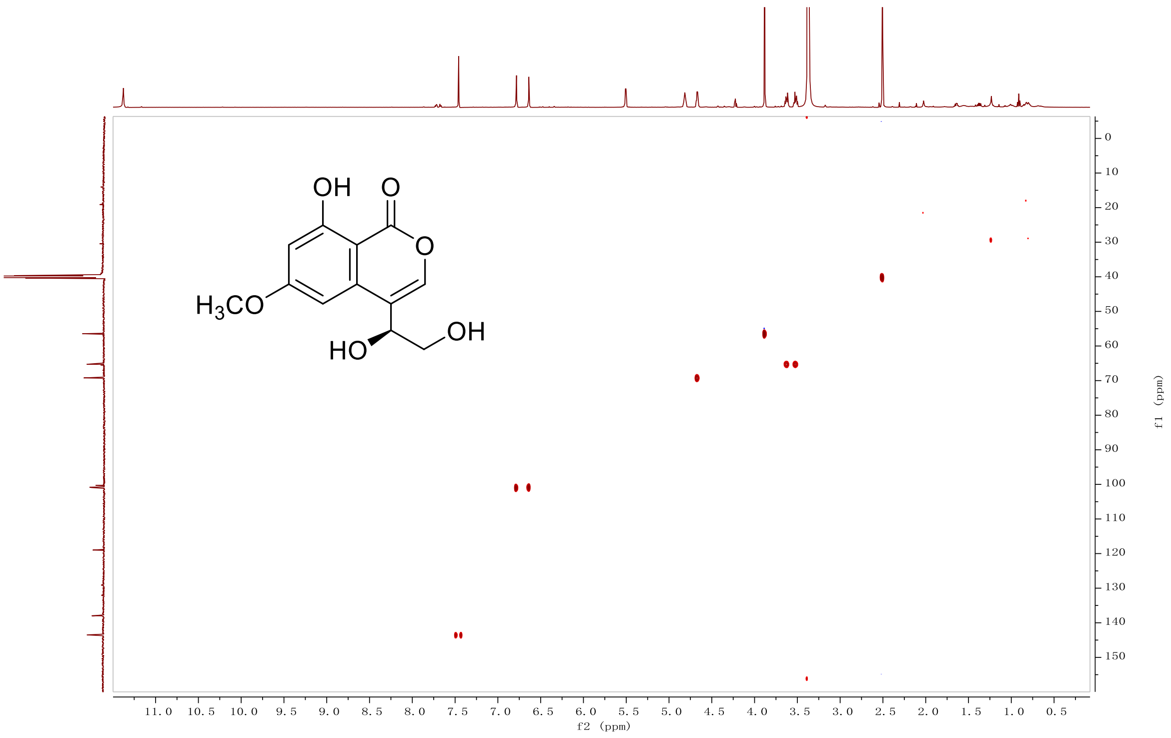

Figure S37. ^{1}H-^{1}H COSY spectrum of myrothelactone A (**5**) in DMSO-d_6

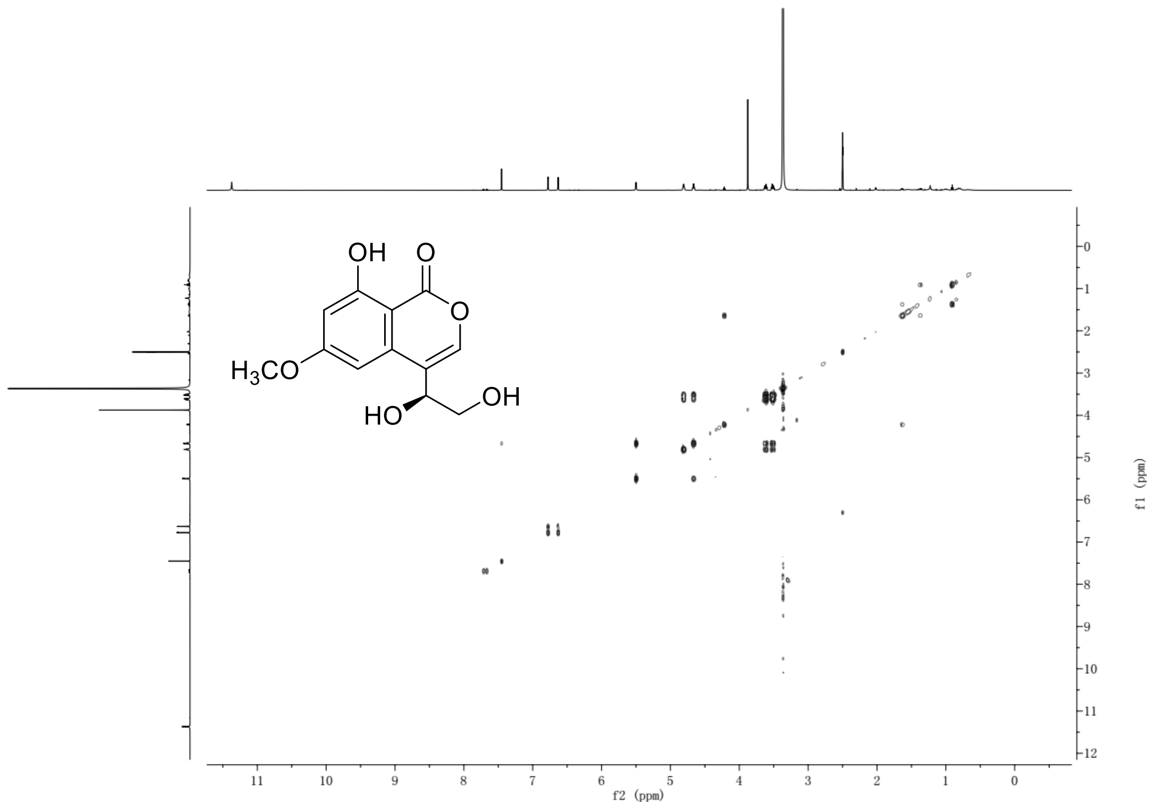

Figure S38. HMBC spectrum of myrothelactone A (**5**) in DMSO-d_6

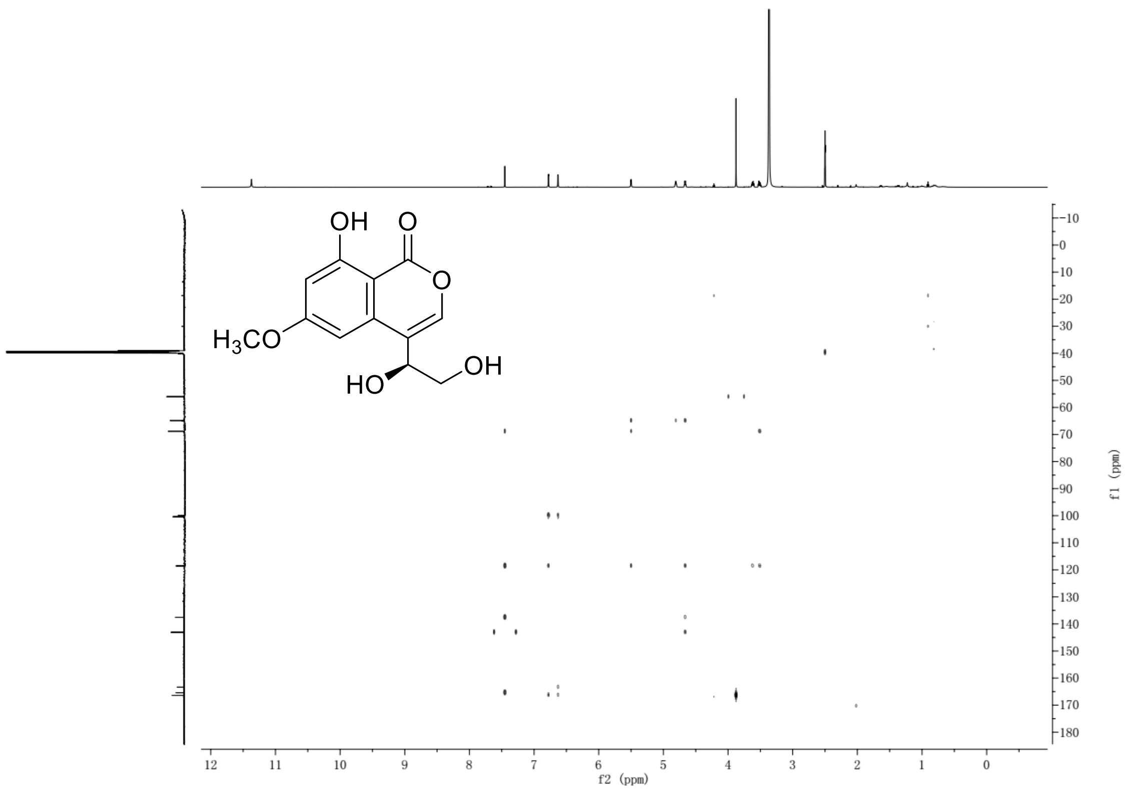

Figure S39. ^{1}H-NMR spectrum of myrothelactone B (**6**) in DMSO-d_6

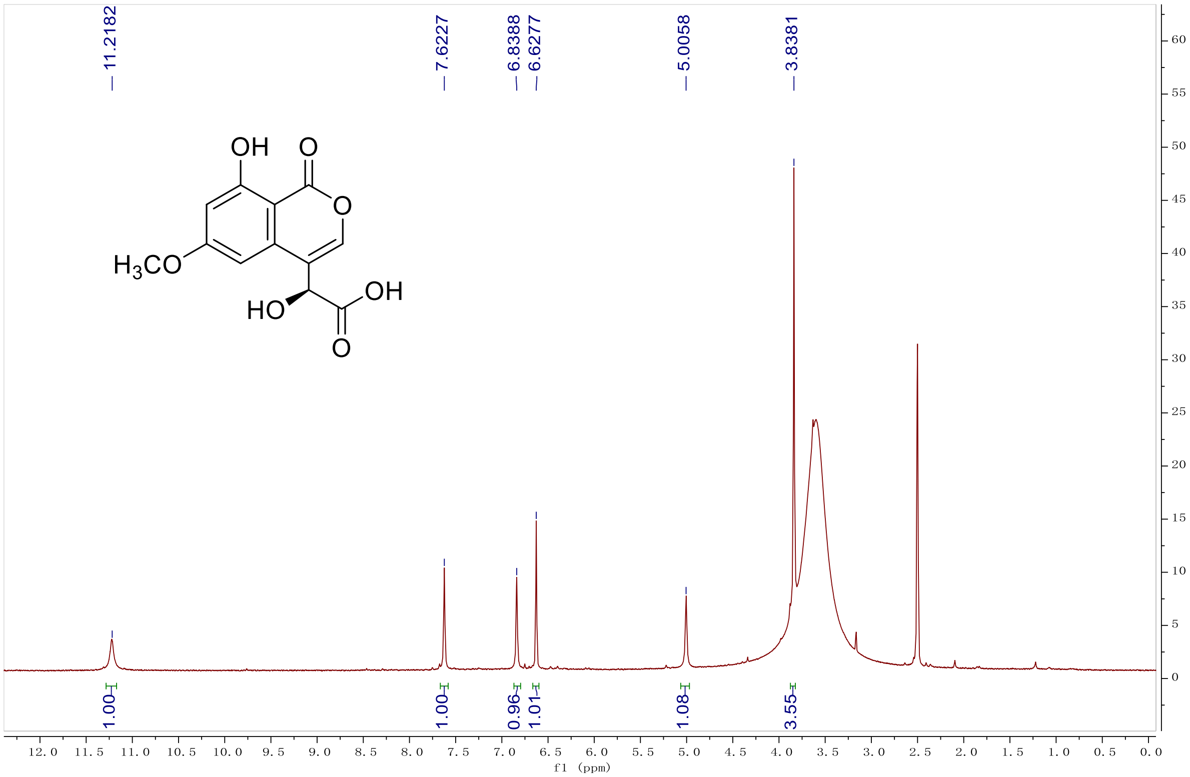

Figure S40. ^{13}C-NMR spectrum of myrothelactone B (**6**) in DMSO-d_6

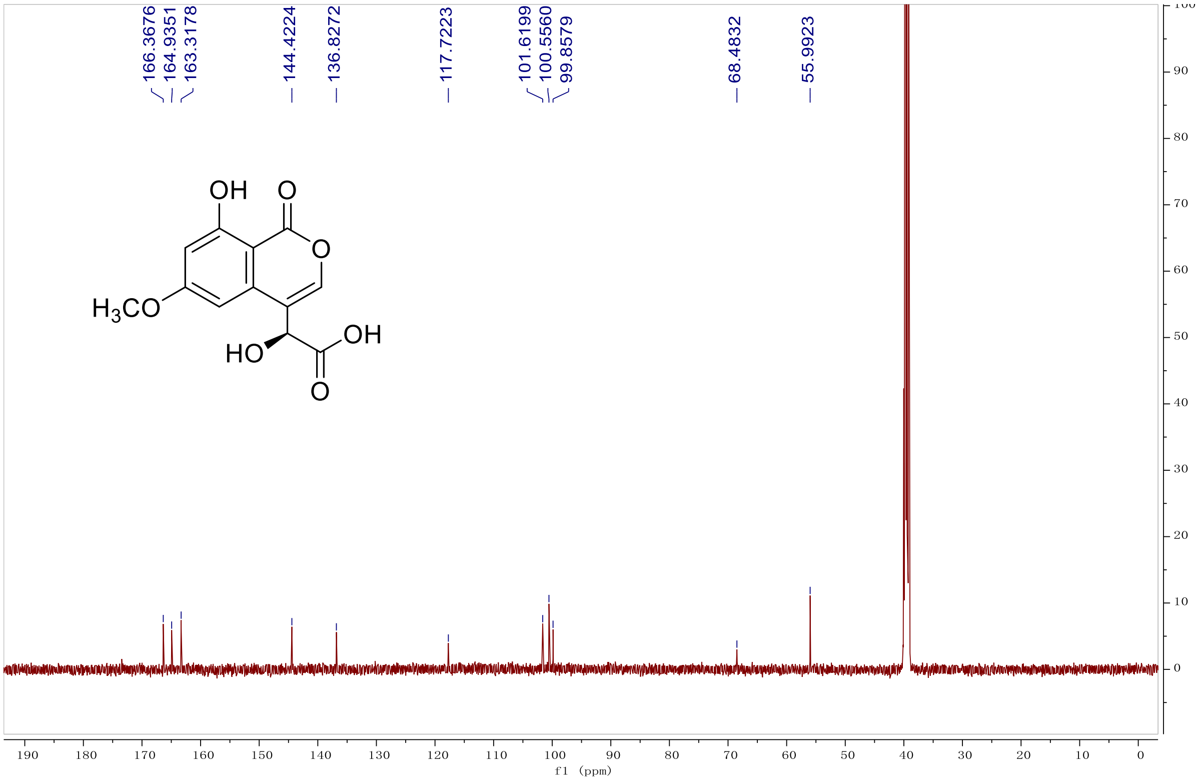

Figure S41. DEPT spectrum of myrothelactone B (**6**) in DMSO-d_6

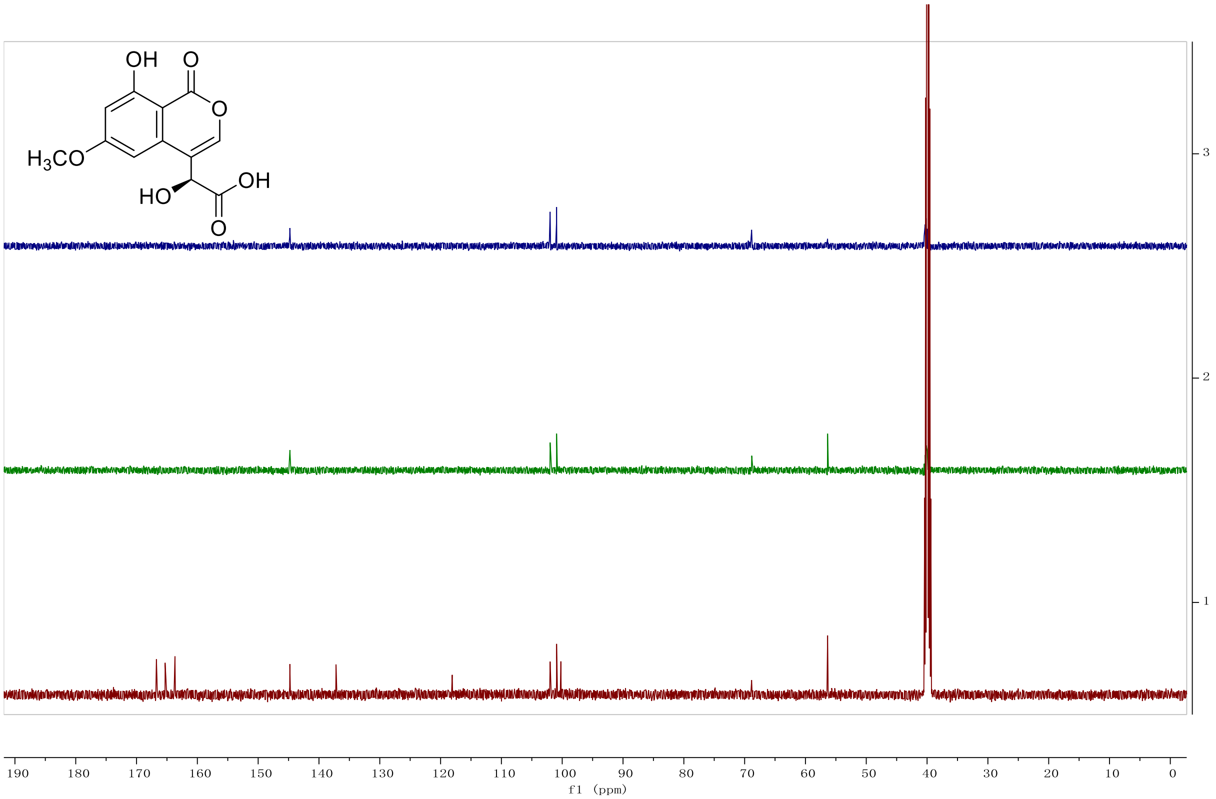

Figure S42. HSQC spectrum of myrothelactone B (**6**) in DMSO-d_6

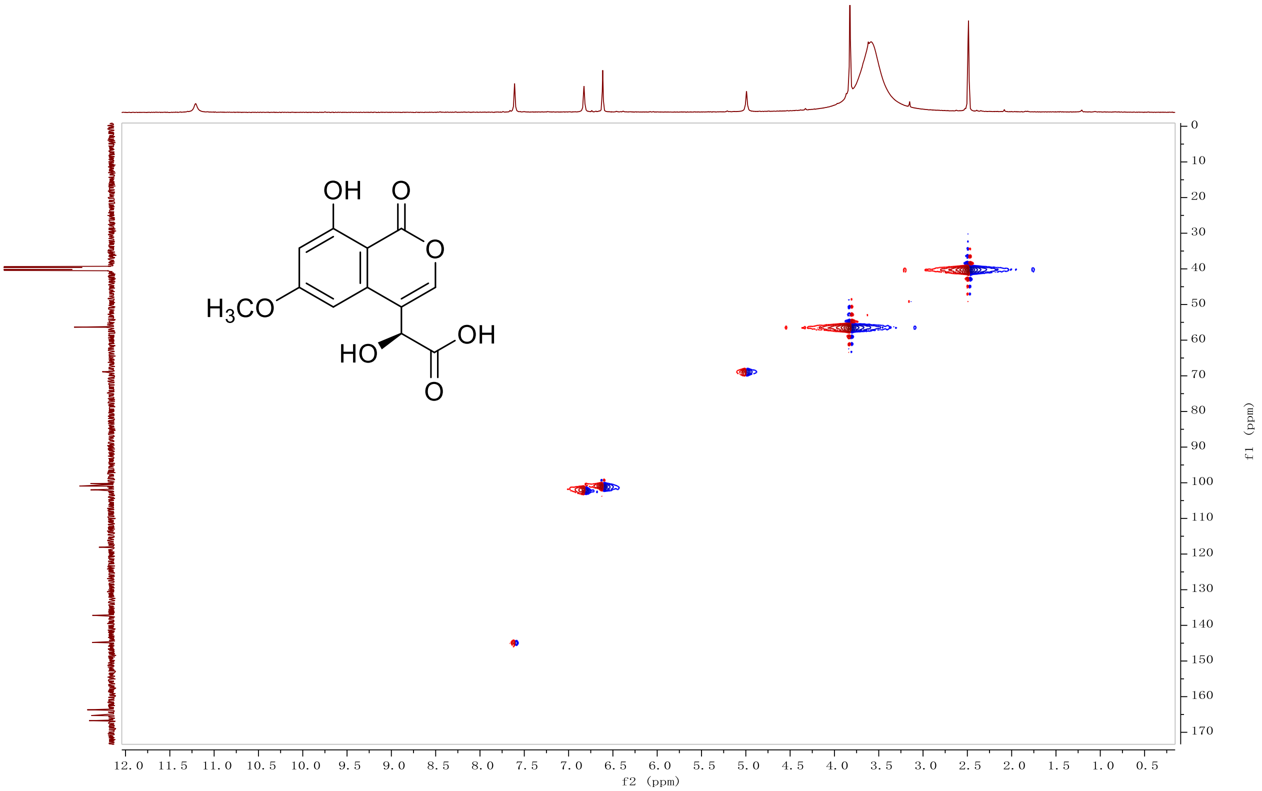

Figure S43. ^{1}H-^{1}H COSY spectrum of myrothelactone B (**6**) in DMSO-d_6

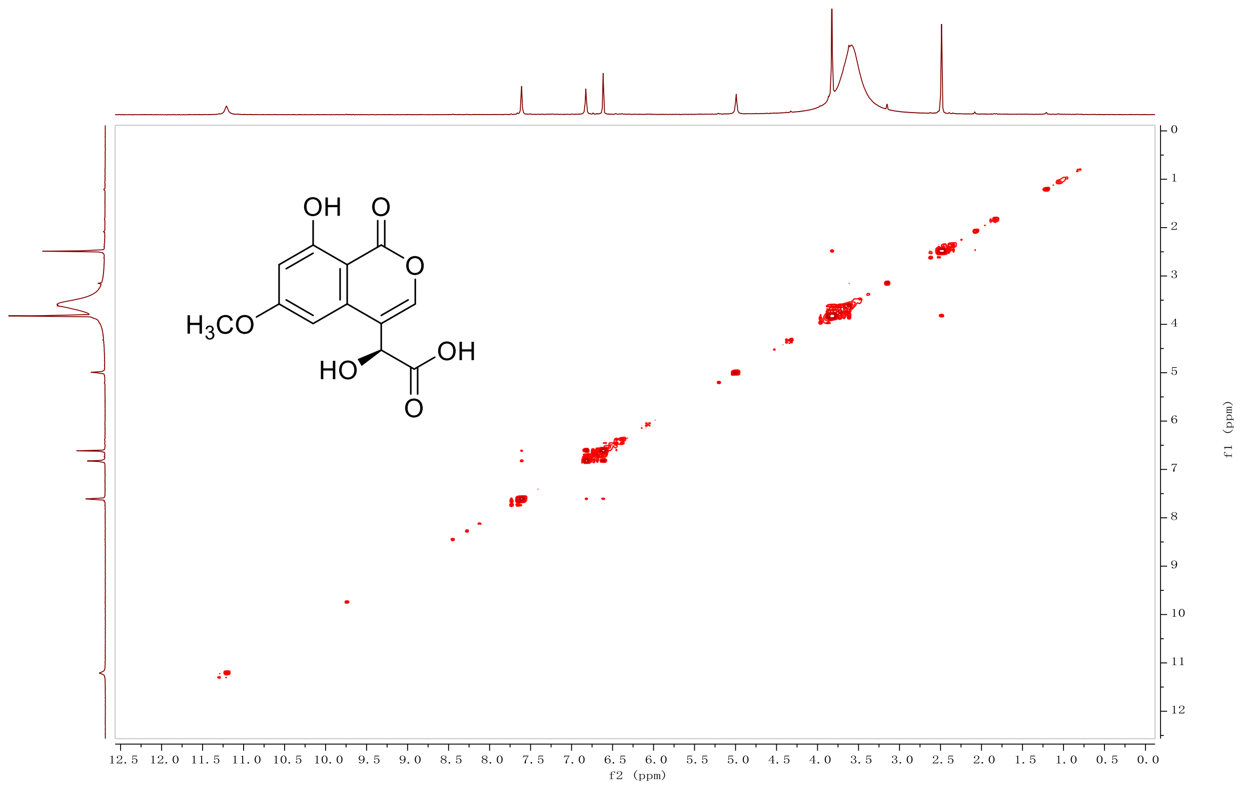

Figure S44. HMBC spectrum of myrothelactone B (**6**) in DMSO-d_6

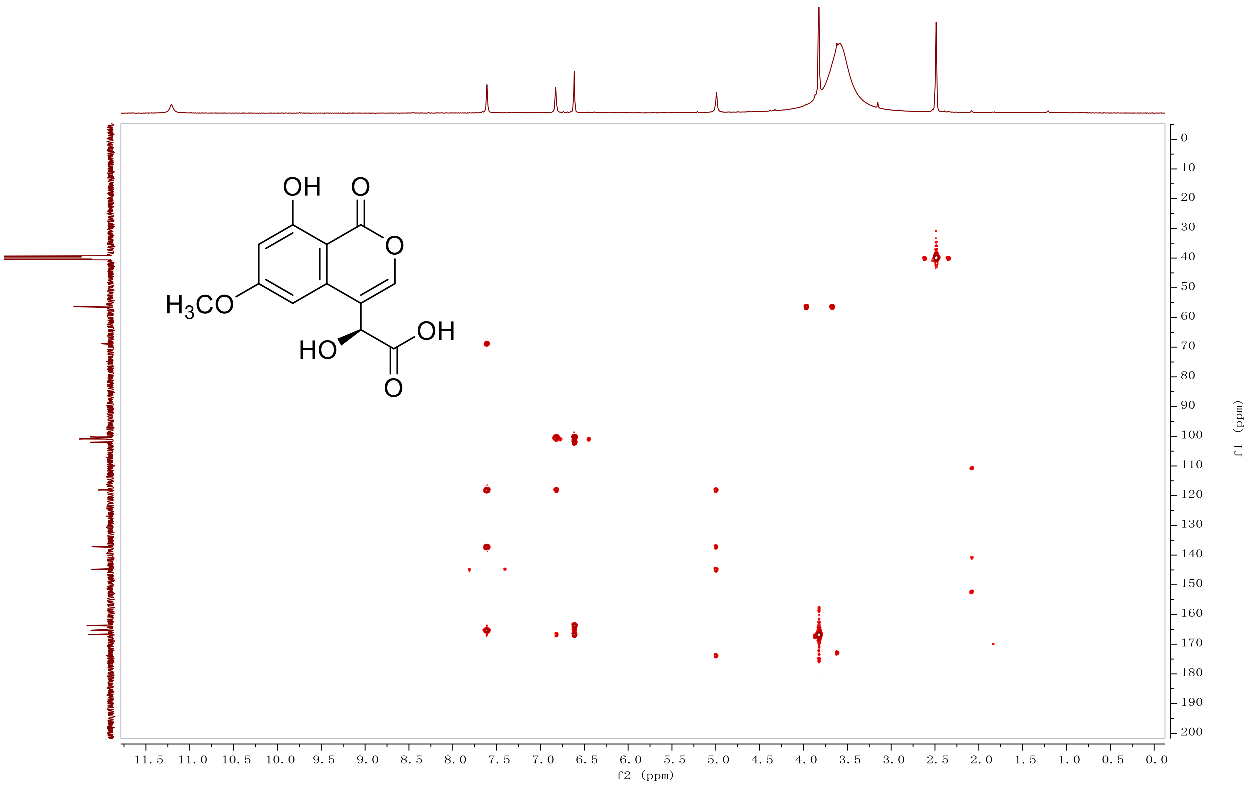

Figure S45. ^{1}H-NMR spectrum of myrothelactone C (**7**) in DMSO-d_6

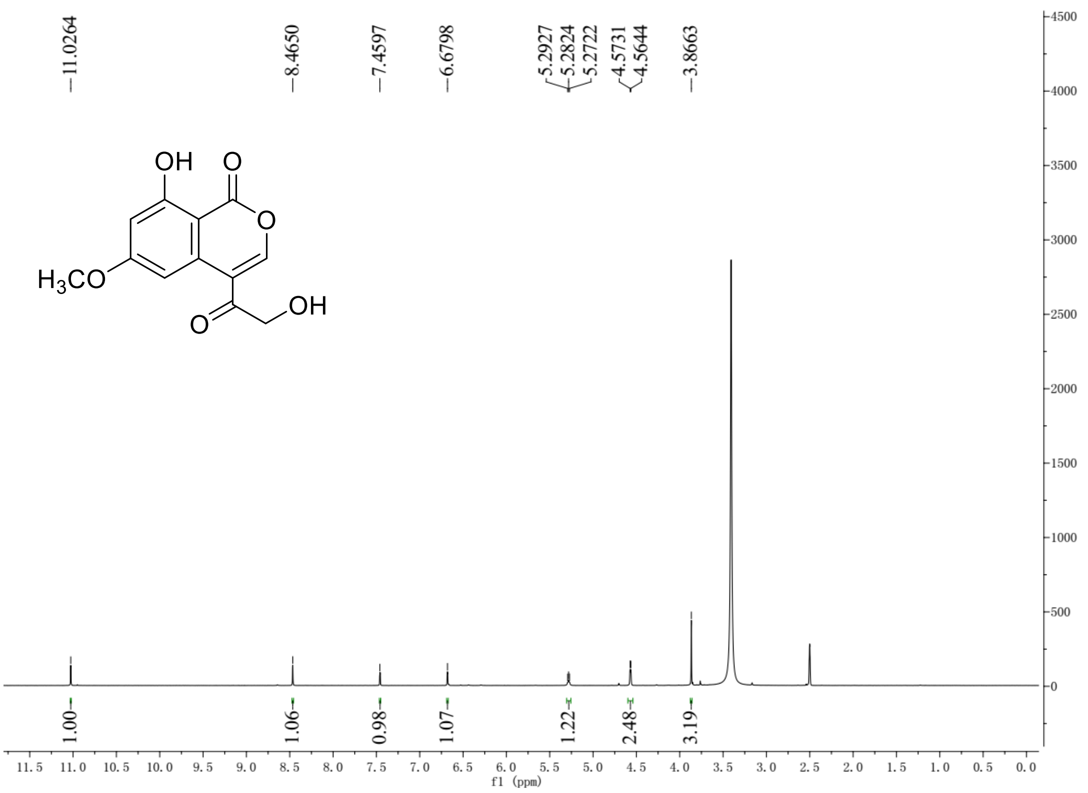

Figure S46. ^{13}C-NMR spectrum of myrothelactone C (**7**) in DMSO-d_6

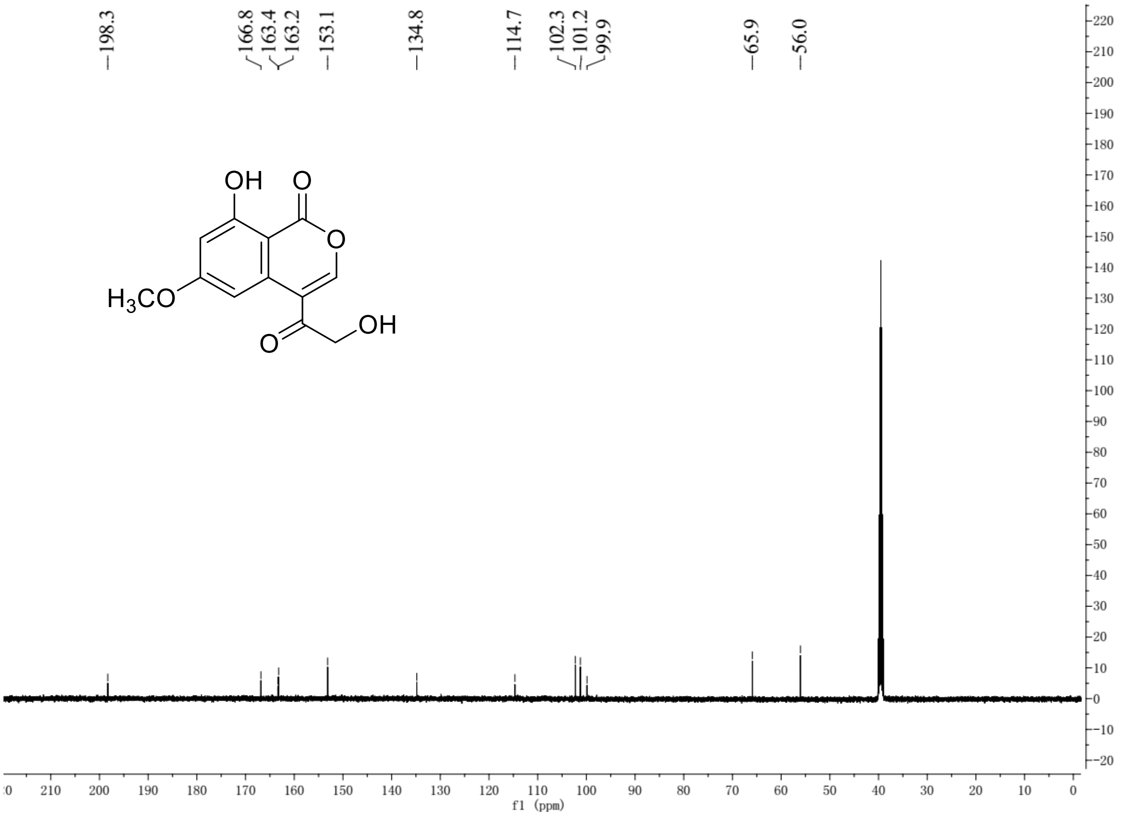

Figure S47. DEPT spectrum of myrothelactone C (**7**) in DMSO-d_6

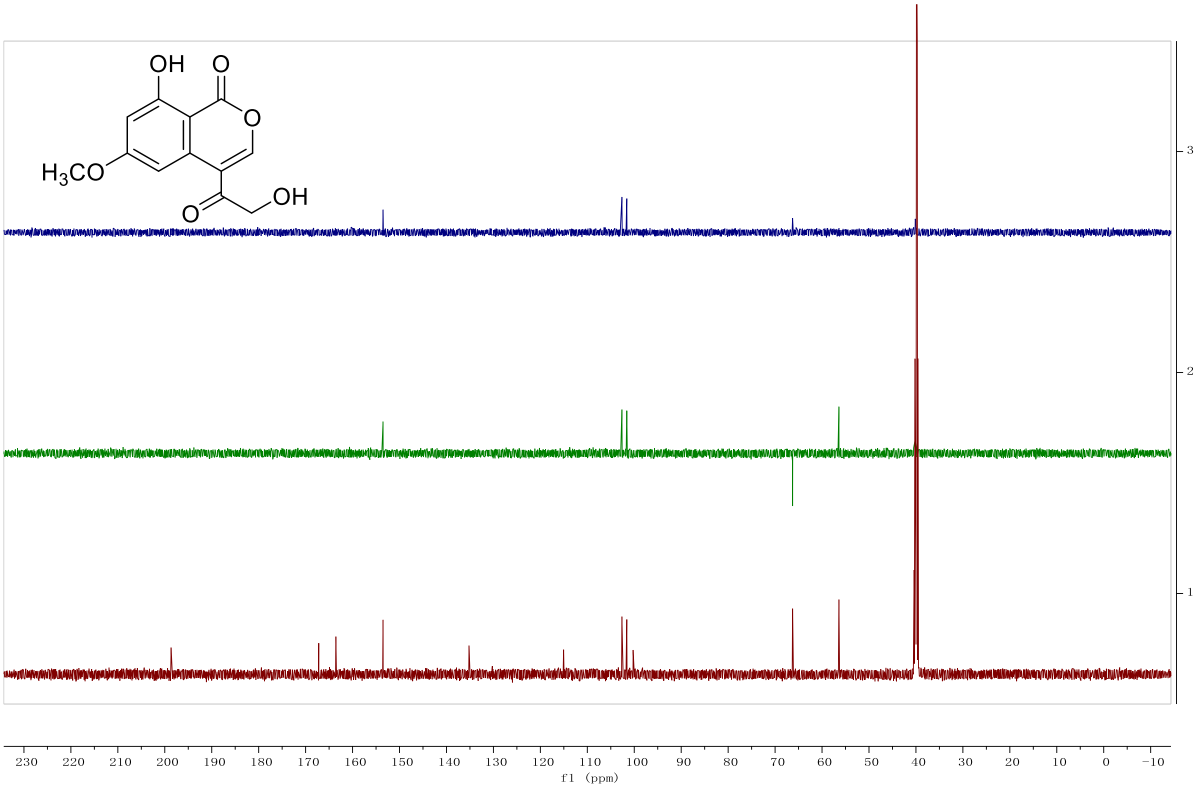

Figure S48. HSQC spectrum of myrothelactone C (**7**) in DMSO-d_6

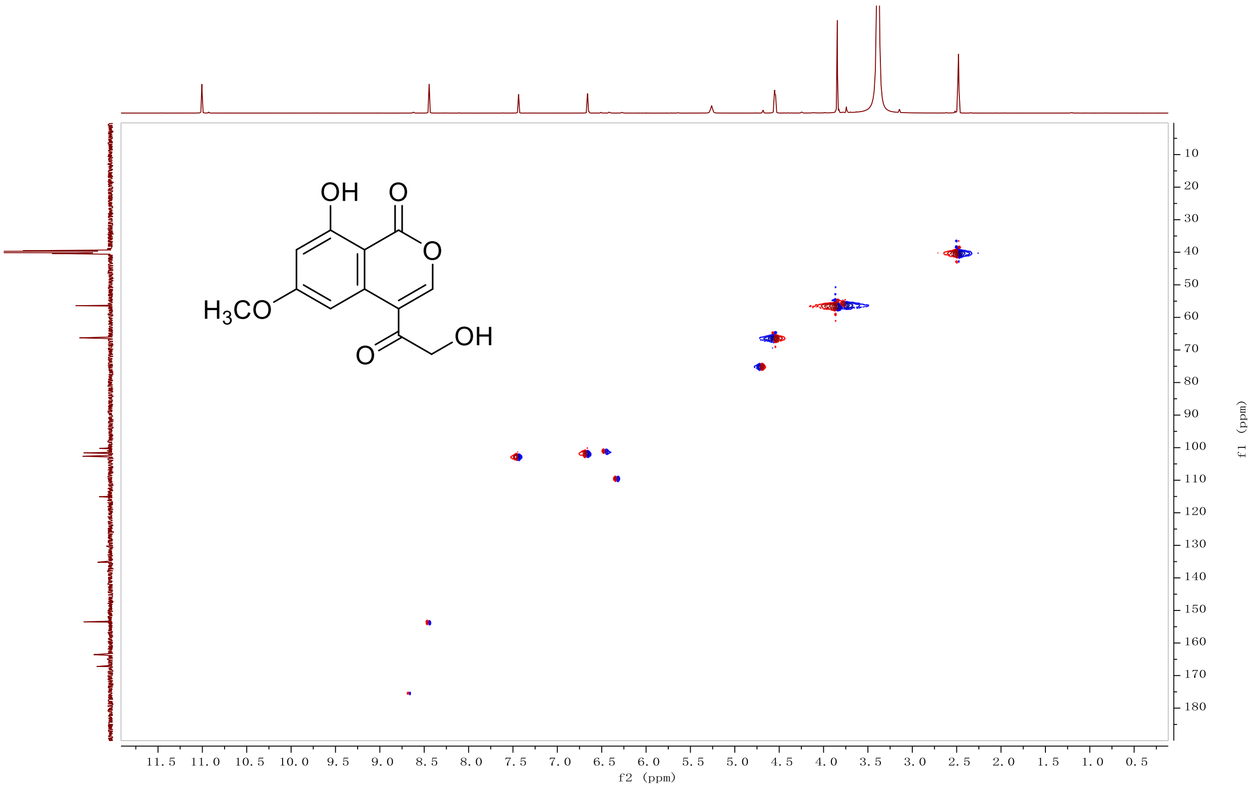

Figure S49. ^{1}H-^{1}H COSY spectrum of myrothelactone C (**7**) in DMSO-d_6

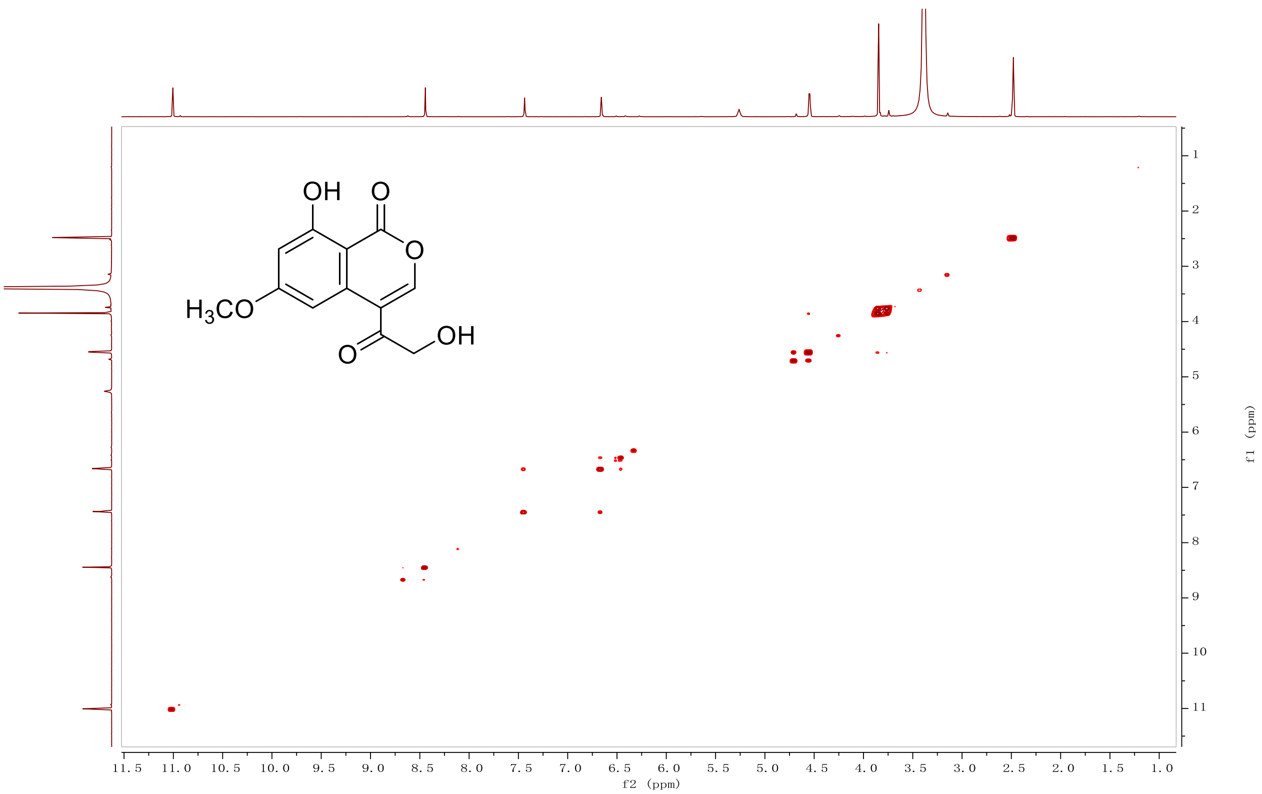

Figure S50. HMBC spectrum of myrothelactone C (**7**) in DMSO-d_6

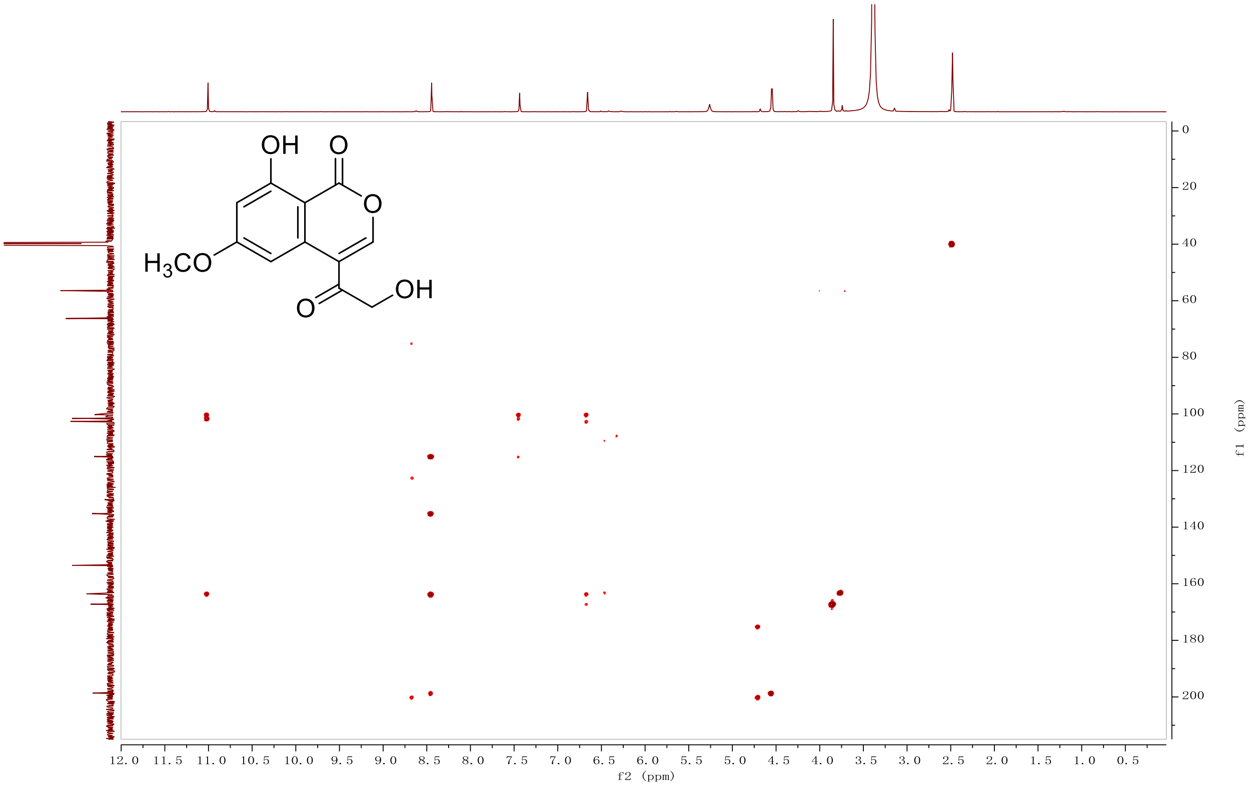

Figure S51. ^{1}H-NMR spectrum of myrothelactone D (**8**) in DMSO-d_6

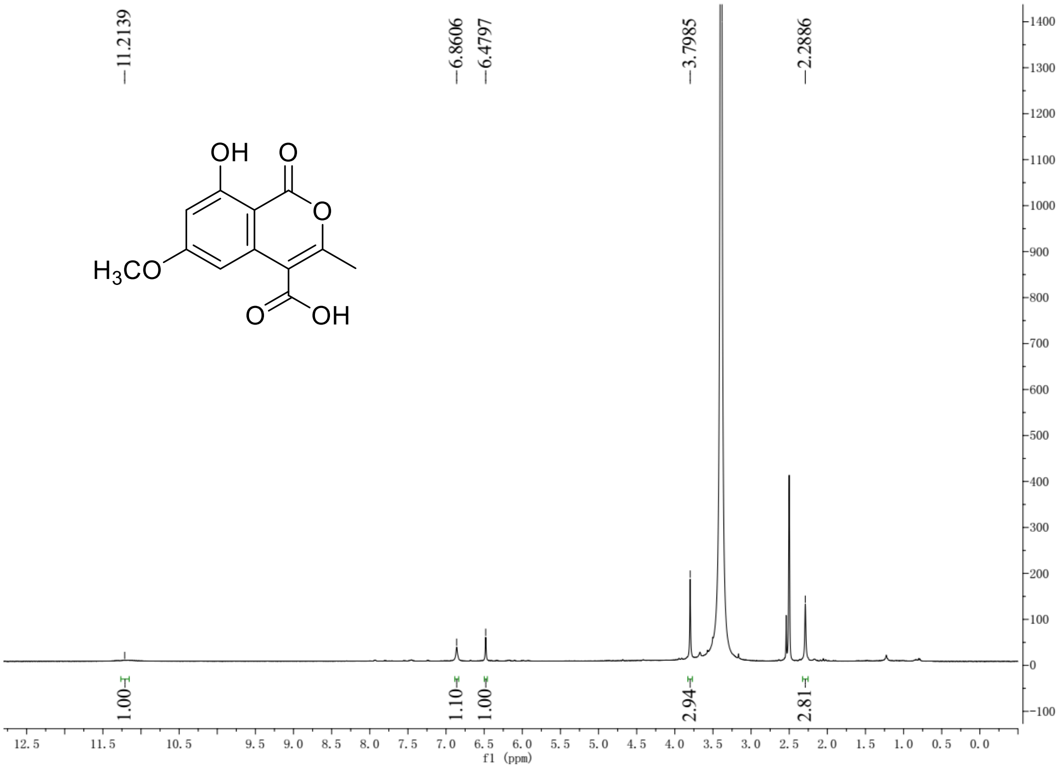

Figure S52. ^{13}C-NMR spectrum of myrothelactone D (**8**) in DMSO-d_6

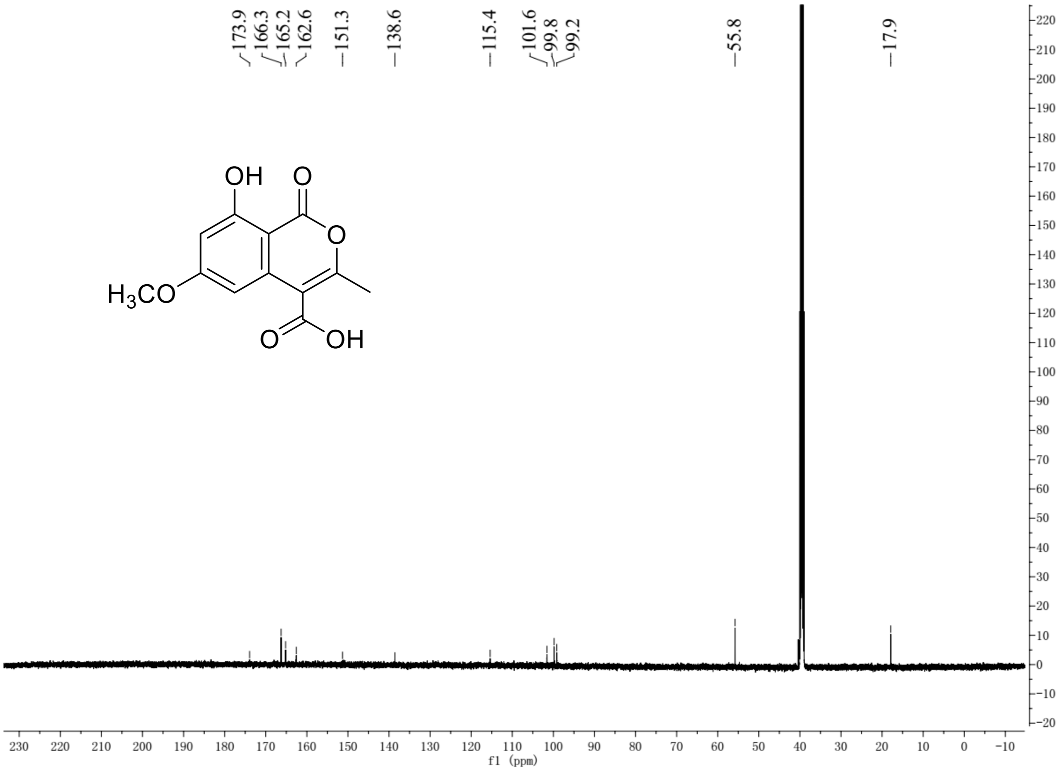

Figure S53. DEPT spectrum of myrothelactone D (**8**) in DMSO-d_6

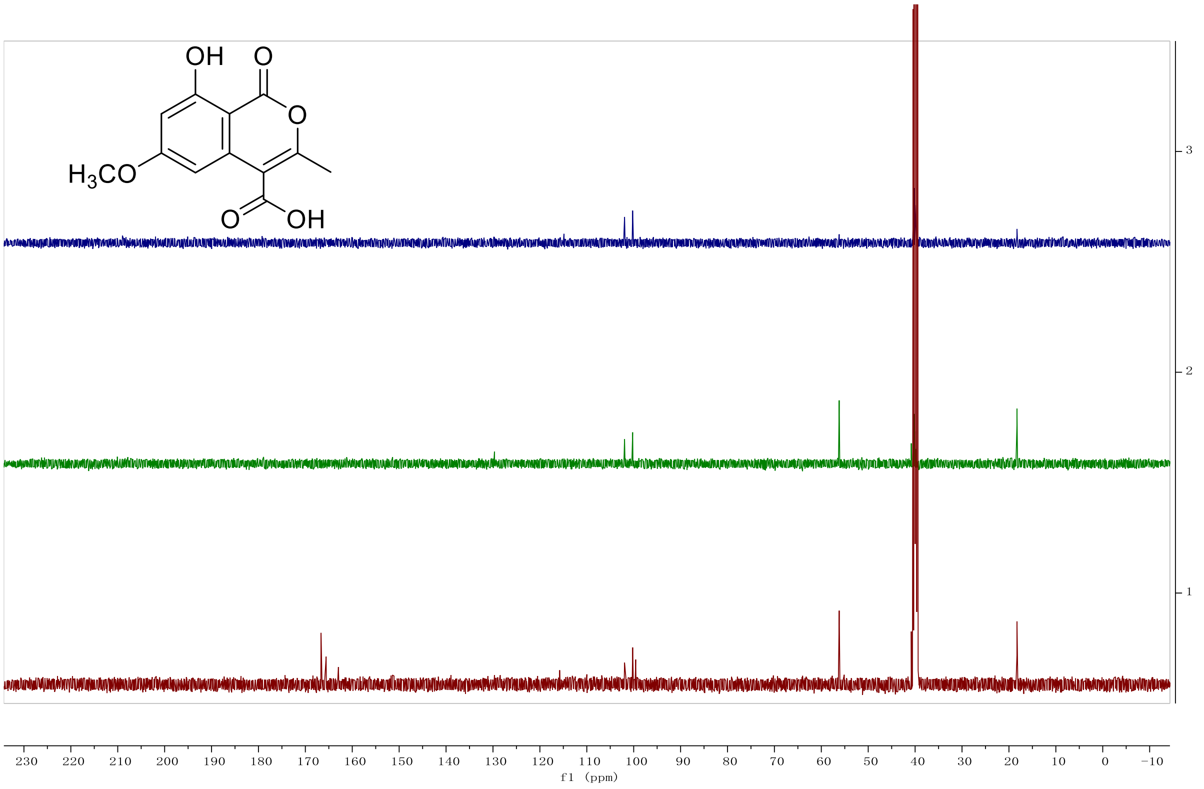

Figure S54. HSQC spectrum of myrothelactone D (**8**) in DMSO-d_6

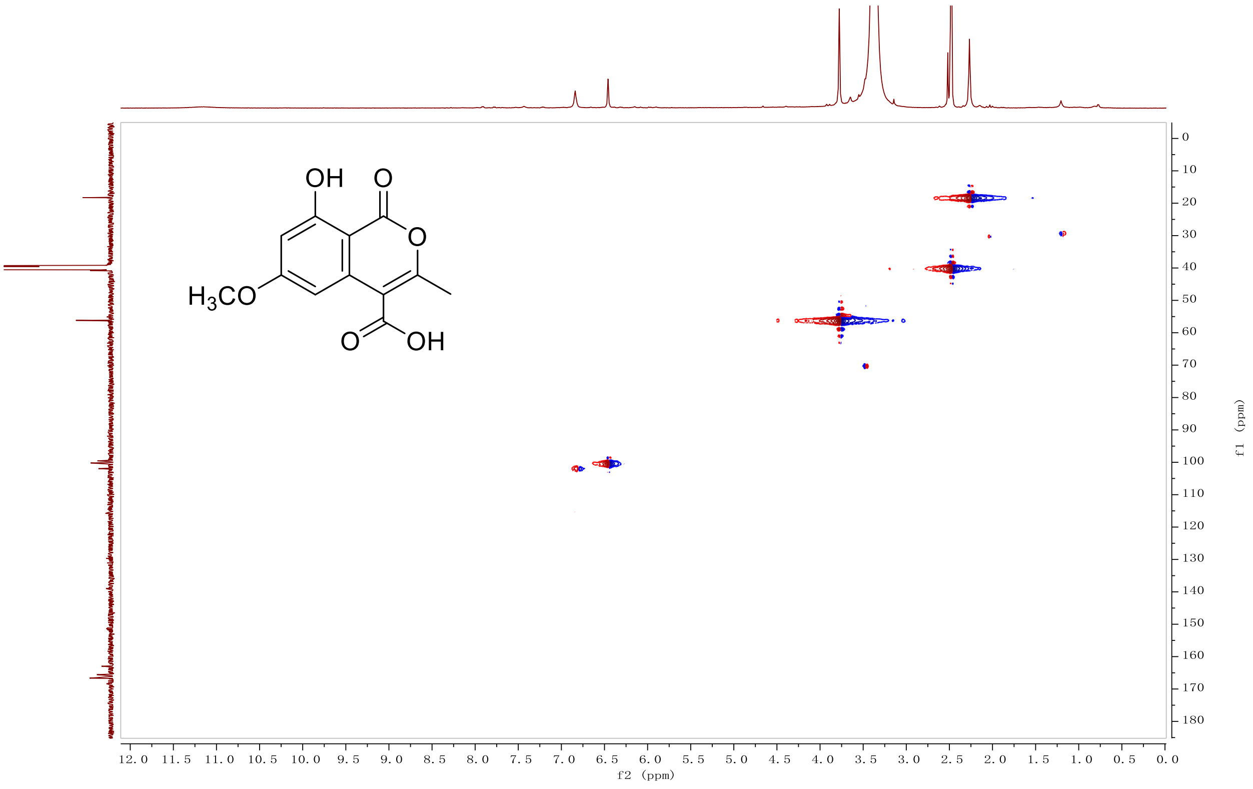

Figure S55. ^{1}H-^{1}H COSY spectrum of myrothelactone D (**8**) in DMSO-d_6

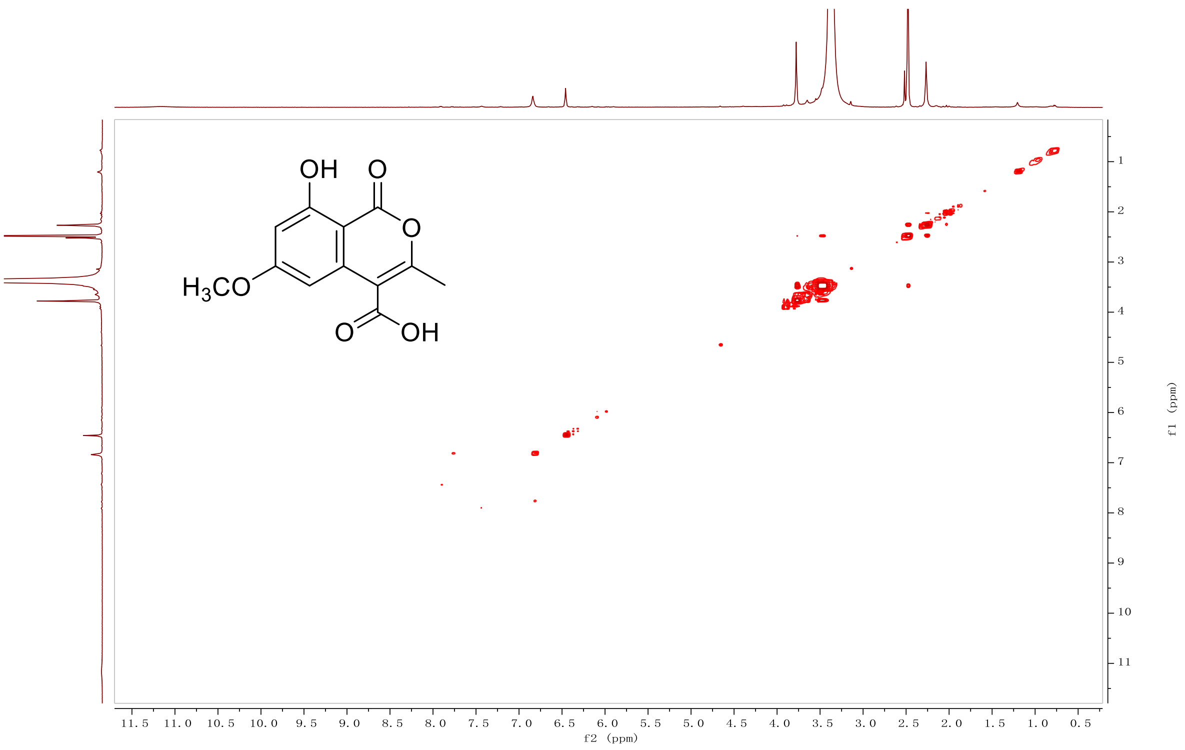

Figure S56. HMBC spectrum of myrothelactone D (**8**) in DMSO-d_6

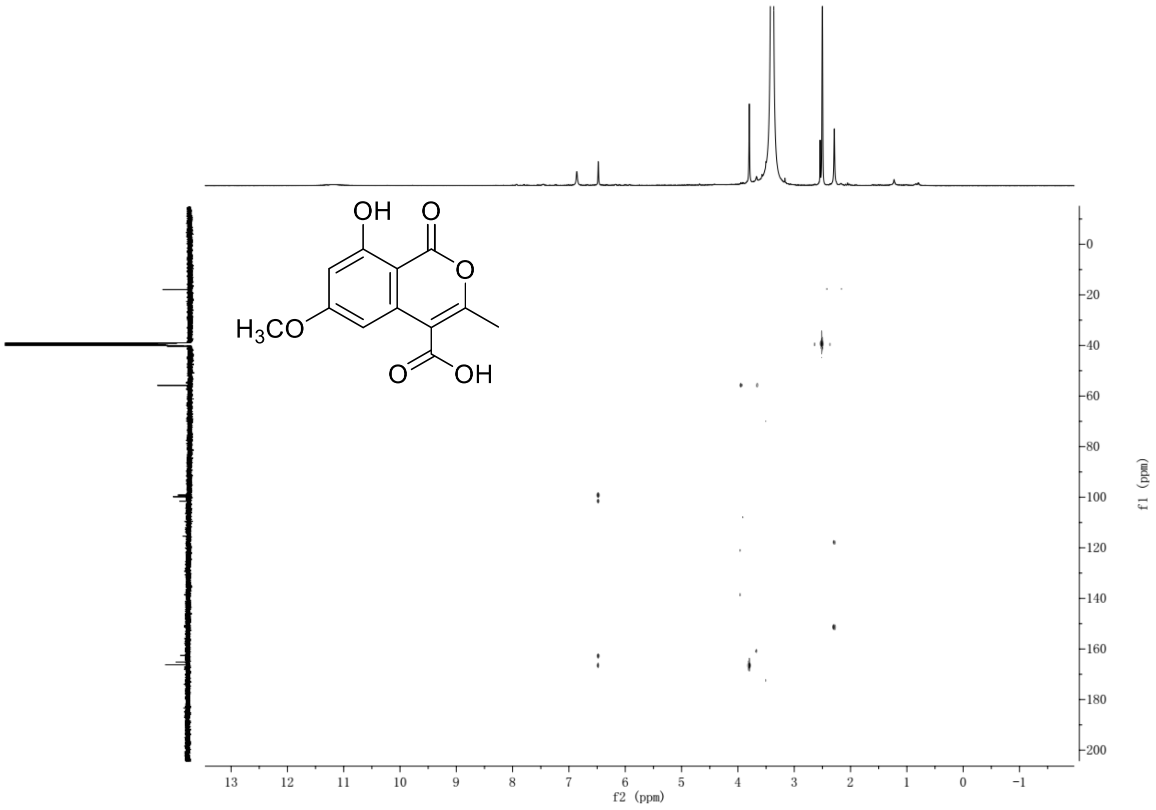

Figure S57. ^{1}H and ^{13}C NMR spectra of the known compounds **9–13** in DMSO-d_6

Figure S57A. ^{1}H NMR spectrum of compound **9** in DMSO-d_6

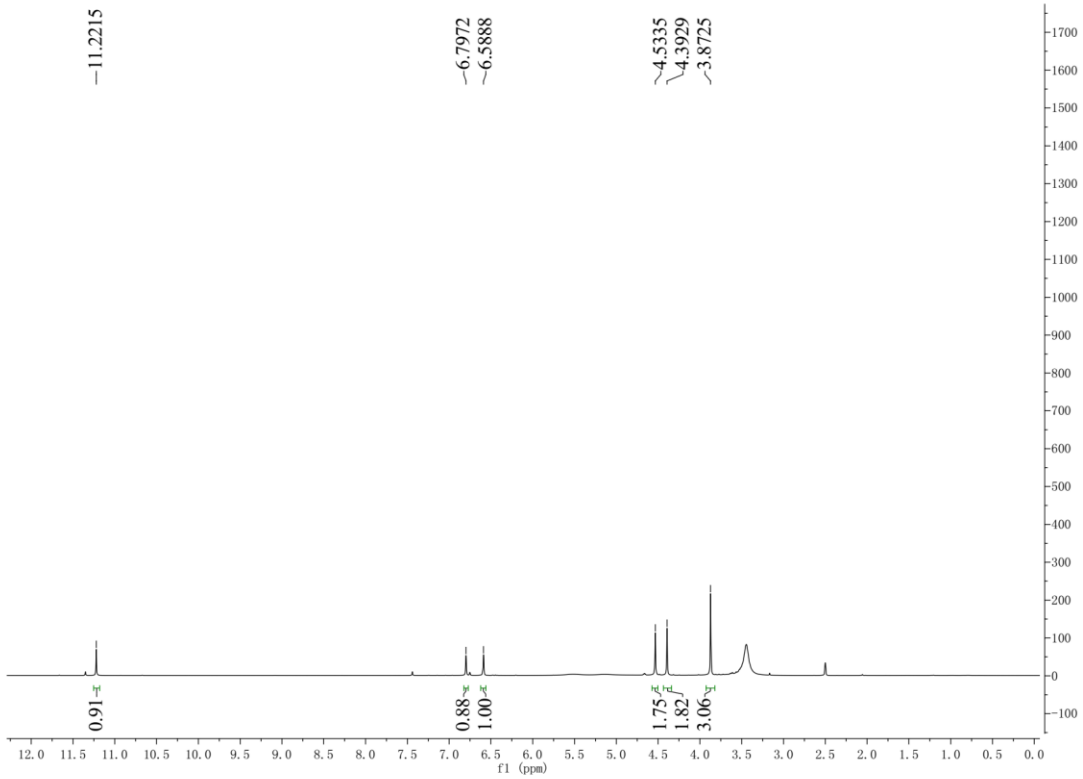

Figure S57B. ^{13}C NMR spectrum of compound **9** in DMSO-d_6

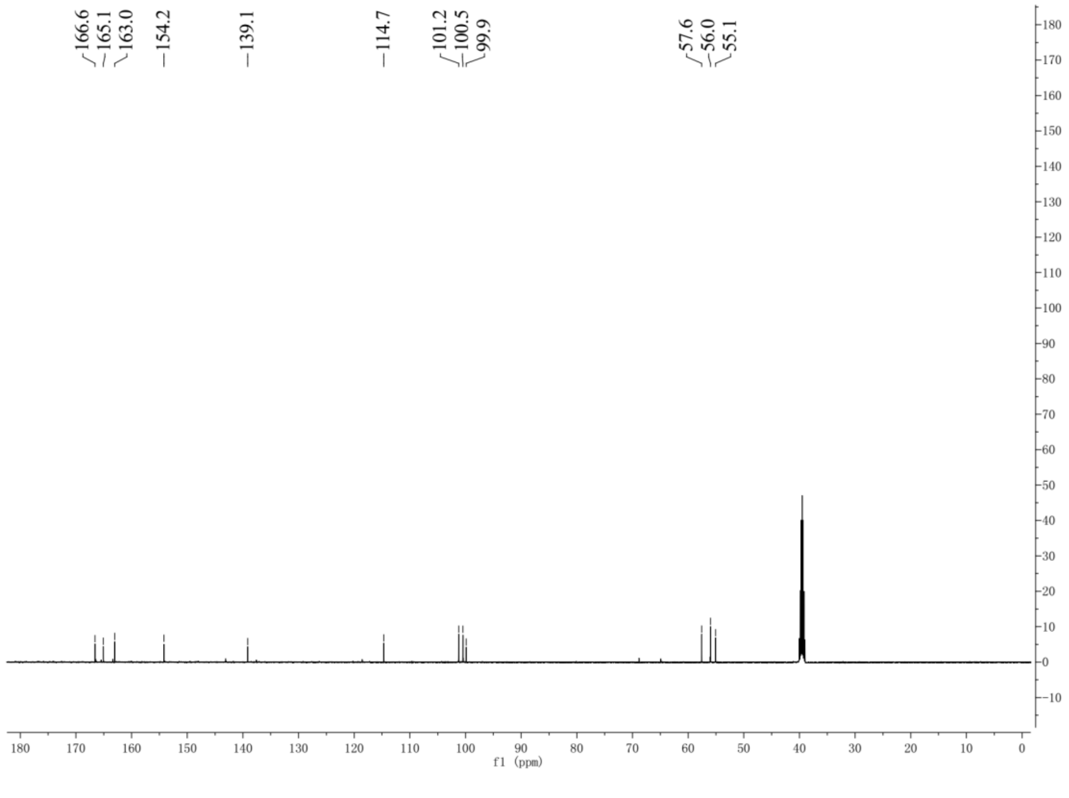

Figure S57C. ^{1}H NMR spectrum of compound **10** in DMSO-d_6

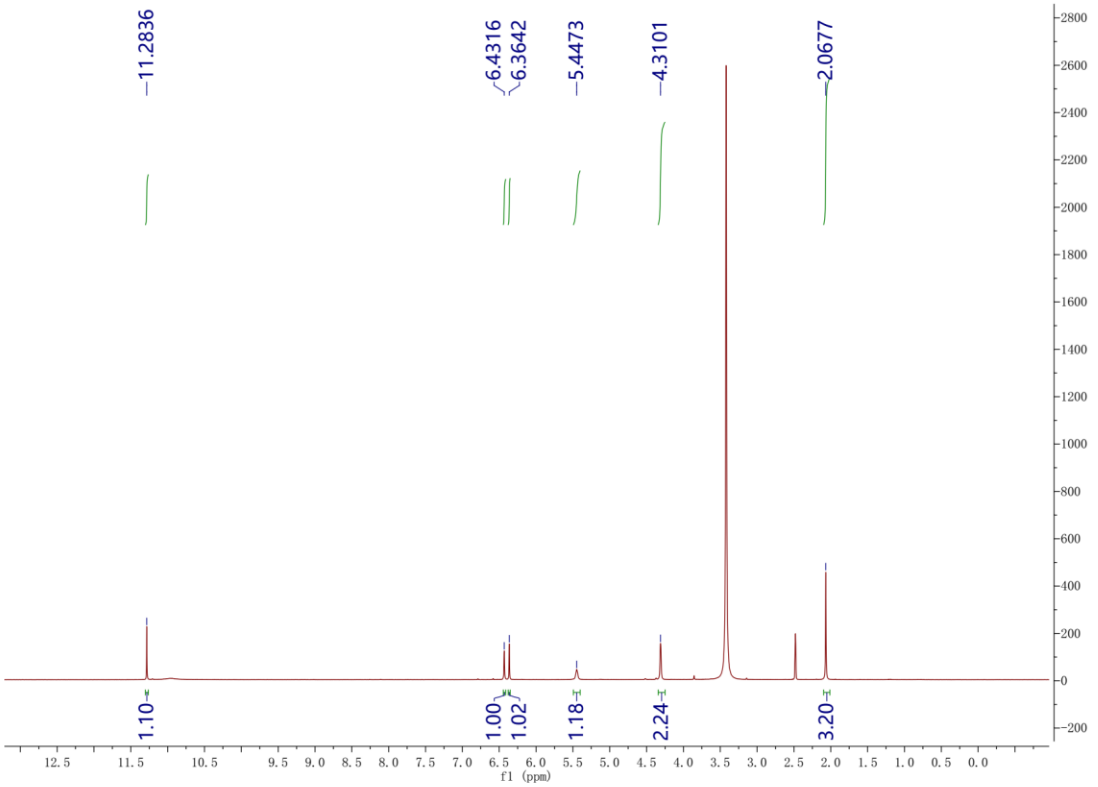

Figure S57D. ^{13}C NMR spectrum of compound **10** in DMSO-d_6

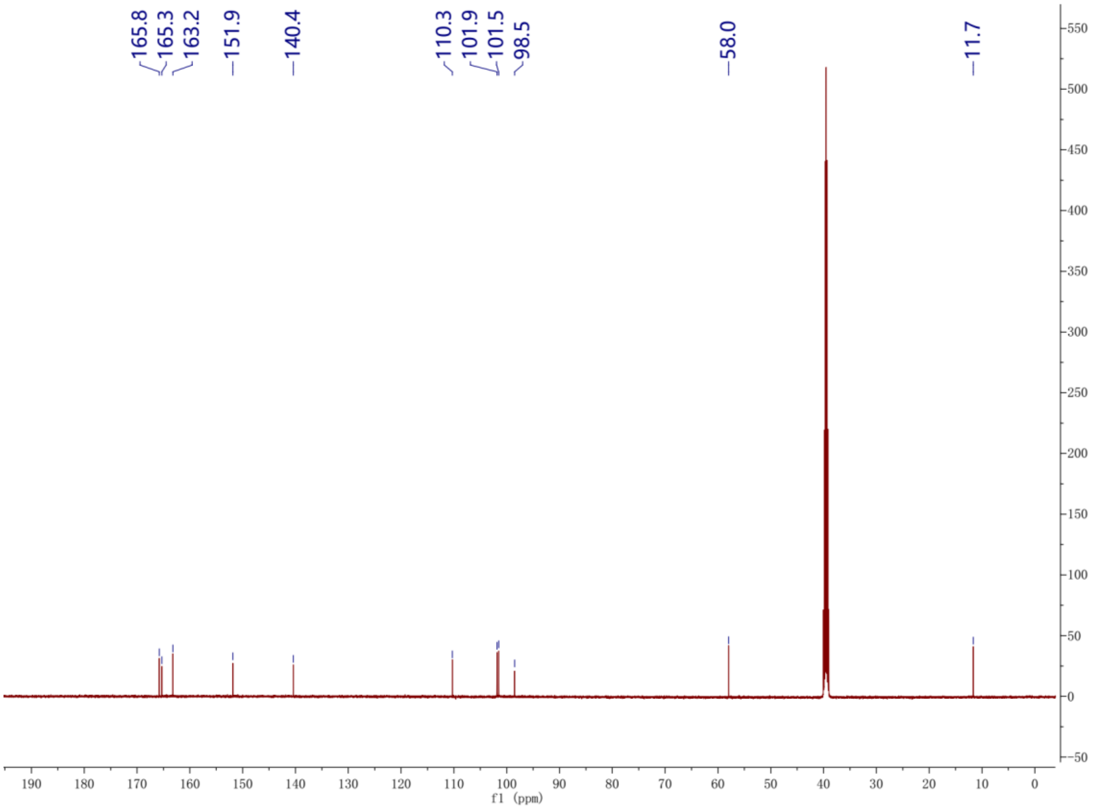

Figure S57E. ^{1}H NMR spectrum of compound **11** in DMSO-d_6

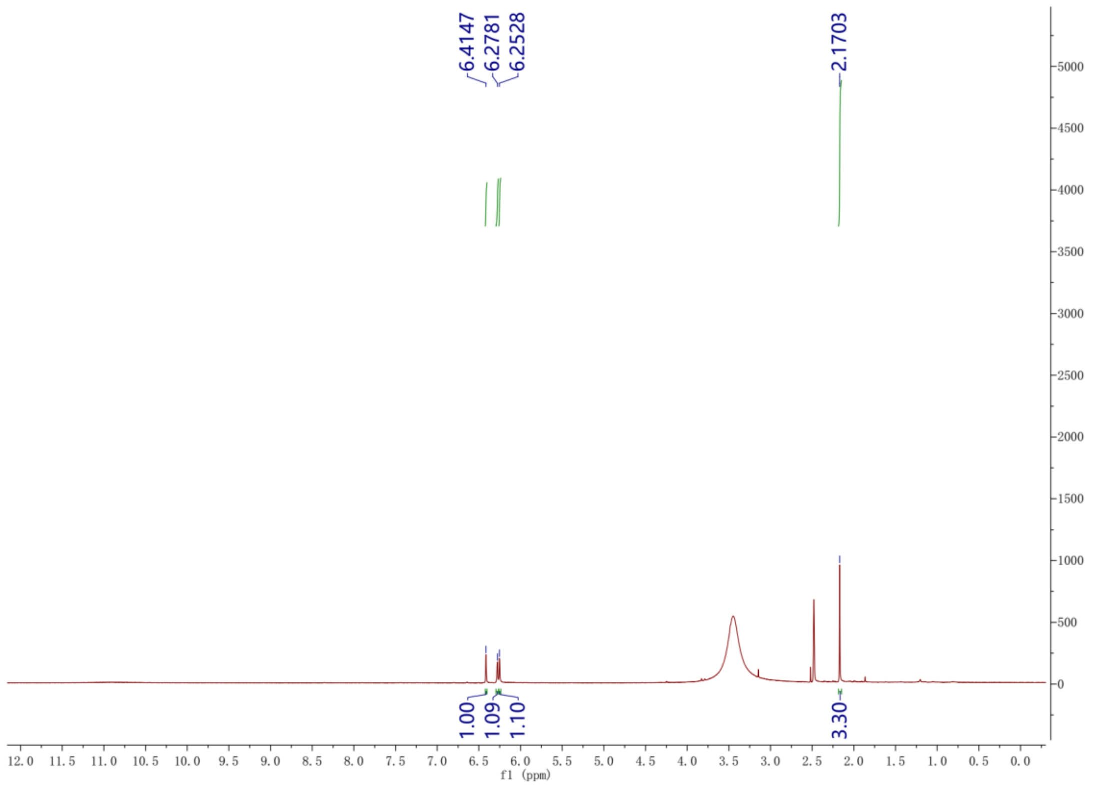

Figure S57F. ^{13}C NMR spectrum of compound **11** in DMSO-d_6

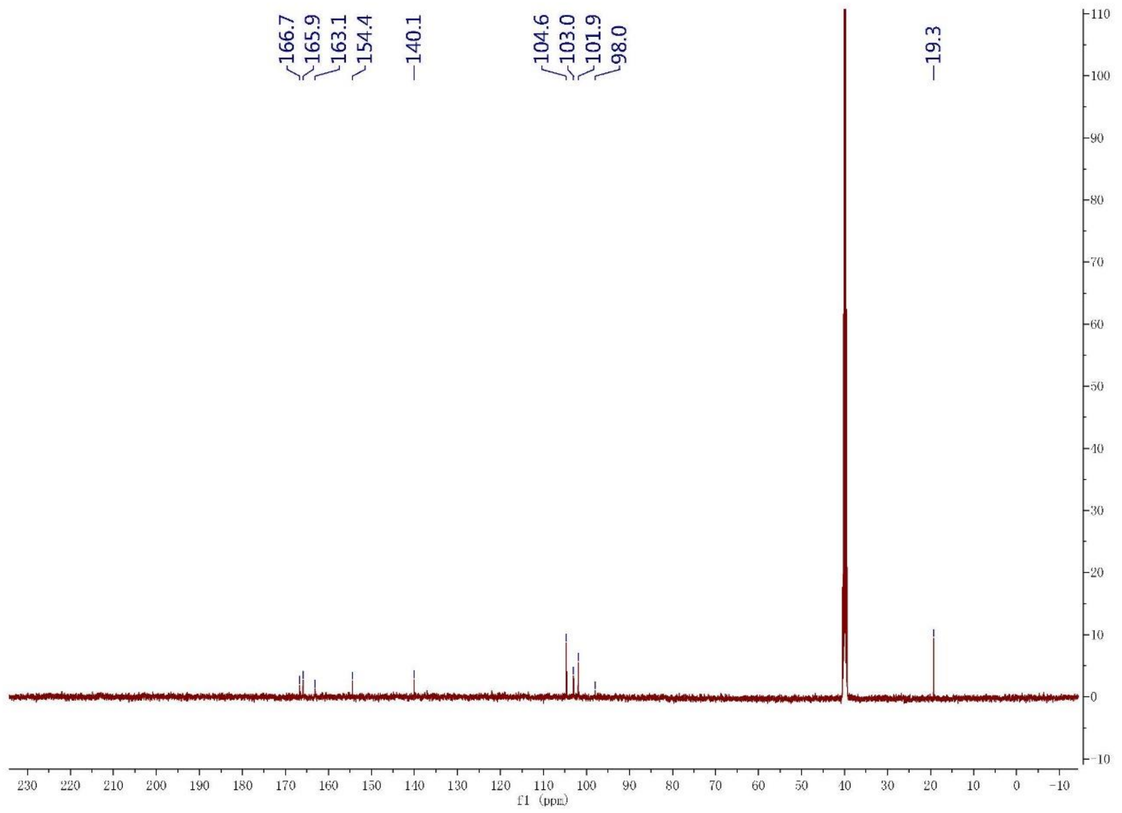

Figure S57G. ^{1}H NMR spectrum of compound **12** in DMSO-d_6

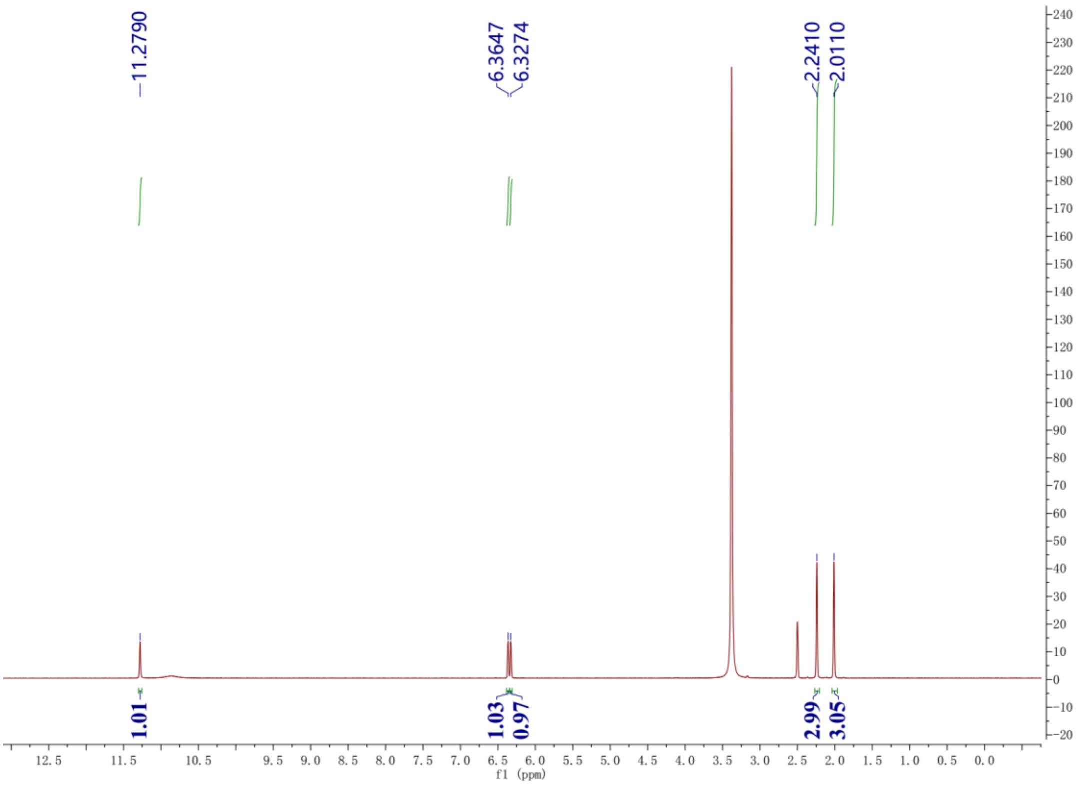

Figure S57H. ^{13}C NMR spectrum of compound **12** in DMSO-d_6

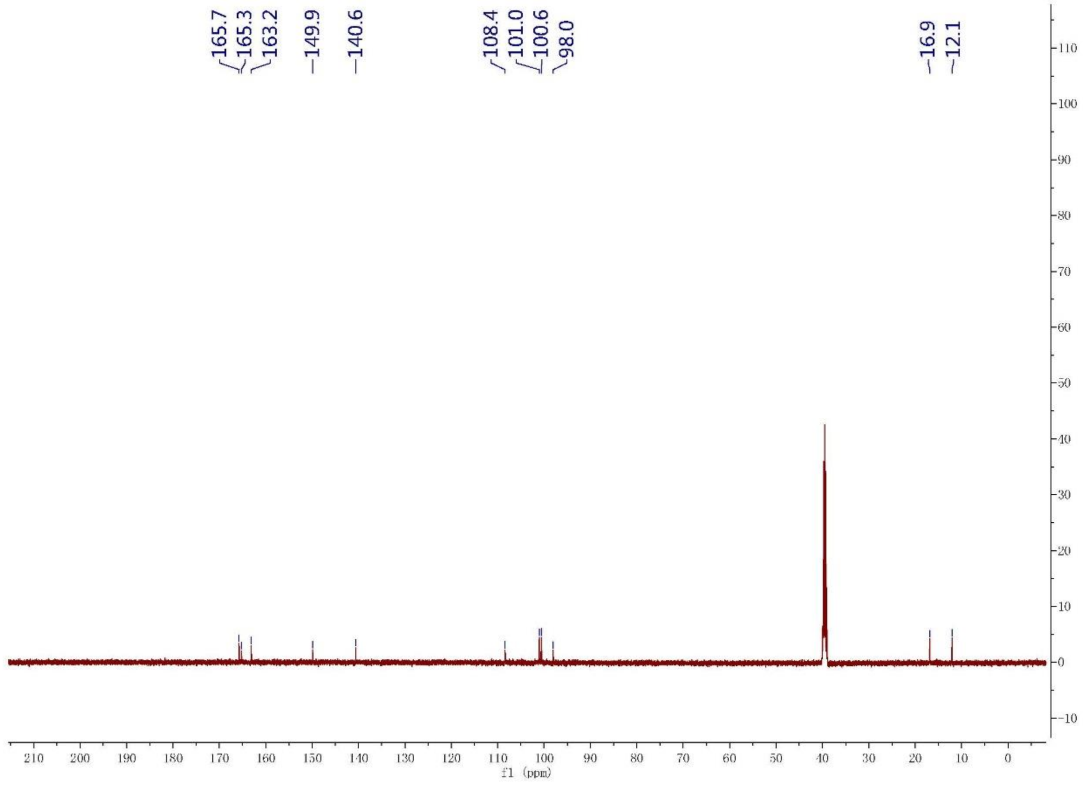

Figure S57I. ^{1}H NMR spectrum of compound **13** in DMSO-d_6

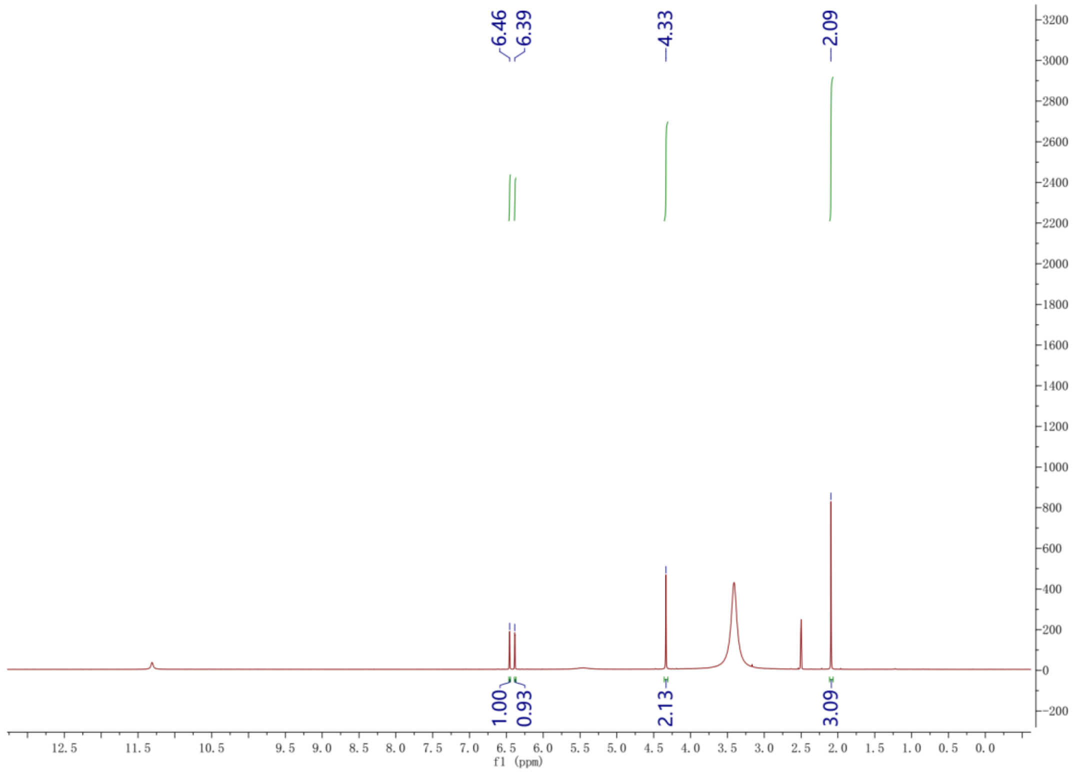

Figure S57J. ^{13}C NMR spectrum of compound **13** in DMSO-d_6

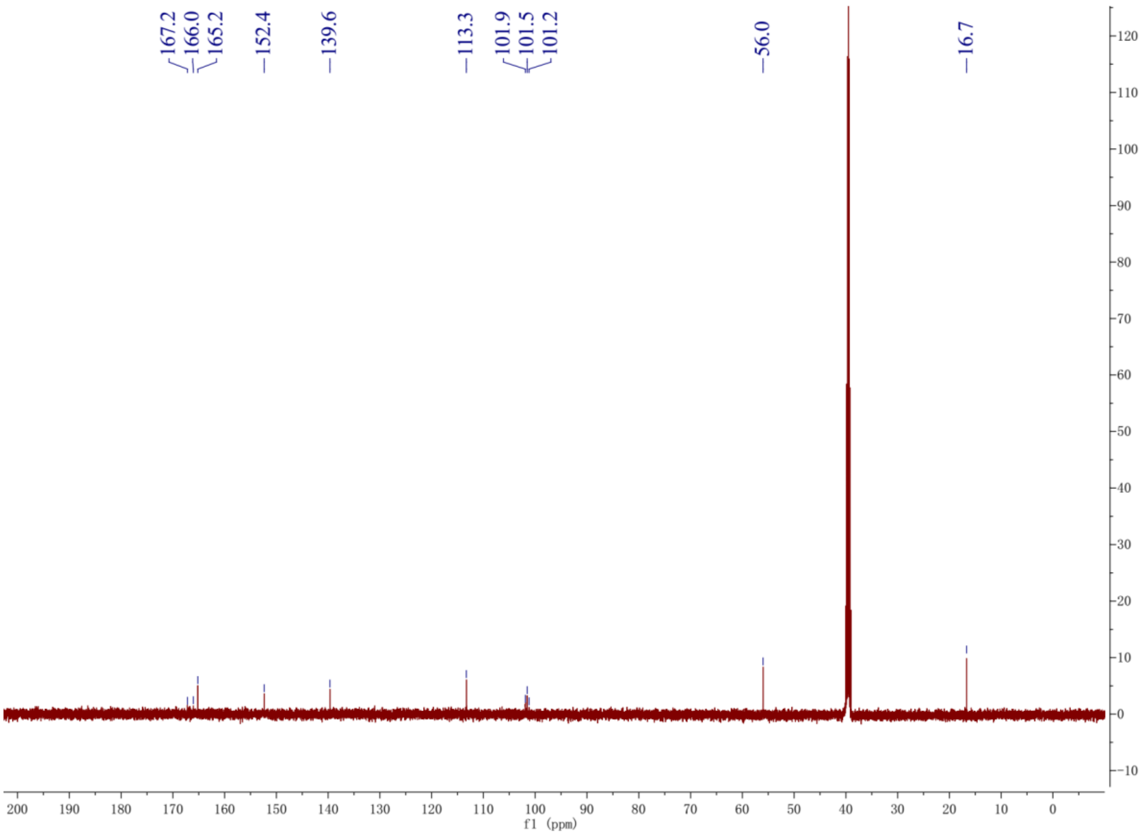

Figure S58. HRESIMS spectra of the new compounds **1–8**

Figure S58A. HRESIMS spectrum of compound **1**

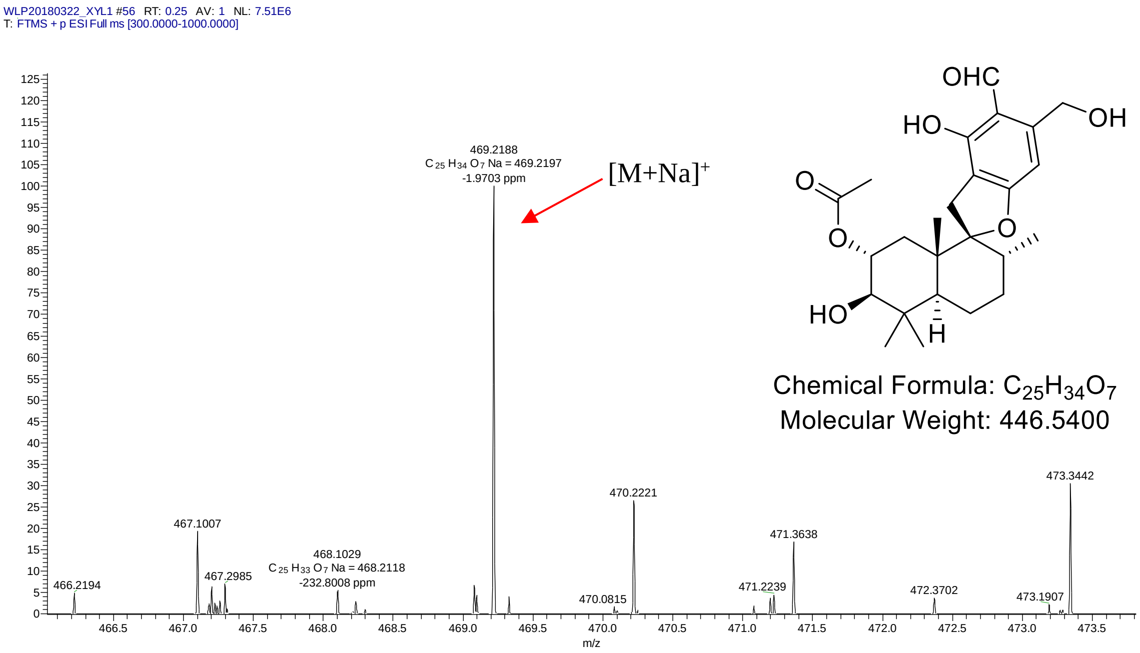

Figure S58B. HRESIMS spectrum of compound **2**

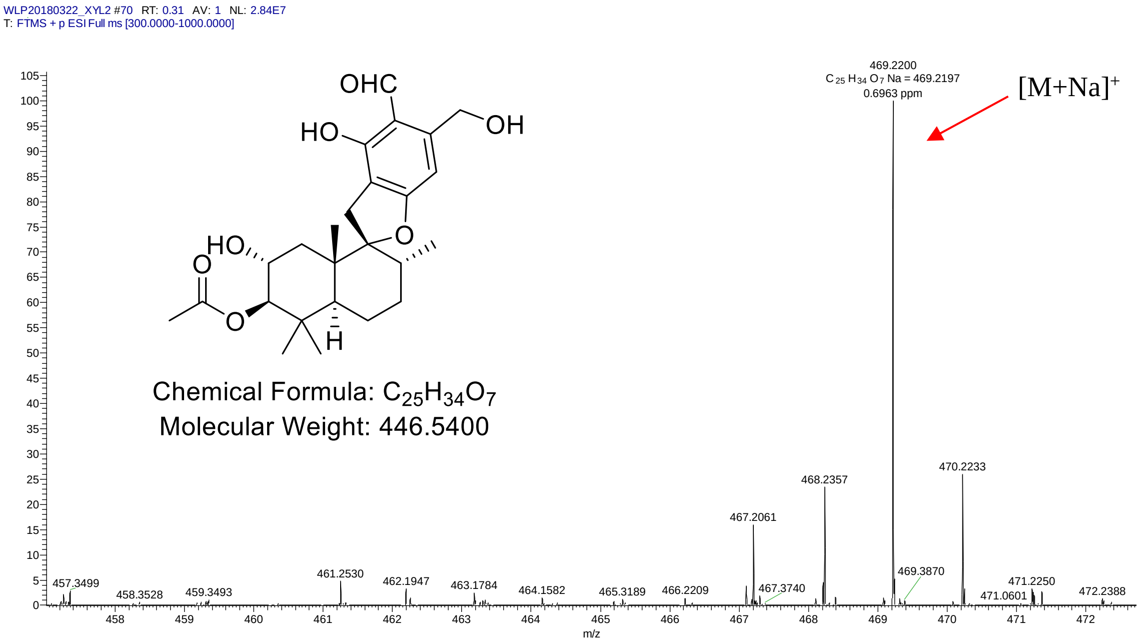

Figure S58C. HRESIMS spectrum of compound **3**

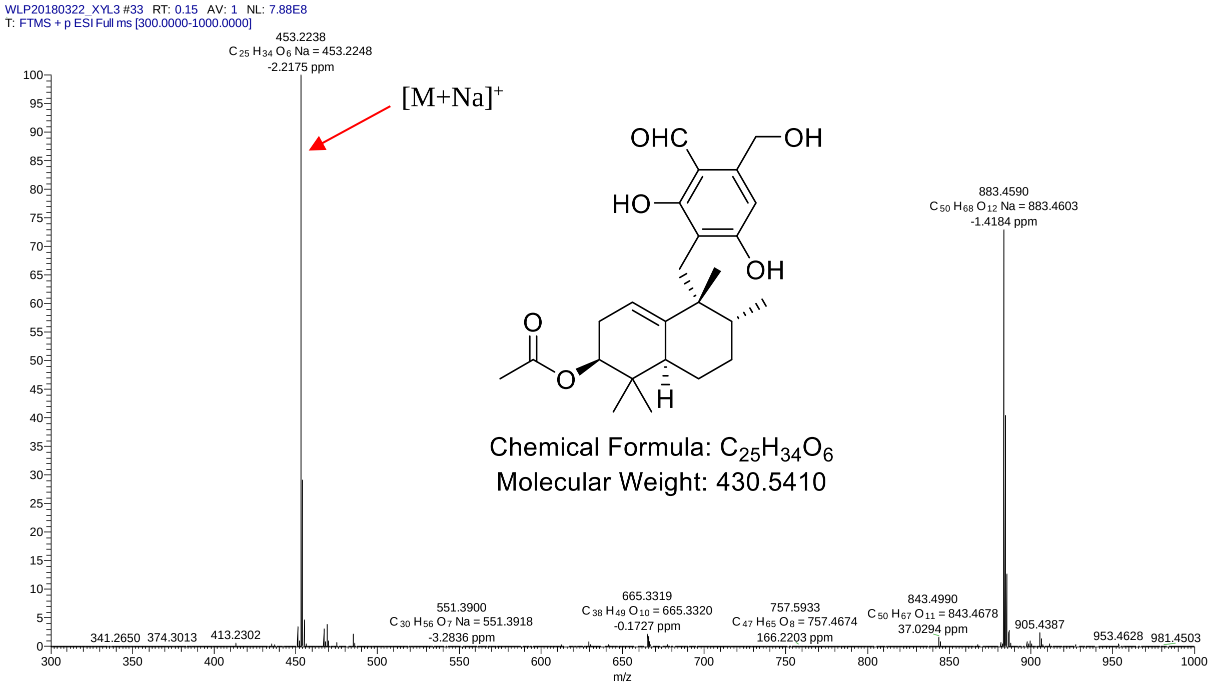

Figure S58D. HRESIMS spectrum of compound **4**

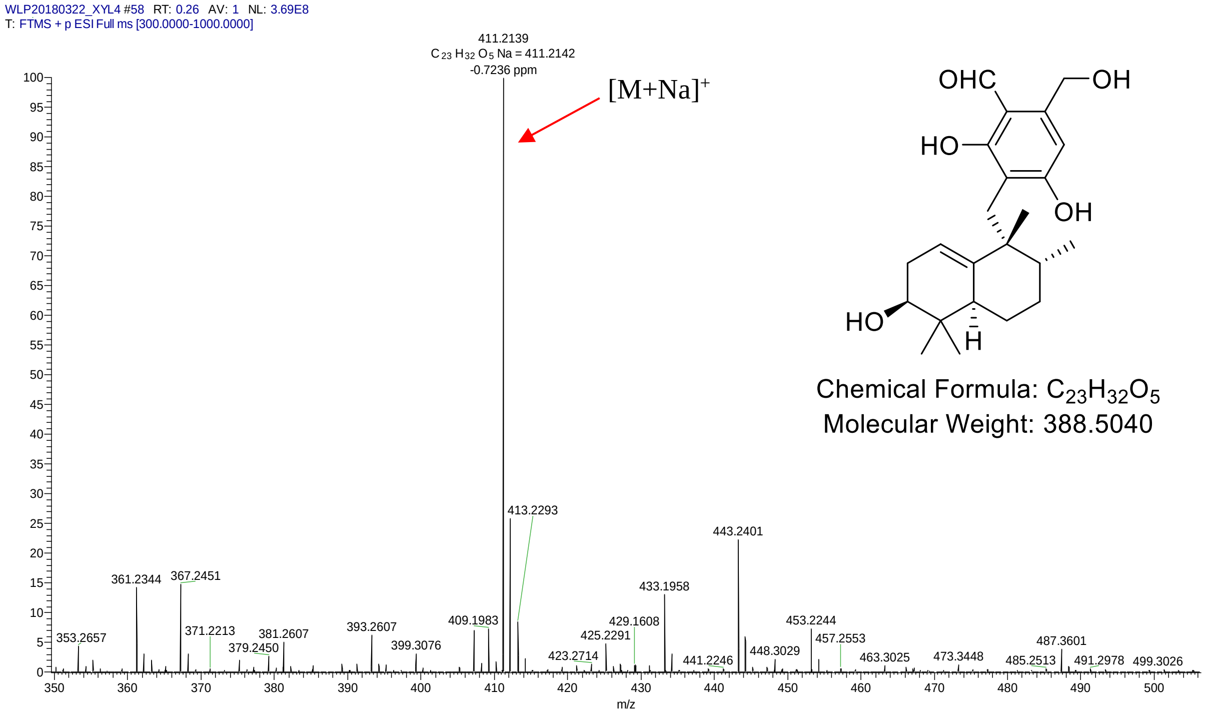

Figure S58E. HRESIMS spectrum of compound **5**

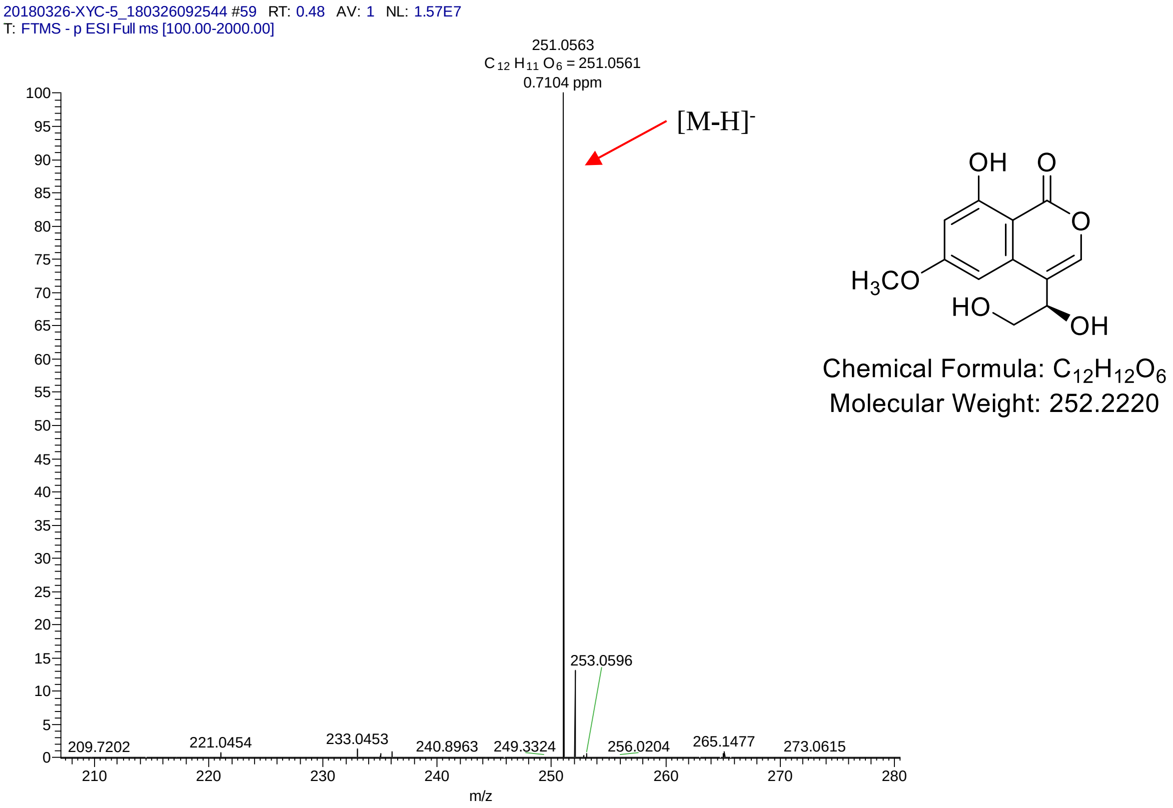

Figure S58F. HRESIMS spectrum of compound **6**

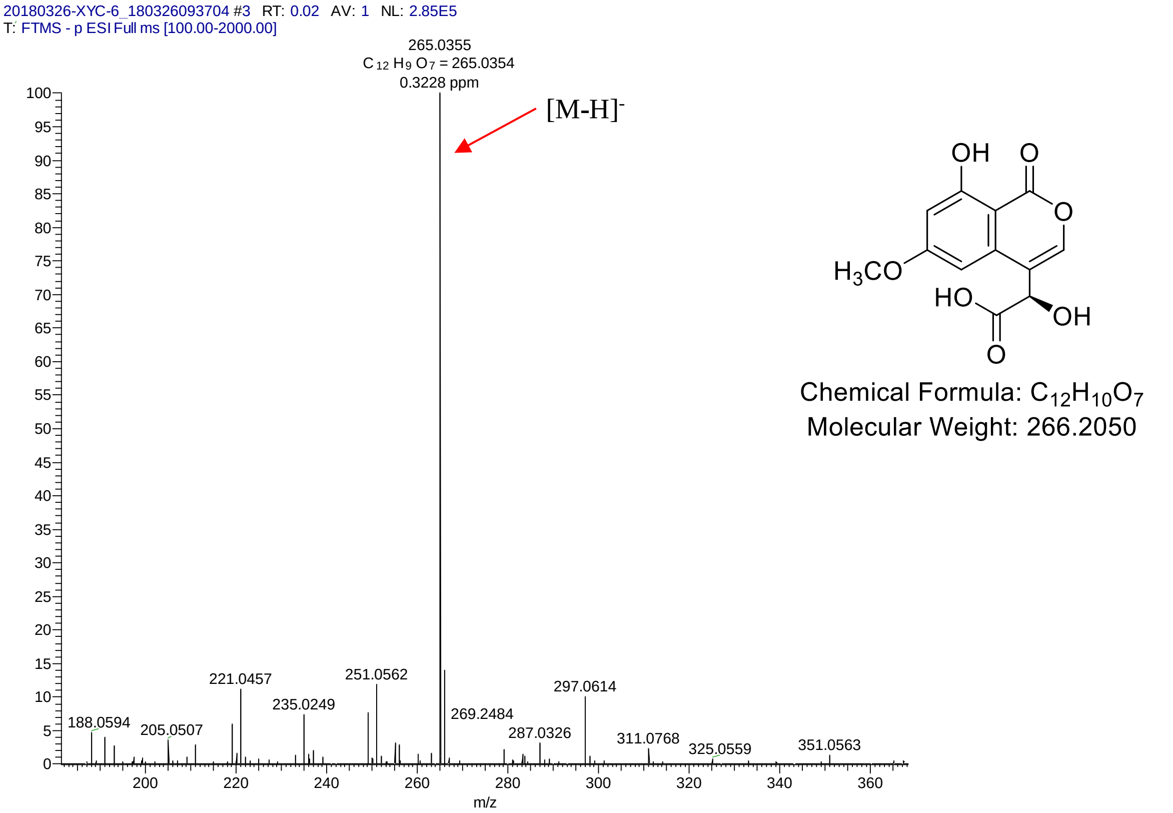

Figure S58G. HRESIMS spectrum of compound **7**

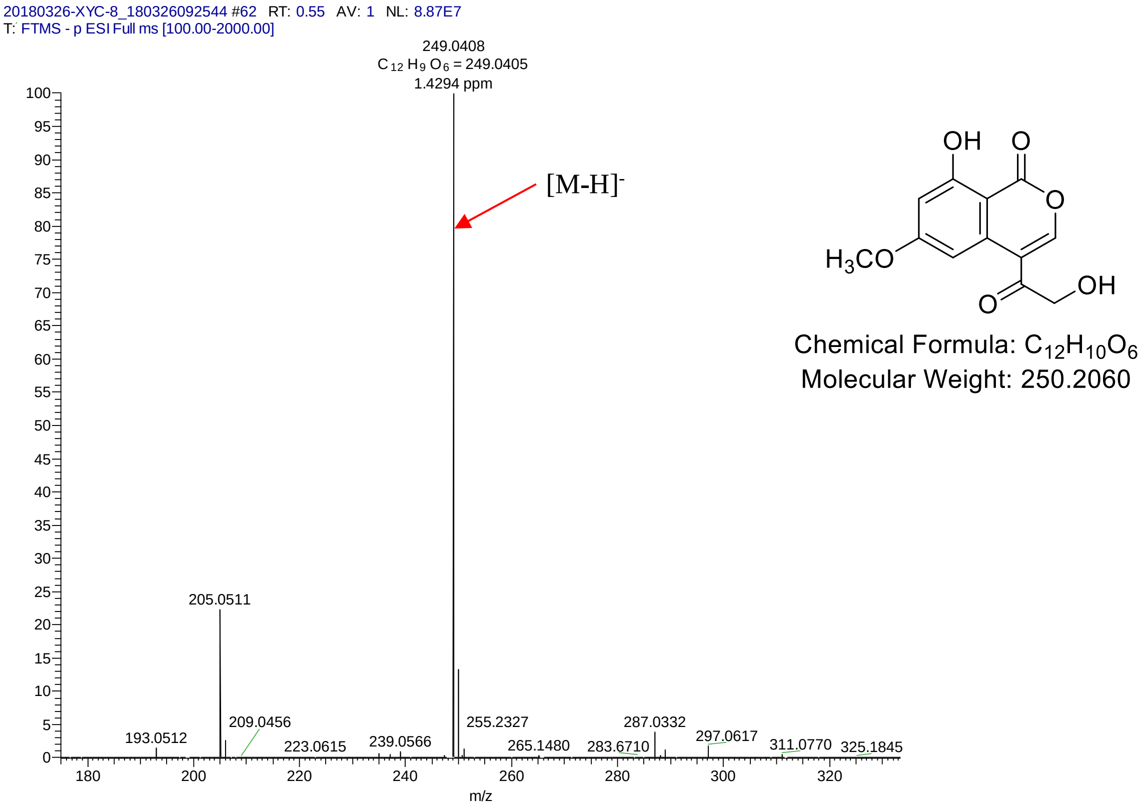

Figure S58H. HRESIMS spectrum of compound **8**

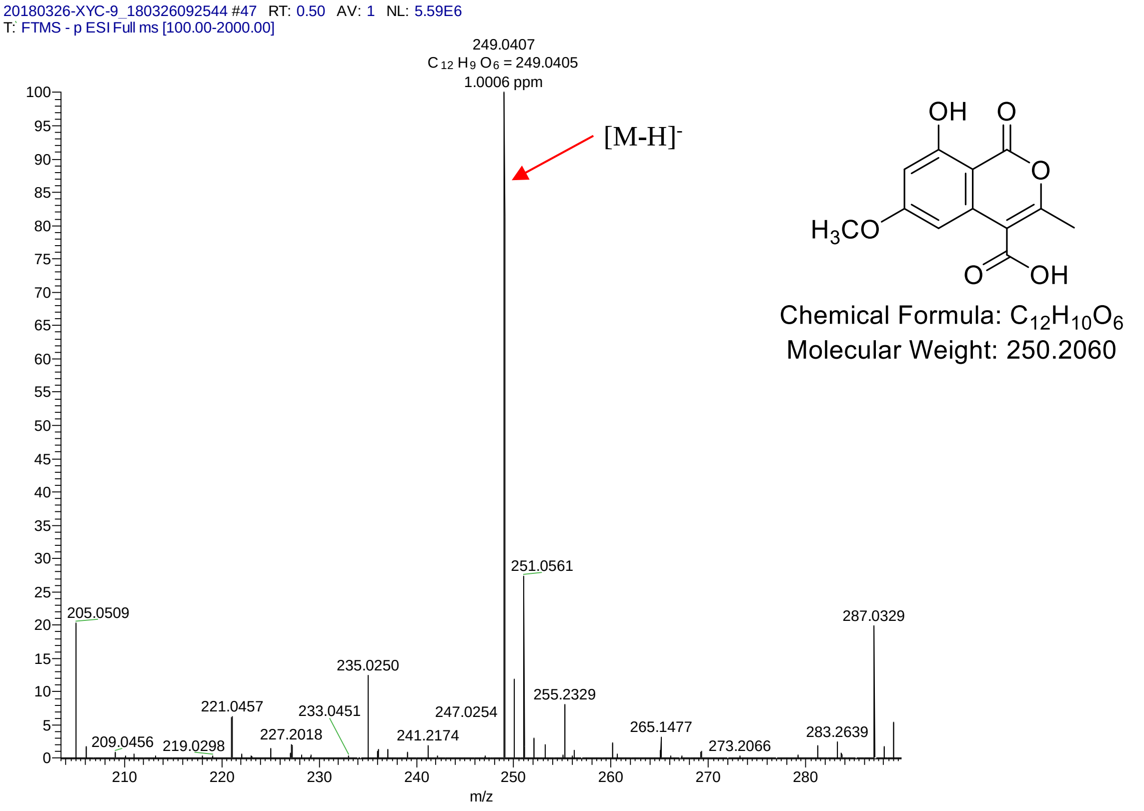

Figure S59. ESIMS spectra of the known compounds **9–13**

Figure S59A. ESIMS spectrum of compound **9**

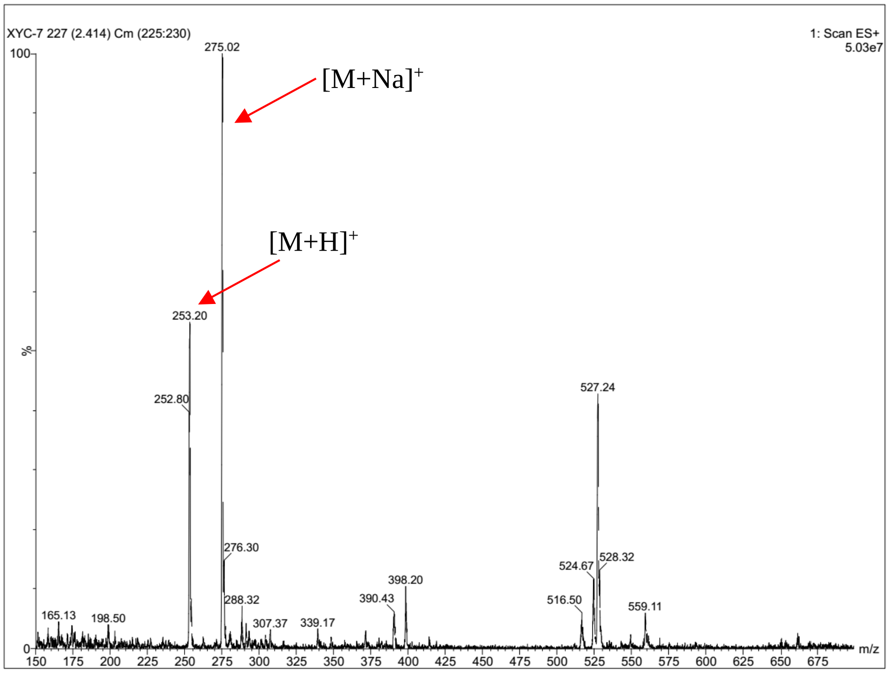

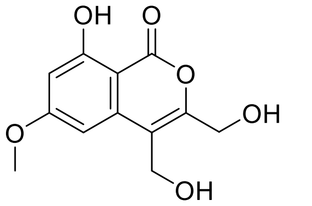

Chemical Formula: $C_{12}H_{12}O_6$
Molecular Weight: 252.22

Figure S59B. ESIMS spectrum of compound **10**

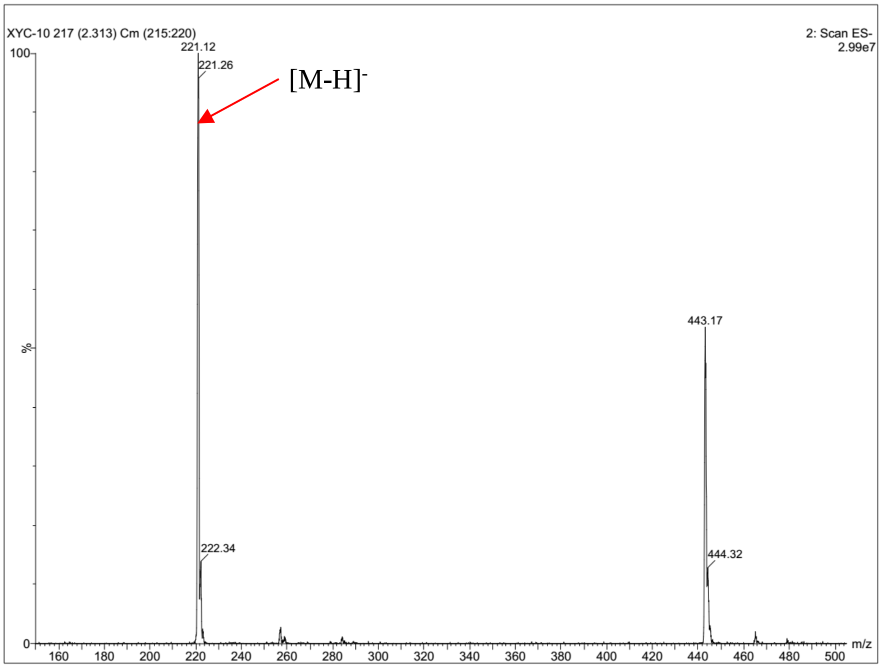

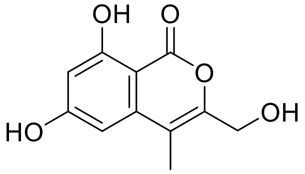

Chemical Formula: $C_{11}H_{10}O_5$
Molecular Weight: 222.20

Figure S59C. ESIMS spectrum of compound **11**

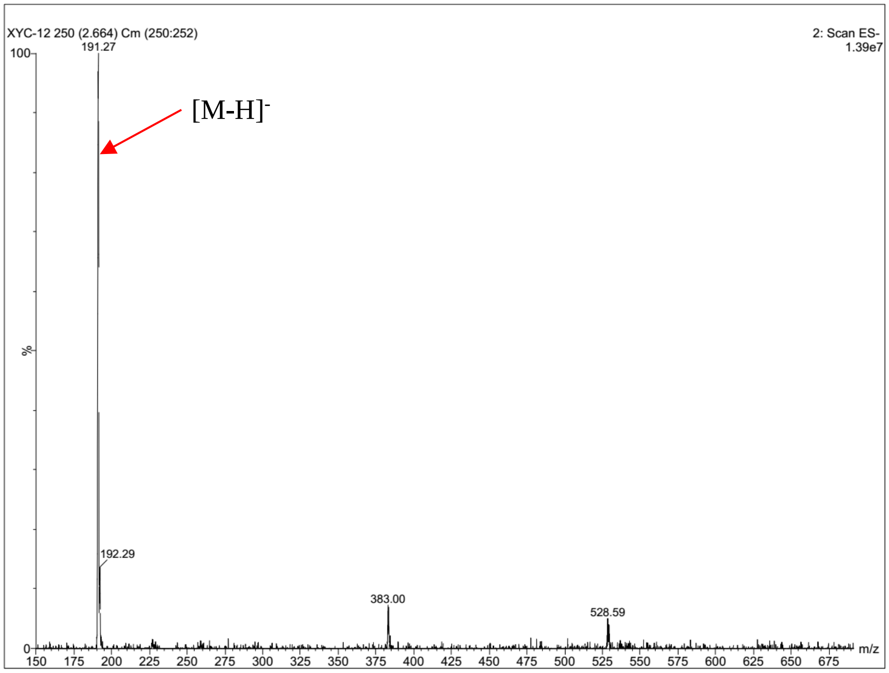

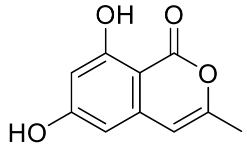

Chemical Formula: $C_{10}H_8O_4$
Molecular Weight: 192.17

Figure S59D. ESIMS spectrum of compound **12**

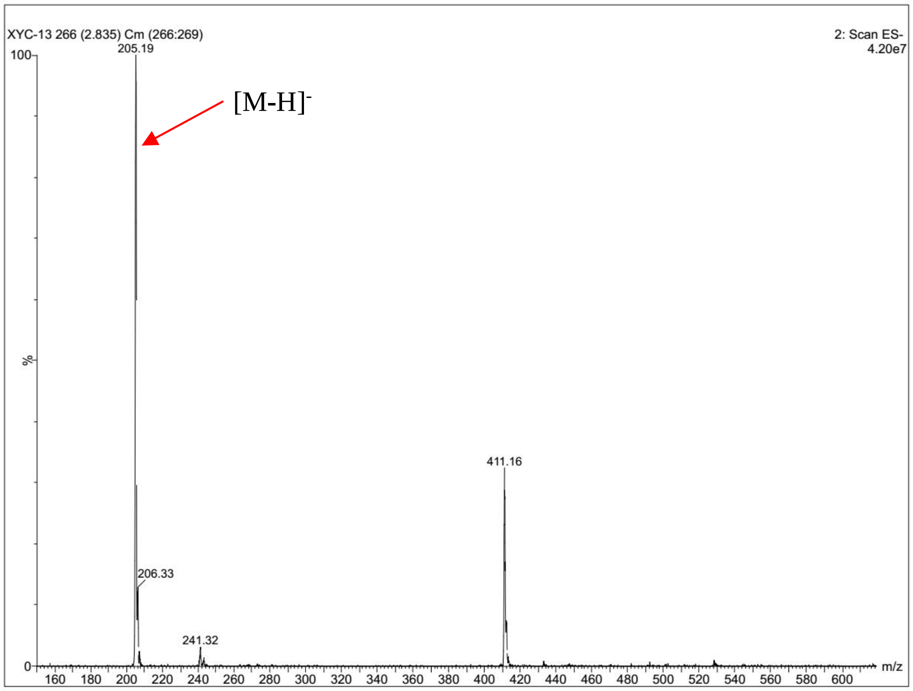

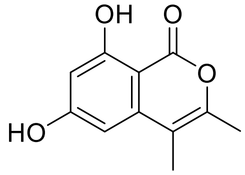

Chemical Formula: $C_{11}H_{10}O_4$
Molecular Weight: 206.20

Figure S59E. ESIMS spectrum of compound **13**

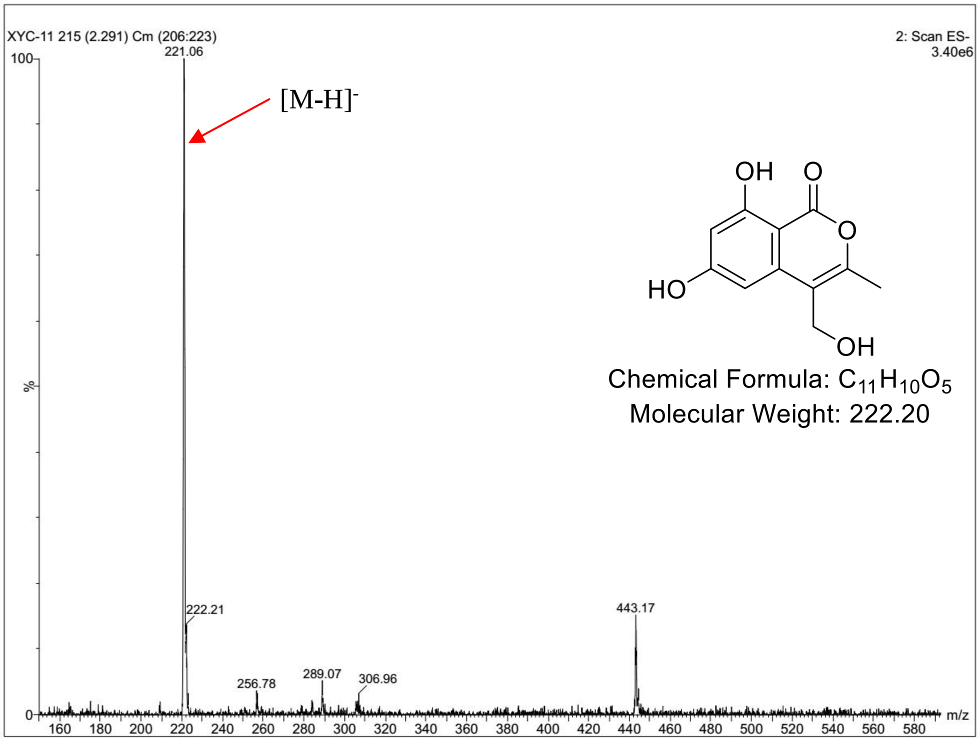

Table S1 ^{1}H and ^{13}C NMR data for the known compounds **9–13** in DMSO-d_6.

No.	9		10		11		12		13	
	δ_C	δ_H, Mult. (J in Hz)	δ_C	δ_H, Mult. (J in Hz)	δ_C	δ_H, Mult. (J in Hz)	δ_C	δ_H, Mult. (J in Hz)	δ_C	δ_H, Mult. (J in Hz)
1	165.1, C		165.3, C		165.9, C		165.3, C		166.0, C	
3	154.2, C		151.9, C		154.4, C		149.9, C		152.4, C	
4	99.9, C		110.3, C		104.6, CH	6.25, s	108.4, C		113.3, C	
5	101.2, CH	6.80, s	101.9, CH	6.43, s	103.0, CH	6.41, s	101.0, CH	6.36, s	101.9, CH	6.45, s
6	166.6, C		165.8, C		166.7, C		165.7, C		167.2, C	
7	100.5, CH	6.59, s	101.5, CH	6.36, s	101.9, CH	6.28, s	100.6, CH	6.33, s	101.5, CH	6.38, s
8	163.1, C		163.2, C		163.1, C		163.2, C		165.2, C	
9	139.1, C		140.4, C		140.1, C		140.6, C		139.6, C	
10	114.7, C		98.5, C		98.0, C		98.0, C		101.2, C	
11	55.1, CH_2	4.53, s	58.0, CH_2	4.31, s	19.3, CH_3	2.17, s	16.9, CH_3	2.24, s	56.0, CH_2	4.33, s
12	57.6, CH_2	4.39, s	11.7, CH_3	2.06, s			12.1, CH_3	2.01, s	16.7, CH_3	2.09, s
6-OCH_3	56.0, CH_3	3.87, s								
